# The IMPACT survey: a mixed methods study to understand the experience of children, adolescents and adults with osteogenesis imperfecta and their caregivers

**DOI:** 10.1186/s13023-024-03126-9

**Published:** 2024-03-21

**Authors:** Ingunn Westerheim, Tracy Hart, Taco van Welzenis, Lena Lande Wekre, Oliver Semler, Cathleen Raggio, Michael B. Bober, Maria Rapoport, Samantha Prince, Frank Rauch

**Affiliations:** 1 Osteogenesis Imperfecta Federation Europe, Heffen, Belgium; 2 Osteogenesis Imperfecta Foundation, Gaithersburg, MD USA; 3 TRS National Resource Center for Rare Disorders, Sunnaas Rehabilitation Hospital, Bjørnemyr, Norway; 4 University of Cologne, Cologne, Germany; 5 Hospital for Special Surgery, New York, USA; 6 Alfred I. duPont Hospital, Wilmington, USA; 7 Wickenstones Ltd., Abingdon, UK; 8 McGill University, Montreal, Canada

**Keywords:** Osteogenesis imperfecta, Patient reported outcomes, Survey, Burden of disease, Children, Adults, Adolescents, Impact, Rare diseases, Mixed methods

## Abstract

**Background:**

Osteogenesis imperfecta (OI) is a rare, heritable connective tissue disorder associated with a variety of symptoms, that affect individuals’ quality of life (QoL) and can be associated with increased healthcare resource use. While some aspects of OI are well studied, others remain poorly understood. Therefore, the IMPACT survey aimed to elucidate the humanistic, clinical and economic burden of OI on individuals with OI, their families, caregivers and wider society.

**Methods:**

We developed an international mixed methods online survey in eight languages (fielded July–September 2021), aimed at adults (aged ≥ 18 years) or adolescents (aged ≥ 12–17 years) with OI, caregivers (with or without OI) of individuals with OI and other close relatives. All respondents provided data on themselves; caregivers additionally provided data on individuals in their care by proxy. Data were cleaned, coded, and analysed using the pandas Python software package and Excel.

**Results:**

IMPACT collected 2208 eligible questionnaires (covering 2988 individuals of whom 2312 had OI) including 1290 non-caregiver adults with OI, 92 adolescents with OI, 150 caregiver adults with OI, 560 caregivers for individuals with OI, 116 close relatives and 780 proxy care-recipients with OI. Most individuals with OI (direct or proxy) described their OI as moderate (41–52% across populations) and reported OI type 1 (33–38%). Pain (72–82%) was the most reported clinical condition experienced in the past 12 months and was also most frequently rated as severely or moderately impactful. Further, among adults, 67% reported fatigue, 47% scoliosis, and 46% sleep disturbance; in adolescents, fatigue affected 65%, scoliosis and other bone problems 60%, and mental health problems 46%; in children, fractures were common in 67%, fatigue in 47%, and dental problems in 46%.

**Conclusion:**

IMPACT has generated an extensive dataset on the experience of individuals with OI, their caregivers and relatives. We found that, irrespective of age, individuals with OI experience numerous and evolving symptoms that affect their QoL; however, pain and fatigue are consistently present. Upcoming analyses will provide further insights into the economic impact, healthcare journey and caregiver wellbeing, aiming to contribute to improved treatment and care for the OI community.

**Supplementary Information:**

The online version contains supplementary material available at 10.1186/s13023-024-03126-9.

## Introduction

Osteogenesis imperfecta (OI) is a rare, heritable connective tissue disorder with variable manifestations and numerous symptoms affecting 1–5 in 10,000 individuals [1–3]. Most often the condition is caused by alterations in the type 1 collagen genes (e.g. COL1A1 and COL1A2), but mutations in other genes that are linked to the collagen synthesis are also associated with OI [1]. OI affects multiple systems and organs in the body, resulting in an array of possible symptoms including fractures, skeletal deformities, pain, joint hypermobility and occasionally blue sclerae, hearing loss, dental abnormalities, basilar invagination, cardiovascular and pulmonary abnormalities [1].

Currently there are no curative therapies for OI, and treatment primarily aims to manage and decrease fractures, pain, and bone deformities, and promote mobility and independence. To manage the spectrum of OI symptoms multi-disciplinary care is required [4, 5]. Most therapeutic agents used in OI care were initially developed to target the bone metabolism in conditions such as osteoporosis and are used off-label [4]. Bisphosphonates are most widely used; additionally human monoclonal antibodies (e.g., denosumab) and parathyroid hormone (e.g., teriparatide) are available [4, 5].

Considering the lack of curative treatment and the condition’s effect on multiple systems and organs in the body, living with OI has a significant impact on the physical, social, and emotional wellbeing of individuals as well as their families and caregivers [6–8]. However, due to its rarity and variability, research into OI is challenging, and there are few comprehensive, self-reported patient outcome studies in this field [6, 9].

While some aspects of OI and their impact on health-related quality of life (HRQoL) are well-understood (e.g., mobility challenges and fractures) others, such as women’s health, characteristics of pain, treatment-related adverse events, pulmonological conditions, and gastrointestinal-, sleep-, and skin-related conditions are less well studied [9]. Similarly, the impact on some caregivers of individuals with OI is incompletely understood, particularly fathers and siblings [9]. In addition, as most HRQoL studies are cross-sectional and use small samples, our understanding of the HRQoL of individuals with OI at different life stages is limited [7, 9]

OI may also be associated with a considerable economic burden on individuals and their families, even in socialised healthcare systems [10, 11], and an increased use of healthcare and social service resources [12–17]. However, studies focus on the societal and healthcare resource use associated with OI, while out of pocket spending is rarely described [10, 11].

To better understand the humanistic, clinical and economic impact of OI on individuals with OI, their families, caregivers and wider society we conducted the IMPACT Survey (Living with osteogenesis imperfecta: understanding experiences based on community insight & evidence) aimed at individuals with OI, their caregivers and relatives. Here we present the study’s design, methodology and first findings, including demographics, clinical characteristics and clinical signs, symptoms and events and their impact.

## Methods

### Development

IMPACT was developed by a steering committee that included independent academic researchers, representatives of the patient advocacy organisation (PAO) Osteogenesis Imperfecta Foundation (OIF; USA), the umbrella PAO Osteogenesis Imperfecta Federation Europe (OIFE) and representatives of Mereo BioPharma.

Questions that could potentially address evidence gaps identified in a scoping literature review [9], that were most relevant to the OI and research community and most suitable to survey-based research, were prioritised. The questionnaire was drafted and reviewed in English and professionally translated into German, Italian, Dutch, French, Russian, Spanish (both South American and European) and Portuguese. The survey was extensively tested by the authors, OI community members and volunteers. Community members from Germany, Norway, the Netherlands, Italy, France, Spain, Mexico, Italy, Russia and the USA tested the survey for local relevance using Microsoft Word copies of the translated survey and advised on regionally relevant question wording and answer options. To this end, individuals were supplied with a Microsoft Word version of the survey as well as a test version of the online survey. Additionally the online version of the survey was tested using respondent profiles developed by the authors to ensure that the survey platform guided respondents as intended and all questions were clear and displayed correctly.

An ethics approval was not sought because exemption was granted by an independent review board (Pearl IRB, Indianapolis, USA).

### Population-specific questionnaire design

A basic version of the questionnaire was adapted to cover five main respondent populations. The “non-caregiver adult with OI” questionnaire was designed for individuals with OI ≥ 18 years of age who answered questions about themselves. This population is called “non-caregiver adults with OI” to distinguish from the adults with OI who identified as caregivers of care recipients with OI. The “caregiver adult with OI” questionnaire was designed for individuals with OI ≥ 18 years of age who were caregivers of care recipients with OI who answered questions about themselves, and by proxy, about individuals in their care (children [0–11 years], adolescents [12–17 years] or adults [≥ 18 years] with OI). The “adolescent with OI” questionnaire was designed for adolescents with OI aged 12–17 years who answered questions about themselves. The “caregiver without OI” questionnaire was designed for caregivers of individuals with OI who answered questions about themselves and, by proxy, about their care recipients with OI (children, adolescents or adults with OI). The “relative” questionnaire was designed for other relatives of individuals with OI (Table 1). For the “adolescent with OI” questionnaire, certain topics were removed (e.g., sexual health and financial impact), and the language was adjusted for ease of understanding. Questionnaires were adjusted for each population, to keep the survey size manageable for respondents who provided information on themselves and care recipients. The full questionnaires are provided in Additional file 1.

**Table 1:** IMPACT Survey design overview

	Population				
Domain	Non-caregiver adults with OI	Caregiver adults with OI^a, b^	Caregivers without OI^a^	Adolescents with OI	Relatives
Participation criteria	≥ 18 years of age	≥ 18 years of age OI Self-describes as someone who provides care for individuals with OI	≥ 18 years of age Self-describes as someone who provides care for individuals with OI	≥ 12–17 years of age OI	≥ 12 years of age Self-assesses relationship to person with OI as close but does not self-describe as a caregiver
N questions^c^	37–84	50–111	49–94	28–69	10–12
Clinical characteristics	Demographic data; for each person with OI: height, OI type, OI severity, causative gene, mobility status				Demographics
Clinical signs, symptoms and events	Signs, symptoms and events in past 12 months and lifetime				NA
Treatment and care experience	Diagnostic pathway, experience with healthcare providers, access to care				NA
QoL	Impact of OI on individuals(s) with OI, impact of signs, symptoms and events, worries				NA
Impact on families	NA	Impact on caregivers and impact on family life		NA	Impact of OI, worries
Healthcare consumption	Use of therapies in past 12 months and lifetime, use of inpatient and outpatient care, consumable use				NA
Financial sources for treatment	Insurance coverage, out of pocket spending			NA	NA

NA, not applicable; OI, osteogenesis imperfecta; QoL, quality of life

^a^Caregivers provided information on 1–3 care recipients with OI of any age; ^b^Caregivers with OI only provided information about their OI and their experience of caring for individuals with OI, they did not report about the OI experience of individuals in their care; ^c^The minimum includes compulsory questions without any follow-up questions that only applied to subgroups, the maximum includes all compulsory and optional questions

### Recruitment of participants

Recruitment of participants was undertaken digitally, through self-referral via a website. The website was promoted via PAO social channels, direct emails to organisation members and at meetings. Survey branding, a style guide, key messaging, a promotional animation clip, campaign flyers, proposed email content and social media materials were developed in collaboration with PAO members. A branded survey website was available (www.impactsurveyoi.com) for ongoing referral and information for potential participants.

Before and throughout fielding the project was presented at meetings of OI associations and through social media channels, while no other survey projects were promoted through these channels during that period. The OIFE provided regular updates to member organisations. To mitigate geographic and gender-based disparities in recruitment the steering committee reached out to the OIFE medical advisory board to medical professionals to mention the survey to their patients. This was effective in countries with less active PAOs or countries in which surveys are uncommon.

### Fielding

The survey was fielded online 1 July–30 September 2021.

### Analysis

Survey data were exported into Excel, translated back into English and compiled into a master database using the pandas Python software package. Free text responses were analysed and used to validate structured responses. Where possible, free text answers were compared with any structured responses in Microsoft Excel to identify mismatch or improve the accuracy of structured responses (e.g. where respondents had used the “Other” option, but the free text response matched one of the answer options). Additionally for all questions recurring themes in free text responses were identified in Microsoft Excel and free text containing recurring themes was quantified. Data were cleaned to exclude any outliers and non-sensical responses. Potential outliers (any values greater than 2 standard deviations from the median of continuous variables) were validated by clinicians. Data-cleaning and analysis were performed in Excel. The main features and characteristics of the data set were summarised with descriptive statistics. Unless otherwise specified “adults with OI” includes both, caregiver and non-caregiver adults with OI.

## Results

Overall, 2428 complete questionnaires were submitted. During the data cleaning stage 220 were excluded based on basic consistency checks of the responses leaving 2208 questionnaires for analysis, representing 2312 individuals with OI (1532 of whom answered questions about themselves, and 780 whose answers were provided by proxy) and 676 individuals without OI (560 of whom were caregivers without OI who answered questions about individuals with OI in their care, and 116 of whom were relatives of individuals with OI; Tables 1 and 2).

**Table 2:** Respondent and proxy respondent demographics

	Direct respondents					Proxy respondents		
	Non-caregiver adults with OI n = 1,290	Caregiver adults with OI n = 150	Adolescents with OI n = 92	Caregivers without OI n = 560	Relatives n = 116	Children with OI (n = 474)	Adolescents with OI (n = 171)	Adults with OI (n = 135)
Age, mean (range)^a^	43.4 (18–85)	42.0 (24–75)	14.8 (12–17)	41.9 (18–81)	43.3 (12–87)	5.8 (1–11)	14.0 (12–17)	25.6 (18–60)
Women, n (%)^b^	900 (70)	108 (72)	51 (55)	465 (83)	80 (69)	214 (45)	84 (49)	67 (50)
*Geography, n (%)*^c^								
Europe	807 (63)	104 (69)	41 (45)	319 (57)	102 (88)	258 (54)	111 (65)	92 (68)
North America	327 (25)	20 (13)	22 (24)	103 (18)	9 (8)	85 (18)	26 (15)	30 (22)
South America	55 (4)	6 (4)	8 (9)	33 (6)	2 (2)	32 (7)	3 (2)	5 (4)
Asia	62 (5)	16 (11)	20 (22)	87 (16)	1 (1)	84 (18)	21 (12)	7 (5)
Africa	6 (0.5)	1 (0.7)	0 (0)	5 (1)	1 (1)	3 (1)	2 (1)	1 (1)
Australia/Oceania	33 (3)	3 (2)	1 (1)	13 (2)	1 (1)	12 (3)	8 (5)	0 (0)

Osteogenesis imperfecta, OI

^a^Questions 29, 40 and 48 “What is your age?” and “What is your child/children’s age?”; ^b^;Questions 8, 303, 30, 41 and 49 “What is your sex?/What is the sex of your child/children? ^c^Questions 7, 302 and 370 “What is your country of residence?” It was assumed that proxy respondents reside in the same countries as their caregivers

### Individuals reporting their own experience (direct respondents)

Respondent demographics are summarised in Table 2 and Appendix Table 4. There were 1532 respondents with OI who provided information on their own experience including 1290 non-caregiver adults with OI (without care recipients) and 92 adolescents with OI. Additionally, 150 adults with OI who were also caregivers for individuals with OI (“caregivers with OI”) provided information on both their own experience and that of the individuals with OI in their care (see below).

Adults with OI (n = 1440, including non-caregiver adults with OI [n = 1290] and caregiver adults with OI [n = 150]) were mostly female, from Europe or North America, with a median age of 42 years (range 18–85). Among 92 adolescents with OI the median age was 15 years (range 12–18) and proportions of male and female respondents were similar. Most adolescents with OI were from Europe (45%).

Respondents without OI, comprising 560 caregivers without OI, and 116 close relatives of individuals with OI, reported on their experience of caring for or being related to a person with OI. Caregivers without OI had a median age of 42 years (range 18–81), were mostly female and from Europe (Table 2).

Of the 710 survey participants identifying as caregivers (560 without OI, 150 with OI), 700 provided valid data on care recipients with OI (proxy data on 10 care recipients with OI were excluded). Most caregivers (90%) provided care to 1 individual with OI to whom they were a parent (98%). Fewer caregivers cared for to 2 (8%) or 3 (2%) individuals with OI or had other relationships to the care recipient (3%, Appendix Table 4).

Close relatives responding to the survey were predominantly female (69%); they self-described as being partners (28%), siblings (20%), or friends (15%) of individuals with OI (Appendix Table 4).

### Care recipients with OI for whom caregivers provided survey answers by proxy (proxy respondents)

Of the 780 care recipients with OI who were proxy reported by 700 caregivers (with or without OI), most were children < 12 years (68%; median age 6 years, range 1–11), fewer were adolescents (24%; median age 14 years, range 12–17) or adults (19%; median age 26 years, range 18–60). Approximately half in each age group were female (Table 2).

### Clinical characteristics

#### Direct respondents

**Fig. 1:**
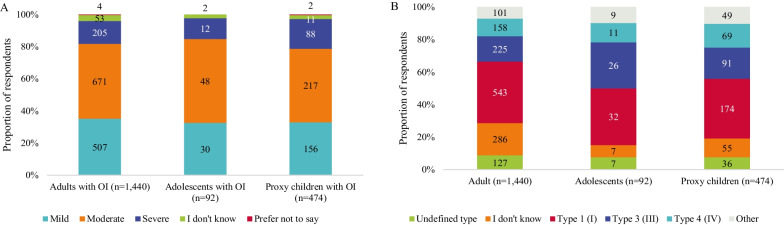
Respondents’ and proxy-respondents’(A) OI severity^a^ and (B) type^b^. OI, osteogenesis imperfecta. ^a^Questions 18, 32, 46, 54 and 312 “How would you describe the severity of your/your child’s/your children’s OI? Answer options included mild, moderate, severe, prefer not to say, I don’t know. ^b^Questions 17, 31, 45, 53 and 311.” If you/your child/your children have received an OI type as part of your OI diagnosis or treatment, please indicate your type using the dropdown below.” Answer options included Type 1–Type 15, undefined type, I don’t know, prefer not to say, other type. Participants who responded “other” could provide a free text answer

Most adults and adolescents with OI rated their OI as moderate (47% of adults and 52% of adolescents); the smallest proportion of individuals with OI rated their condition as severe (14% of adults and 13% of adolescents; Fig. 1A, Table 3). Similarly, most adults and adolescents with OI reported clinical OI type 1 (38% of adults and 35% of adolescents), 3 (16% of adults and 28% of adolescents) and 4 (11% of adults and 12% of adolescents; Fig. 1B and Table 3). Compared with adolescents, more adults were unaware of their clinical OI type (20% vs. 8% among adults and adolescents, respectively).

**Table 3:** OI-related clinical characteristics of individuals with OI

	Adults with OI (n = 1440)	Adolescents with OI (n = 92)	Proxy children with OI (n = 474)	Proxy adolescents with OI (n = 171)	Proxy adults with OI (n = 135)
*OI severity, n (%)*^a^					
Mild	507 (35)	30 (33)	157 (33)	63 (37)	38 (28)
Moderate	671 (47)	48 (52)	216 (46)	70 (41)	58 (43)
Severe	205 (14)	12 (13)	88 (19)	33 (19)	36 (27)
I don’t know	53 (4)	2 (2)	11 (2)	4 (2)	3 (2)
Prefer not to say	4 (0.3)	0 (0)	2 (0.4)	1 (0.6)	0 (0)
*OI type, n (%)*^b^					
Undefined type	127 (9)	7 (8)	36 (8)	19 (11)	7 (5)
I don’t know	286 (20)	7 (8)	55 (12)	24 (14)	25 (19)
Prefer not to say	2 (0.1)	0 (0)	1 (0.2)	0 (0)	2 (2)
Type 1 (I)	543 (38)	32 (35)	174 (37)	56 (33)	44 (33)
Type 2 (II)	23 (2)	2 (2)	7 (2)	3 (2)	3 (2)
Type 3 (III)	225 (16)	26 (28)	91 (19)	29 (17)	27 (20)
Type 4 (IV)	158 (11)	11 (12)	69 (15)	27 (16)	21 (16)
Type 5 (V)	26 (2)	3 (3)	16 (3)	4 (2)	1 (0.7)
Type 6 (VI)	4 (0.3)	0 (0)	8 (2)	2 (1)	0 (0)
Type 7 (VII)	2 (0.1)	0 (0)	1 (0.2)	0 (0)	0 (0)
Type 8 (VIII)	1 (0.1)	0 (0)	1 (0.2)	3 (2)	0 (0)
Type 9 (IX)	1 (0.1)	1 (1)	0 (0)	0 (0)	0 (0)
Type 11 (XI)	2 (0.1)	1 (1)	0 (0)	0 (0)	0 (0)
Type 14 (XIV)	0 (0)	0 (0)	0 (0)	0 (0)	1 (1)
Type 15 (XV)	1 (0.1)	0 (0)	5 (1)	0 (0)	0 (0)
Other	39 (3)	2 (2)	10 (2)	4 (2)	4 (3)
*Genetic confirmation, n (%)*^c^					
Yes	916 (64)	73 (79)	397 (84)	130 (76)	95 (70)
No	370 (26)	12 (13)	63 (14)	34 (20)	34 (25)
I don’t know	153 (11)	6 (7)	14 (3)	7 (4)	5 (4)
Prefer not to say	1 (0.1)	1 (1)	0 (0)	0 (0)	1 (0.7)
*Mobility, n (%)*^d^					
Walking (inside)	900 (63)	56 (61)	338 (71)	118 (69)	82 (61)
Walking (outside)	730 (51)	49 (53)	300 (63)	112 (66)	80 (59)
Cane/walking stick (inside)	71 (5)	3 (3)	6 (1)	6 (4)	3 (2)
Cane/walking stick (outside)	163 (11)	4 (4)	6 (1)	7 (4)	5 (4)
Walking frame (inside)	20 (1)	6 (7)	23 (5)	15 (9)	1 (0.7)
Walking frame (outside)	16 (1)	3 (3)	15 (3)	6 (4)	0 (0)
Rollator (inside)	55 (4)	7 (8)	23 (5)	8 (5)	2 (1)
Rollator (outside)	61 (4)	2 (2)	15 (3)	6 (4)	4 (3)
Crutches (inside)	96 (7)	6 (7)	9 (2)	6 (4)	7 (5)
Crutches (outside)	136 (9)	9 (10)	3 (0.6)	4 (2)	7 (5)
Manuel wheelchair (inside)	300 (21)	18 (20)	61 (13)	38 (22)	27 (20)
Manuel wheelchair (outside)	370 (26)	33 (36)	91 (19)	61 (36)	33 (24)
Powered wheelchair (inside)	125 (9)	5 (5)	9 (2)	3 (2)	12 (9)
Powered wheelchair (outside)	198 (14)	8 (9)	16 (3)	10 (6)	18 (13)
Mobility scooter (inside)	4 (0.3)	0 (0)	6 (1)	0 (0)	0 (0)
Mobility scooter (outside)	65 (5)	0 (0)	3 (0.6)	1 (0.6)	2 (1)
Crawling (inside)	58 (4)	9 (10)	115 (24)	25 (15)	3 (2)
Crawling (outside)	13 (0.9)	0 (0)	46 (10)	2 (1)	0 (0)
Being carried (inside)	26 (2)	8 (9)	130 (27)	14 (8)	5 (4)
Being carried (outside)	32 (2)	10 (11)	136 (29)	17 (10)	2 (1)
Laying in bed or stretcher (inside)	20 (1)	7 (8)	36 (8)	4 (2)	3 (2)
Laying in bed or stretcher (outside)	4 (0.3)	0 (0)	15 (3)	3 (2)	2 (1)
Other (inside)	29 (2)	5 (5)	24 (5)	2 (1)	0 (0)
Other (outside)	35 (2)	3 (3)	28 (6)	4 (2)	2 (1)
*Height, n (%)*^e^					
< 50 cm	9 (0.6)	1 (1)	7 (1)	1 (0.6)	0 (0)
≥ 50–80 cm	13 (0.9)	1 (1)	71 (15)	2 (1)	4 (3)
> 80–100 cm	95 (7)	4 (4)	126 (27)	11 (6)	9 (7)
> 100–130 cm	270 (19)	23 (25)	179 (38)	27 (16)	21 (16)
> 130–160 cm	702 (49)	44 (48)	54 (11)	86 (50)	49 (36)
> 160 cm	331 (23)	13 (14)	0 (0)	40 (23)	48 (36)
I don’t know	18 (1)	5 (5)	36 (8)	4 (2)	3 (2)
Prefer not to say	2 (0.1)	1 (1)	1 (0.2)	0 (0)	1 (0.7)

OI, osteogenesis imperfecta

^a^Questions 18, 32, 46, 54 and 312 “How would you describe the severity of your/your child’s/your children’s OI? Answer options included mild, moderate, severe, prefer not to say, I don’t know. ^b^Questions 17, 31, 45, 53 and 311.” If you/your child/your children have received an OI type as part of your OI diagnosis or treatment, please indicate your type using the dropdown below.” Answer options included Type 1–Type 15, undefined type, I don’t know, prefer not to say, other type. Participants who responded “other” could provide a free text answer. ^c^Questions 19, 33, 47, 55 “Do you/your child/your children have a genetically confirmed diagnosis of OI”. Answer options included yes, I don’t know, prefer not to say. ^d^Questions 16, 36, 66, 67, 310 “How do you/your child/your children get around?”. Answer options for both outside and inside your home included Walking unaided, cane/walking stick, walking frame, rollator (wheeled walker), crutches, manual wheelchair, powered wheelchair, mobility scooter, crawling, being carried, laying in bed/stretcher, other (please specify below). Respondents could choose more than one answer in each category. ^e^Questions 14, 15, 27, 28, 43, 44, 51, 52, 308, 309 “What is your/your child’s height”. Answer options included metric or imperial measurements and “I don’t know”, “Prefer to say”. All values were converted to metric measurements

Most adults and adolescents with OI had a genetic confirmation of their diagnosis. Data on height provided further context to individuals’ OI type—most adults were 130–160 cm tall (49%), fewer were 100–130 cm tall (19%). This distribution was similar among adolescents (Table 3).

Most adults and adolescents reported walking unaided both inside and outside. Manual wheelchair use was the second most common mode of mobility (up to 36% of respondents), power chairs in contrast were used by only up to 14% of respondents (Table 3).

**Fig. 2:**
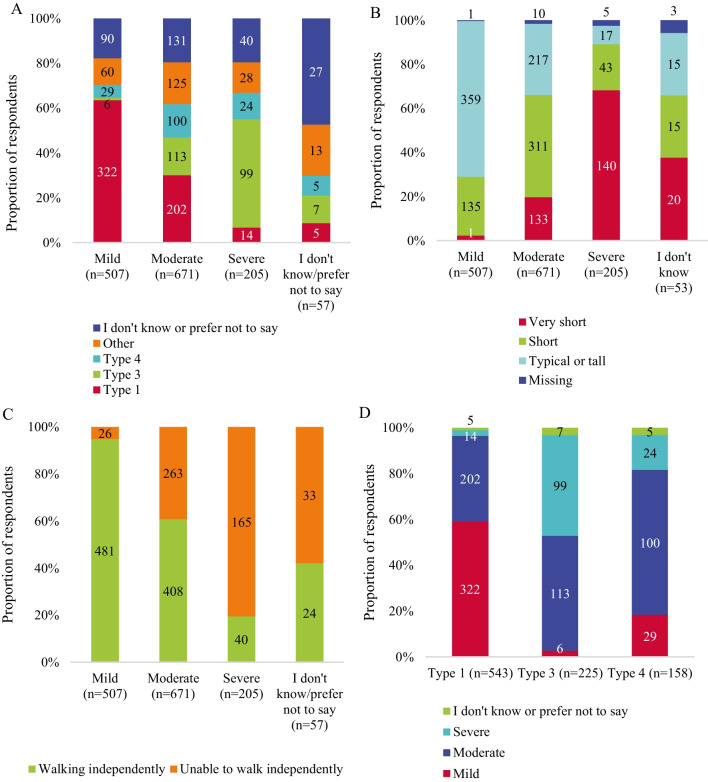
Alignment of OI severity and type (**A**), severity and height (**B**)^a^, and severity and mobility (**C**)^b^ OI type and severity (**D**) among adults with OI (n = 1440). OI, osteogenesis imperfecta. ^a^Typical or tall includes women > 150 cm and men > 160 cm height; short includes women 120–150 cm and men 130–160 cm. Women and men < 120 and < 130 cm respectively were grouped in the very short category. Individuals who preferred not to provide their gender were assessed in the female category. ^b^Individuals who reported to be walking without assistance in- or outside were considered to be walking independetly. All others were considered to be unable to walk independently

Most adults with mild OI reported OI type 1 (64%), ability to walk independently in- or outside (95%) and typical or tall height (71%; includes women ≥ 150 cm and men ≥ 160 cm). Adults reporting moderate OI were split across clinical OI types: most reported OI type 1 (30%) and similar proportions reported types 3 (17%) and 4 (15%). Adults with moderate OI predominantly reported short height (46%; includes women 120–150 cm and men 130–160 cm) and ability to walk independently (61%). Conversely, adults with severe OI predominantly reported OI type 3 (48%), inability to walk independently (81%) and very short height (68%; women < 120 and men < 130 cm; Fig. 2, Appendix Tables 5, 6, 7, 8).

When considering the relationship between self-reported clinical type and OI severity, adults with OI type 1 commonly reported mild (59%) and moderate (37%) OI severity. Those with OI type 3 were split across moderate (50%) and severe (44%) OI severity. Adults with OI type 4 predominantly reported moderate OI (63%), however mild (18%) and severe OI (15%) were also reported (Fig. 2, Appendix Tables 5, 6, 7, 8).

#### Proxy respondents

The OI of most proxy respondents was described as moderate by their caregivers (46% of proxy children, 41% of proxy adolescents and 43% of proxy adults; Table 3). Severe OI was least frequently reported among proxy children (19%) and proxy adolescents (19%). However, mild (28%) and severe OI (27%) were reported in similar proportions in proxy adults. Proportions of OI types 1, 3 and 4 were similar across all proxy-reported age groups (Fig. 1B and Table 3). Awareness of clinical OI type and proportion of genetically confirmed diagnoses decreased slightly with increasing proxy-respondent age.

Most children walked inside (71%) and outside (63%), were carried inside (27%) and outside (29%), or used a manual wheelchair inside (13%) and outside (19%). Proportions of proxy adolescents and adults who walked independently (and used a manual wheelchair were distributed similarly to direct respondents. Most children were 100–130 cm tall (38%); For proxy adolescents a height of 130–160 cm was most commonly reported (50%). Equal proportions of proxy adults were 130–160 cm and over 160 cm tall (both 36%; Table 3).

#### Signs, symptoms and events in the past 12 months

The survey explored the prevalence of clinical signs, symptoms and events in the past 12 months and throughout an individuals’ lifetime. Here, events reported in the past 12 months are explored.

#### Direct respondents

**Fig. 3:**
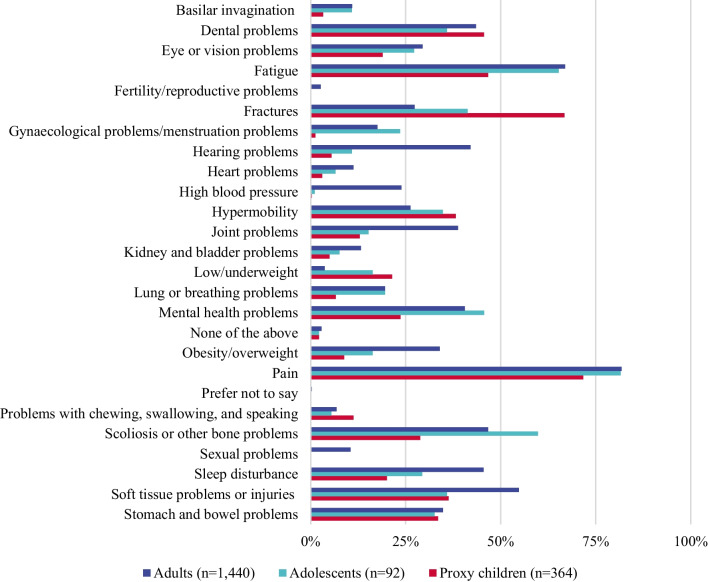
Prevalence of clinical events, signs and symptoms in participants with OI^a^. OI, osteogenesis imperfecta. ^a^Questions 113, 187, 251–253 “Over the past 12 months, have you/your child experienced any of the following signs, symptoms or events? Answer options included 24 options (see above) for adults and 22 options for care recipients and adolescents (excluding sexual health and fertility) and “I don’t know”, “prefer not to say”

Pain was the most widely reported condition experienced in the past 12 months in adults (82%) and adolescents (82%); Fig. 3A and Appendix Table 9). Other commonly reported signs, symptoms and events among adults included: fatigue (67%), scoliosis and other bone problems (47%), sleep disturbance (45%), dental problems (43%), hearing problems (42%), mental health problems (41%). In adolescents, in addition to pain, fatigue (65%), scoliosis and other bone problems (60%), mental health problems (46%) and fractures (41%) were common.

#### Proxy respondents

In proxy children pain (72%), fractures (67%), fatigue (47%) and dental problems (46%; Fig. 3) were commonly reported. For proxy adolescents such signs, symptoms and events included, pain (79%), scoliosis and other bone problems (60%), fatigue (54%), fractures (54%), and soft tissue injuries (52%; Appendix Table 9); among proxy adults pain (76%) and fatigue (49%) were most common (Appendix Table 9).

#### Impact of signs, symptoms and events experienced in the past 12 months

Respondents who reported that they, or individuals with OI in their care, experienced signs, symptoms and events associated with OI were asked follow-up questions on how they were affected by their signs, symptoms and events.

#### Direct respondents

**Fig. 4:**
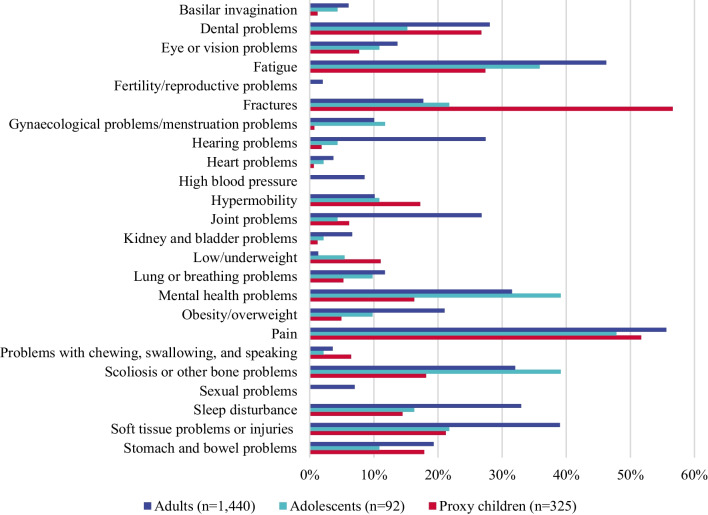
Moderate-severe impact of clinical events, signs and symptoms in adults, adolescents and children with OI^a^. OI, osteogenesis imperfecta. ^a^Questions 114, 116, 118–131, 133–142, 188, 191, 193, 194, 195, 197–215, 319, 321, 323, 324, 325, 327–345 “In the past 12 months, how has x impacted your life”, where x is any clinical sign, symptom or event participants reported to have experienced in the past 12 month in questions 113, 187, 251–253. These follow up questions were unavailable for caregivers who provided information on multiple care recipients. Respondents chose a single answer option from a 5-level Likert scale “Not at all impacted–Severely impacted” and I don’t know, prefer not to say. The prevalence of gynaecological problems was calculated within the female subpopulations (n = 1008, n = 51 and n = 137, respectively)

Pain, fatigue and scoliosis were most commonly reported as moderately-severely impactful by adults and adolescents (56–36% reporting moderate-severe impact; Fig. 4, Appendix Tables 10 and 11). Additionally, adults commonly rated soft tissue (39%) and sleep problems (33%) as moderately-severely impactful, while adolescents most commonly rated mental health problems (39%) and fractures (22%) as moderately-severely impactful signs, symptoms and events.

#### Proxy respondents

Pain (52%), fractures (57%), dental problems (27%), fatigue (27%) and soft tissue problems (21%) were most commonly rated as moderately-severely impactful in children (Fig. 4, Appendix Table 12). Among proxy adolescents such signs, symptoms and events included pain (79%), scoliosis and other bone problems (60%), fractures (54%), fatigue (54%) and soft tissue problems (52%; Appendix Table 13). For proxy adults, most commonly moderately-severely impactful signs, symptoms and events included pain (76%), fatigue (49%), scoliosis and other bone problems (40%), dental problems (37%), sleep disturbance (32%) and mental health problems (33%; Appendix Table 14).

## Discussion

The IMPACT Survey has compiled the largest known patient-reported dataset on the experience of individuals with OI (n = 2312 individuals across self- and proxy reports), and their caregivers (n = 560) to date. This was achieved through an ambitious and self-driven collaboration between the clinical and OI communities, aided by third-party funding from a pharmaceutical manufacturer. Past research on the impact of OI on individuals and their caregivers has been limited by small sample sizes and did not cover the breadth of topics included in IMPACT [6, 7, 9, 18, 19]. Therefore, IMPACT was designed as an online survey to maximise accessibility across geographies and included questions covering a breadth of topics exploring the clinical, humanistic, and economic impact of OI that may be insufficiently documented in the existing literature.

While in this survey sample self-reported OI severity correlated well with other characteristics like height, mobility and clinical OI type, OI types were split across multiple OI severities. This finding mirrors previous research and suggests that “severity” is a holistic, patient-centric view of individuals’ health, that is not entirely captured by the clinical OI type which is determined based on clinical and molecular patient characteristics [20, 21]. Additionally, while general predictions about the clinical OI type can be made based on the causative gene, mutation type and location, genetic variation alone does not account for all phenotypic variation [22–24].

Some characteristics of this sample reflect the nature of the study. While OI is equally prevalent in men and women, most direct respondents to the survey were female. This is in line with past patient-reported outcomes research in OI, where an increased participation of women with OI and female caregivers is common [9]. Most direct respondents were from Europe and European respondents made up > 50% of participants. This strong representation of European individuals is unsurprising as survey languages and recruitment efforts focussed on this region, although the survey was open to participants from any geography. OI type 1, which is commonly associated with mild OI, was the most commonly reported type across direct and proxy respondents. This is a somewhat lower prevalence of type 1 than reported in past population-based studies, but in line with other large surveys among individuals with OI [1, 20, 25]. Past research has shown that in samples selected primarily based on individuals’ availability and willingness to participate, more engaged individuals (e.g., those who participate more in a computer game or are more involved with an organisation) who may also be more severely affected by a given health concern are overrepresented [26, 27]. Moreso, past patient-reported outcomes research similarly underrepresented individuals with OI type 1 [9, 28].

The survey includes adults (both self- and proxy reported), adolescents (self- and proxy reported), caregivers (with and without OI) of individuals with OI, children (proxy-reported), and relatives of individuals with OI. Caregivers also provided information on the OI-related QoL of any siblings to individuals with OI in their care. For some of these populations few data are available elsewhere: comparatively few studies, which commonly have small samples, provide information on the wellbeing of caregivers and siblings [29, 30] and few studies include self-reporting adolescents [9].

This study found that, irrespective of age, individuals with OI experience numerous signs, symptoms and events that affect their HRQoL. Pain was highly prevalent and impactful on the HRQoL of individuals with OI, as reported in past research [20, 31–33]. Notably, fatigue, which has rarely been explored in children, was prevalent in children in this sample, but much less so than adults or adolescents. Past research also found that fatigue was prevalent in adults but not children [20, 34]. Furthermore, our data suggest that fatigue may be underreported in caregiver-based studies: in our dataset, fatigue was reported by much higher proportions of self-reporting compared with proxy-reported individuals. This discrepancy has also been detected for numerous other clinical events, signs and symptoms including, in adolescents compared with proxy adolescents, gynaecological and menstruation problems, mental health problems, sleep disturbance and stomach and bowel problems. Similarly, differences were also common among reports of adults compared with proxy adults, for example in the proportion of individuals experiencing hearing problems, fatigue, sleep disturbance, stomach and bowel problems and mental health problems. While the survey does not include self-reported paediatric data, this suggests that differences in self- and proxy report may be an issue in the paediatric population as well. Past literature largely reports that parents underreport pain and some other not directly observable health conditions, however some reports found relatively high agreement between self- and proxy report [35–42]. Nonetheless, underreporting may contribute to suboptimal health outcomes due to lack of attention focused on understanding a health issue; while there are few reports on bowel problems in the OI population, past research lists diseases of the digestive system as a common cause of death [43, 44]. Among adults and adolescents, our survey has found a high prevalence of bowel and stomach issues.

Our survey has also detected a high prevalence of mental health problems in adults, adolescents and, to a lesser extent, children. Past literature on the mental health impact of OI is conflicting: while some reports found higher prevalence of mental health issues in adults and children, others have reported that the mental health of individuals with OI is preserved which in part may be due to the adaptation process experienced by individuals with chronic conditions [8, 19, 20, 31, 34, 45–47]. More detailed exploration of the mental health impact of OI and affected areas of life will be the subject of additional analyses of the IMPACT dataset.

A mental health impact of OI may be aligned with participants’ responses on the effect of signs, symptoms and events on their life. Pain and fatigue were commonly perceived as negatively impactful across survey populations and these conditions have indeed been associated with a negative impact on mental health in past reports [48–50]. Additionally, the negative impact of OI may increase with age: larger proportions of adults described signs, symptoms and events they experienced as moderately-severely impactful compared with adolescents. This may be due to an overall increasing burden of health events and/or a progressively decreasing resilience to health events.

Similarly sleep disturbance was commonly reported among all age groups. This finding is aligned with past literature [20, 34, 51, 52] and may be correlated with the findings of a high prevalence of mental health issues and fatigue in the OI population [53–55]

It remains to be explored whether the changes in prevalence and impact of clinical symptoms are caused by underlying differences in respondent demographics, differences in awareness and perception of symptoms by individuals with OI and their caregivers, or changes in symptom intensity or resilience.

Further analysis of the survey responses may fill current data gaps in our understanding of the lived experience of the OI community, to improve treatment and care, and to evaluate potential differences across diverse geographical or cultural settings.

### Strengths and limitations

The IMPACT survey was developed in collaboration with researchers and OI community members to ensure that the survey design is relevant and inclusive. To this end, questions were adjusted to fit multiple demographics, including individuals who are underrepresented in existing research. The design was also adapted to cover a wide range of geographies, include relevant and culturally appropriate answer options, and was translated into 8 languages to include as many members of the community as possible. However, though IMPACT did not include any validated patient-reported outcome tools, questions were validated by members of the OI community and academic researchers. Our study does not include a control population which limits the generalisability of our findings; comparison with past population-based research can provide insights into the significance of our findings but may not be able to fully mitigate this shortcoming. Questions about ethnicity were omitted due to the breadth of potential fielding geographies and the resulting multitude of possible responses and respondent attitudes on questions about this topic.

During its 3-month fielding period the survey generated a large sample that includes a wide range of community members including demographics for which few data are available, e.g., adolescents and caregivers (with or without OI) of multiple individuals with OI. Despite our best efforts to recruit men, most survey respondents were female. As the survey includes a convenience sample of individuals recruited by patient organisations, the survey population includes those who were able to respond to a survey online and are engaged with PAOs.

Lastly, any self-reported data are limited by the respondents’ accuracy and recall bias. As the survey was fielded in 2021 under the extraordinary circumstances of the Coronavirus Disease Pandemic 2019 (COVID-19) pandemic in 2021 this bias may be exacerbated, and the generalisability of some survey outcomes may be affected. To mitigate this limitation and contextualise survey findings, the survey included questions about behavioural changes among individuals with OI and caregivers due to the COVID-19 pandemic.

## Conclusion

IMPACT has generated an extensive dataset on the experience of individuals with OI, their caregivers and relatives. Upcoming analyses of the IMPACT data will further our understanding of the humanistic, economic and clinical burden of OI and contribute to improved treatment and care for the OI community.

### Supplementary Information

**Additional file 1**. IMPACT Survey Questionnaire.

## Data Availability

The data that support the findings of this study are not openly available due to reasons of sensitivity. They are managed by a data management committee and are available upon reasonable request to the authors.

## Appendix

See Table 4, 5, 6, 7, 8, 9, 10, 11, 12, 13 and 14.

**Table 4:** Caregiver and close relative demographics

**Caregiver relation, n (%)**^**b****,****c**^	**n = 700**^**a**^
Parent	684 (98)
Sibling	2 (0.3)
Grandparent	7 (1)
Legal guardian	18 (3)
Prefer not to say	0 (0)
Other	2 (0.3)
Number of care recipients with OI in household, n (%)^d,c^	
Households with 1 care recipient	632 (90)
Households with 2 care recipients	56 (8)
Households with 3 care recipients	12 (2)
Number of care recipients without OI in household, n (%)^d^	
Households with 0 siblings	302 (43)
Households with 1 sibling	284 (41)
Households with 2 siblings	91 (13)
Households with 3 siblings	18 (3)
Households with 4 siblings	5 (0.7)
**Close relative relation, n (%)**^**e**^	**n=116**
Partner of a person with OI	33 (28)
Sibling of a person with OI	23 (20)
Friend of a person with OI	17 (15)
Grandparent of a person with OI	14 (12)
Aunt of a person with OI	8 (7)
Child of a person with OI	4 (3)
Friend of a caregiver for a child with OI	3 (3)
Support teacher of a person with OI	3 (3)
Doctor of a person with OI	2 (2)
Grandchild of a person with OI	2 (2)
Great aunt of a person with OI	2 (2)
Brother-in-law of a person with OI	1 (1)
Caregiver of a person with OI	1 (1)
Godmother	1 (1)
Mother of a person with OI	1 (1)
Mother of a deceased child with OI	1 (1)

OI, osteogenesis imperfecta

^a^Includes caregiver adults with OI and caregivers without OI. Of 710 caregivers 700 provided information on care recipients with OI; ^b^14 respondents indicated > 1 relation to a care recipient; ^c^Question 24 “What is your relationship to the child/children with OI in your care?”. Respondents could pick more than one option including: parent, sibling, grandparent, legal guardian, prefer not to say, other and provide free text. ^d^Questions 22 and 23 included a drop down with numerals from 1 to 3 and 0 to 4 respectively. ^e^Question 396 “Please indicate which of the following best describes your relationship to the person with OI”. Answer options included: child of a person with OI, sibling of a person with OI, grandparent of a person with OI, partner of a person with OI, other relationship to a person with OI (please specify below)

**Table 5:** Association of OI severity and type among adults with OI (n = 1440)

n (%)	Type 1 (n = 543)	Type 3 (n = 225)	Type 4 (n = 158)	Other (n = 226)	I don’t know or prefer not to say (n = 288)
Mild (n = 507)	322 (64)	6 (1)	29 (6)	60 (12)	90 (18)
Moderate (n = 671)	202 (30)	113 (17)	100 (15)	125 (19)	131 (20)
Severe (n = 205)	14 (7)	99 (48)	24 (12)	28 (14)	40 (20)
I don’t know/prefer not to say (n = 57)	5 (9)	7 (12)	5 (9)	13 (23)	27 (47)

**Table 6:** Association of OI severity and height among adults with OI (n = 1,440)

n (%)	Very short (n = 305)	Short (n = 507)	Typical or tall (n = 608)	I don’t know/prefer not to say (n = 20)
Mild (n = 507)	12 (2)	135 (27)	359 (71)	1 (0.2)
Moderate (n = 671)	133 (20)	311 (46)	217 (32)	10 (1)
Severe (n = 205)	140 (68)	43 (21)	17 (8)	5 (2)
I don’t know/prefer not to say (n = 57)	20 (35)	18 (32)	15 (26)	4 (7)

**Table 7:** Association of OI severity and walking status among adults with OI (n = 1440)

n (%)	Unable to walk independently in-or outside (n = 487)	Walking independently in- or outside (n = 953)
Mild (n = 507)	26 (5)	481 (95)
Moderate (n = 671)	263 (39)	408 (61)
Severe (n = 205)	165 (80)	40 (20)
I don’t know/prefer not to say (n = 57)	33 (58)	24 (42)

**Table 8:** Association of OI type and severity among adults with OI (n = 1440)

n (%)	Mild	Moderate	Severe	I don’t know or prefer not to say
Type 1 (n = 543)	322 (59)	202 (37)	14 (3)	5 (0.9)
Type 3 (n = 225)	6 (3)	113 (50)	99 (44)	7 (3)
Type 4 (n = 158)	29 (18)	100 (63)	24 (15)	5 (3)

**Table 9:** Prevalence of clinical signs, symptoms and events^a^

n, (%)	Adults (n = 1,440)	Adolescents (n = 92)	Proxy children (n = 364)	Proxy adolescents (n = 136)	Proxy adults (n = 95)
Basilar invagination	157 (11)	10 (11)	12 (3)	9 (6)	4 (4)
Dental problems	626 (43)	33 (36)	166 (46)	52 (38)	35 (37)
Eye or vision problems	424 (29)	25 (27)	69 (19)	21 (15)	16 (17)
Fatigue	964 (67)	60 (65)	170 (47)	74 (54)	47 (49)
Fertility and reproductive problems	38 (3)	NA	NA	NA	NA
Fractures	394 (27)	38 (41)	243 (67)	74 (54)	26 (27)
Gynaecological and menstruation problems^b^	174 (17)	12 (24)	2 (0.9)	7 (11)	7 (17)
Hearing problems	606 (42)	10 (11)	20 (5)	9 (7)	12 (13)
Heart problems	162 (11)	6 (7)	11 (3)	3 (2)	1 (1)
High blood pressure	344 (24)	1 (1)	1 (0.2)	0 (0)	7 (7)
Hypermobility	378 (26)	32 (35)	139 (38)	50 (37)	21 (22)
Joint problems	558 (39)	14 (15)	47 (13)	31 (23)	15 (16)
Kidney and bladder problems	190 (13)	7 (8)	18 (5)	6 (4)	4 (4)
Low and underweight	53 (4)	15 (16)	78 (21)	16 (12)	8 (8)
Lung or breathing problems	282 (20)	18 (20)	24 (7)	11 (8)	12 (13)
Mental health problems	584 (41)	42 (46)	86 (24)	37 (27)	31 (33)
Obesity and overweight	489 (34)	15 (16)	32 (9)	22 (16)	24 (25)
Pain	1,178 (82)	75 (82)	261 (72)	108 (79)	72 (76)
Problems with chewing, swallowing, and speaking	98 (7)	5 (5)	41 (11)	4 (3)	11 (12)
Scoliosis or other bone problems	672 (47)	55 (60)	105 (29)	82 (60)	38 (40)
Sexual problems	151 (11)	NA	NA	NA	NA
Sleep disturbance	655 (46)	27 (29)	73 (20)	29 (21)	30 (32)
Soft tissue problems or injuries	789 (55)	33 (36)	132 (36)	71 (52)	37 (39)
Stomach and bowel problems	501 (35)	30 (33)	122 (34)	34 (25)	21 (22)
None of the above	41 (3)	2 (2)	8 (2)	3 (2)	5 (5)
Prefer not to say	4 (0.3)	0 (0)	1 (0.2)	0 (0)	2 (2)

^a^Questions 113, 187, 251–253 “Over the past 12 months, have you/your child experienced any of the following signs, symptoms or events? Answer options included 24 options (see above) for adults and 22 options for care recipients and adolescents (excluding sexual health and fertility) and “I don’t know”, “prefer not to say” ^b^ Calculated as proportion of women and girls responding to the survey

NA, not available

**Table 10:** Impact of signs, symptoms and events in adults with OI (n = 1,440)^a^

n, (%)	n affected^b^	Severely impacted	Moderately impacted	Mildly impacted	Very mildly impacted	Not impacted	I don’t know	Prefer not to say	Missing
Basilar invagination	157	27 (2)	60 (4)	48 (3)	16 (1)	3 (0.2)	2 (0.1)	0 (0)	1 (0.1)
Dental problems	626	166 (12)	238 (17)	151 (11)	51 (4)	15 (1)	1 (0.1)	1 (0.1)	3 (0.2)
Eye or vision problems	424	55 (4)	142 (10)	155 (11)	58 (4)	9 (0.6)	2 (0.1)	0 (0)	3 (0.2)
Fatigue	964	255 (18)	411 (29)	216 (15)	69 (5)	6 (0.4)	3 (0.2)	1 (0.1)	3 (0.2)
Fertility and reproductive problems	38	18 (1)	11 (0.8)	2 (0.1)	2 (0.1)	5 (0.3)	0 (0)	0 (0)	0 (0)
Fractures	394	103 (7)	152 (11)	86 (6)	36 (3)	11 (0.8)	3 (0.2)	1 (0.1)	2 (0.1)
Gynaecological and menstruation problem	174	33 (3)	68 (7)	49 (5)	16 (2)	5 (0.5)	1 (0.1)	2 (0.2)	0 (0)
Hearing problems	606	163 (11)	232 (16)	143 (10)	52 (4)	12 (0.8)	1 (0.1)	0 (0)	3 (0.2)
Heart problems	162	16 (1)	37 (3)	45 (3)	34 (2)	26 (2)	4 (0.3)	0 (0)	0 (0)
High blood pressure	344	27 (2)	96 (7)	118 (8)	61 (4)	39 (3)	0 (0)	1 (0.1)	2 (0.1)
Hypermobility	378	37 (3)	109 (8)	104 (7)	69 (5)	54 (4)	5 (0.3)	0 (0)	0 (0)
Joint problems	558	150 (10)	236 (16)	103 (7)	47 (3)	16 (1)	4 (0.3)	0 (0)	2 (0.1)
Kidney and bladder problems	190	24 (2)	71 (5)	57 (4)	27 (2)	8 (0.6)	1 (0.1)	0 (0)	2 (0.1)
Low/underweight	53	7 (0.5)	12 (0.8)	16 (1)	11 (0.8)	5 (0.4)	2 (0.1)	0 (0)	0 (0)
Lung or breathing problems	282	56 (4)	113 (8)	77 (5)	30 (2)	5 (0.3)	0 (0)	0 (0)	1 (0.1)
Mental health problems	584	187 (13)	267 (19)	110 (8)	19 (1)	0 (0)	1 (0.1)	0 (0)	0 (0)
Obesity/overweight	489	112 (8)	191 (13)	120 (8)	48 (3)	17 (1)	1 (0.1)	0 (0)	0 (0)
Pain	1,178	280 (19)	521 (36)	265 (18)	97 (7)	7 (0.5)	1 (0.1)	2 (0.1)	5 (0.3)
Problems with chewing, swallowing, and speaking	98	22 (2)	30 (2)	28 (2)	16 (1)	1 (0.1)	0 (0)	0 (0)	1 (0.1)
Scoliosis or other bone problems	672	158 (11)	303 (21)	147 (10)	46 (3)	10 (0.7)	6 (0.4)	0 (0)	2 (0.1)
Sexual problems	151	48 (3)	53 (4)	34 (2)	9 (0.6)	1 (0.1)	3 (0.2)	3 (0.2)	0 (0)
Sleep disturbance	655	174 (12)	301 (21)	143 (10)	29 (2)	4 (0.3)	1 (0.1)	1 (0.1)	2 (0.1)
Soft tissue problems or injuries	789	181 (13)	381 (27)	154 (11)	56 (4)	12 (0.8)	2 (0.1)	0 (0)	3 (0.2)
Stomach and bowel problems	501	81 (6)	197 (14)	150 (10)	59 (4)	11 (0.8)	0 (0)	1 (0.1)	2 (0.1)

OI, osteogenesis imperfecta

^a^Questions 114, 116, 118–131 and 133–142 “In the past 12 months, how has x impacted your life”, where x is any clinical sign, symptom or event participants reported to have experienced in the past 12 month in questions 113. Respondents chose a single answer option from a 5-level Likert scale “Not at all impacted–Severely impacted” and I don’t know, prefer not to say. ^b^Based on responses to question 113. Percentages of affected individuals were calculated for the overall population of 1,440 adults for all but gynaecological problems, where percentages were calculated based on the number of women (n = 1,008) in the survey

**Table 11:** Impact of signs, symptoms and events in adolescents (n = 92)^a^

n, (%)	n affected^b^	Severely impacted	Moderately impacted	Mildly impacted	Very mildly impacted	Not impacted	I don’t know	Prefer not to say	Missing
Basilar invagination	10	0 (0)	4 (4)	2 (2)	4 (4)	0 (0)	0 (0)	0 (0)	0 (0)
Dental problems	33	3 (3)	11 (12)	12 (13)	4 (4)	2 (2)	0 (0)	0 (0)	1 (1)
Eye or vision problems	25	2 (2)	8 (9)	8 (9)	5 (5)	1 (1)	0 (0)	0 (0)	1 (1)
Fatigue	60	9 (10)	24 (26)	18 (20)	6 (7)	1 (1)	1 (1)	0 (0)	1 (1)
Fractures	38	6 (7)	14 (15)	13 (14)	3 (3)	0 (0)	1 (1)	0 (0)	1 (1)
Gynaecological and menstruation problems	12	1 (2)	5 (10)	3 (6)	1 (2)	2 (4)	0 (0)	0 (0)	0 (0)
Hearing problems	10	0 (0)	4 (4)	2 (2)	2 (2)	1 (1)	0 (0)	0 (0)	1 (1)
Heart problems	6	0 (0)	2 (2)	3 (3)	0 (0)	1 (1)	0 (0)	0 (0)	0 (0)
High blood pressure	1	0 (0)	0 (0)	0 (0)	0 (0)	1 (1)	0 (0)	0 (0)	0 (0)
Hypermobility	32	0 (0)	10 (11)	12 (13)	6 (7)	4 (4)	0 (0)	0 (0)	0 (0)
Joint problems	14	1 (1)	3 (3)	7 (8)	1 (1)	2 (2)	0 (0)	0 (0)	0 (0)
Kidney and bladder problems	7	1 (1)	1 (1)	1 (1)	2 (2)	2 (2)	0 (0)	0 (0)	0 (0)
Low and underweight	15	3 (3)	2 (2)	1 (1)	4 (4)	5 (5)	0 (0)	0 (0)	0 (0)
Lung or breathing problems	18	3 (3)	6 (7)	6 (7)	3 (3)	0 (0)	0 (0)	0 (0)	0 (0)
Mental health problems	42	18 (20)	18 (20)	4 (4)	2 (2)	0 (0)	0 (0)	0 (0)	0 (0)
Obesity and overweight	15	6 (7)	3 (3)	6 (6)	0 (0)	0 (0)	0 (0)	0 (0)	0 (0)
Pain	75	10 (11)	34 (37)	21 (23)	8 (9)	1 (1)	0 (0)	0 (0)	1 (1)
Problems with chewing, swallowing, and speaking	5	0 (0)	2 (2)	2 (2)	0 (0)	1 (1)	0 (0)	0 (0)	0 (0)
Scoliosis or other bone problems	55	13 (14)	23 (25)	9 (10)	4 (4)	5 (5)	0 (0)	0 (0)	1 (1)
Sleep disturbance	27	6 (7)	9 (10)	6 (7)	3 (3)	2 (2)	0 (0)	0 (0)	1 (1)
Soft tissue problems or injuries	33	6 (7)	14 (15)	11 (12)	2 (2)	1 (1)	0 (0)	0 (0)	0 (0)
Stomach and bowel problems	30	3 (3)	7 (8)	10 (11)	8 (9)	1 (1)	0 (0)	0 (0)	1 (1)

OI, osteogenesis imperfecta

^a^Questions 319, 321, 323, 324, 325, 327–345 “In the past 12 months, how has x impacted your life”, where x is any clinical sign, symptom or event participants reported to have experienced in the past 12 month in questions 318. Respondents chose a single answer option from a 5-level Likert scale “Not at all impacted–Severely impacted” and I don’t know, prefer not to say. ^b^ Based on responses to question 318. Percentages of affected individuals were calculated for the overall population of 92 adolescents for all but gynaecological problems, where percentages were calculated based on the number of girls (n = 51) in the survey

**Table 12:** Impact of signs, symptoms and events in proxy children (n = 325)^a^

n, (%)	n affected^b^	Severely impacted	Moderately impacted	Mildly impacted	Very mildly impacted	Not impacted	I don’t know	Prefer not to say	Missing
Basilar invagination	12	0 (0)	4 (1)	1 (0.3)	2 (0.6)	3 (0.9)	1 (0.3)	0 (0)	1 (0.3)
Dental problems	166	24 (7)	63 (19)	44 (14)	13 (4)	3 (0.9)	1 (0.3)	1 (0.3)	18 (6)
Eye or vision problems	69	6 (2)	19 (6)	20 (6)	11 (3)	4 (1)	2 (0.6)	2 (0.6)	7 (2)
Fatigue	170	26 (8)	63 (19)	46 (14)	13 (4)	2 (0.6)	2 (0.6)	0 (0)	18 (6)
Fractures	243	97 (30)	87 (27)	29 (9)	5 (2)	1 (0.3)	1 (0.3)	1 (0.3)	23 (7)
Gynaecological and menstruation problems	2	0 (0)	1 (0.7)	1 (0.7)	0 (0)	0 (0)	0 (0)	0 (0)	0 (0)
Hearing problems	20	3 (0.9)	3 (0.9)	5 (2)	2 (0.6)	4 (1)	0 (0)	0 (0)	3 (0.9)
Heart problems	11	1 (0.3)	1 (0.3)	4 (1)	1 (0.3)	4 (1)	0 (0)	0 (0)	0 (0)
High blood pressure	1	0 (0)	0 (0)	1 (0.3)	0 (0)	0 (0)	0 (0)	0 (0)	0 (0)
Hypermobility	139	9 (3)	47 (14)	26 (8)	15 (5)	16 (5)	5 (2)	0 (0)	21 (6)
Joint problems	47	8 (2)	12 (4)	10 (3)	9 (3)	1 (0.3)	1 (0.3)	1 (0.3)	5 (2)
Kidney and bladder problems	18	0 (0)	4 (1)	10 (3)	3 (0.9)	0 (0)	0 (0)	0 (0)	1 (0.3)
Low and underweight	78	17 (5)	19 (6)	15 (5)	9 (3)	9 (3)	0 (0)	0 (0)	9 (3)
Lung or breathing problems	24	7 (2)	10 (3)	5 (1)	1 (0.3)	0 (0)	1 (0.3)	0 (0)	0 (0)
Mental health problems	86	20 (6)	33 (10)	14 (4)	5 (2)	0 (0)	0 (0)	0 (0)	14 (4)
Obesity and overweight	32	5 (2)	11 (3)	10 (3)	1 (0.3)	1 (0.3)	0 (0)	0 (0)	4 (1)
Pain	261	60 (18)	108 (33)	48 (15)	12 (4)	3 (0.9)	2 (0.6)	0 (0)	28 (9)
Problems with chewing, swallowing, and speaking	41	10 (3)	11 (4)	11 (4)	5 (2)	1 (0.3)	0 (0)	0 (0)	3 (0.9)
Scoliosis or other bone problems	105	27 (8)	32 (10)	21 (6)	7 (2)	5 (2)	3 (0.9)	0 (0)	10 (3)
Sleep disturbance	73	20 (6)	27 (8)	12 (4)	4 (1)	0 (0)	0 (0)	0 (0)	10 (3)
Soft tissue problems or injuries	132	16 (5)	53 (16)	33 (10)	9 (3)	1 (0.3)	5 (2)	0 (0)	15 (5)
Stomach and bowel problems	122	15 (5)	43 (13)	34 (10)	11 (3)	5 (2)	1 (0.3)	0 (0)	13 (4)

OI, osteogenesis imperfecta

^a^Questions 188, 191, 193, 194, 195, 197–215 “In the past 12 months, how has x impacted your child’s life”, where x is any clinical sign, symptom or event participants reported to have experienced in the past 12 month in questions 187. Respondents chose a single answer option from a 5-level Likert scale “Not at all impacted–Severely impacted” and I don’t know, prefer not to say. ^b^Based on responses to question 187. Percentages of affected individuals were calculated for the overall population of 325 children for all but gynaecological problems, where percentages were calculated based on the number of girls (n = 137) in the survey

**Table 13:** Impact of signs, symptoms and events in proxy adolescents (n = 118)^a^

n, (%)	n affected^b^	Severely impacted	Moderately impacted	Mildly impacted	Very mildly impacted	Not impacted	I don’t know	Prefer not to say	Missing
Basilar invagination	9	0 (0)	4 (3)	2 (2)	0 (0)	1 (0.9)	0 (0)	0 (0)	2 (2)
Dental problems	52	6 (5)	21 (18)	14 (12)	3 (3)	1 (0.9)	0 (0)	0 (0)	7 (6)
Eye or vision problems	21	3 (3)	5 (4)	7 (6)	3 (3)	1 (0.9)	0 (0)	0 (0)	2 (2)
Fatigue	74	13 (11)	25 (21)	16 (14)	7 (6)	1 (0.9)	0 (0)	0 (0)	12 (10)
Fractures	74	22 (19)	23 (19)	18 (15)	1 (0.9)	0 (0)	0 (0)	0 (0)	10 (8)
Gynaecological and menstruation problems	7	1 (2)	6 (11)	0 (0)	0 (0)	0 (0)	0 (0)	0 (0)	0 (0)
Hearing problems	9	2 (2)	3 (3)	1 (0.9)	3 (3)	0 (0)	0 (0)	0 (0)	0 (0)
Heart problems	3	0 (0)	1 (0.9)	1 (0.9)	1 (0.9)	0 (0)	0 (0)	0 (0)	0 (0)
High blood pressure	0	0 (0)	0 (0)	0 (0)	0 (0)	0 (0)	0 (0)	0 (0)	0 (0)
Hypermobility	50	6 (5)	11 (9)	10 (8)	8 (7)	7 (6)	1 (0.9)	0 (0)	7 (6)
Joint problems	31	5 (4)	13 (11)	5 (4)	2 (2)	1 (0.9)	0 (0)	0 (0)	5 (4)
Kidney and bladder problems	6	0 (0)	6 (5)	0 (0)	0 (0)	0 (0)	0 (0)	0 (0)	0 (0)
Low and underweight	16	2 (2)	4 (3)	7 (6)	1 (0.9)	0 (0)	0 (0)	0 (0)	2 (2)
Lung or breathing problems	11	3 (3)	4 (3)	3 (3)	1 (0.9)	0 (0)	0 (0)	0 (0)	0 (0)
Mental health problems	37	7 (6)	17 (14)	5 (4)	2 (2)	0 (0)	0 (0)	0 (0)	6 (5)
Obesity and overweight	22	5 (4)	8 (7)	3 (3)	0 (0)	3 (3)	0 (0)	0 (0)	3 (3)
Pain	108	22 (19)	42 (36)	19 (16)	8 (7)	1 (0.9)	0 (0)	0 (0)	16 (14)
Problems with chewing, swallowing, and speaking	4	3 (3)	0 (0)	1 (0.9)	0 (0)	0 (0)	0 (0)	0 (0)	0 (0)
Scoliosis or other bone problems	82	25 (21)	24 (20)	15 (13)	5 (4)	2 (2)	1 (0.9)	0 (0)	10 (8)
Sleep disturbance	29	8 (7)	14 (12)	3 (3)	0 (0)	0 (0)	0 (0)	0 (0)	4 (3)
Soft tissue problems or injuries	71	4 (3)	29 (25)	18 (15)	7 (6)	5 (4)	0 (0)	0 (0)	7 (6)
Stomach and bowel problems	34	5 (4)	12 (10)	10 (8)	3 (3)	1 (0.9)	0 (0)	0 (0)	3 (3)

OI, osteogenesis imperfecta

^a^Questions 319, 321, 323, 324, 325, 327–345 “In the past 12 months, how has x impacted your life”, where x is any clinical sign, symptom or event participants reported to have experienced in the past 12 month in questions 318. Respondents chose a single answer option from a 5-level Likert scale “Not at all impacted–Severely impacted” and I don’t know, prefer not to say. ^b^Based on responses to question 318. Percentages of affected individuals were calculated for the overall population of 118 adolescents for all but gynaecological problems, where percentages were calculated based on the number of girls (n = 57) in the survey

**Table 14:** Impact of signs, symptoms and events in proxy adults (n = 85)^a^

n, (%)	n affected^b^	Severely impacted	Moderately impacted	Mildly impacted	Very mildly impacted	Not impacted	I don’t know	Prefer not to say	Missing
Basilar invagination	4	1 (1)	0 (0)	1 (1)	0 (0)	0 (0)	2 (2)	0 (0)	0 (0)
Dental problems	35	9 (11)	16 (19)	8 (9)	1 (1)	0 (0)	0 (0)	0 (0)	1 (1)
Eye or vision problems	16	0 (0)	5 (6)	8 (9)	3 (4)	0 (0)	0 (0)	0 (0)	0 (0)
Fatigue	47	8 (9)	21 (25)	12 (14)	2 (2)	0 (0)	0 (0)	0 (0)	4 (5)
Fractures	26	4 (5)	14 (16)	2 (2)	1 (1)	1 (1)	1 (1)	0 (0)	3 (4)
Gynaecological and menstruation problems	7	1 (3)	5 (13)	0 (0)	0 (0)	0 (0)	0 (0)	0 (0)	1 (3)
Hearing problems	12	1 (1)	6 (7)	0 (0)	3 (4)	1 (1)	0 (0)	0 (0)	1 (1)
Heart problems	1	1 (1)	0 (0)	0 (0)	0 (0)	0 (0)	0 (0)	0 (0)	0 (0)
High blood pressure	7	0 (0)	4 (5)	1 (1)	2 (2)	0 (0)	0 (0)	0 (0)	0 (0)
Hypermobility	21	0 (0)	6 (7)	9 (11)	3 (4)	1 (1)	0 (0)	0 (0)	2 (2)
Joint problems	15	2 (2)	7 (8)	4 (5)	1 (1)	0 (0)	0 (0)	0 (0)	1 (1)
Kidney and bladder problems	4	1 (1)	2 (2)	0 (0)	1 (1)	0 (0)	0 (0)	0 (0)	0 (0)
Low and underweight	8	3 (4)	0 (0)	1 (1)	0 (0)	3 (4)	0 (0)	0 (0)	1 (1)
Lung or breathing problems	12	5 (6)	4 (5)	3 (4)	0 (0)	0 (0)	0 (0)	0 (0)	0 (0)
Mental health problems	31	12 (14)	11 (13)	5 (6)	1 (1)	0 (0)	0 (0)	0 (0)	2 (2)
Obesity and overweight	24	7 (8)	9 (11)	6 (7)	2 (2)	0 (0)	0 (0)	0 (0)	0 (0)
Pain	72	17 (20)	30 (35)	11 (13)	6 (7)	0 (0)	1 (1)	0 (0)	7 (8)
Problems with chewing, swallowing, and speaking	11	4 (5)	4 (5)	0 (0)	2 (2)	1 (1)	0 (0)	0 (0)	0 (0)
Scoliosis or other bone problems	38	17 (20)	10 (12)	6 (7)	2 (2)	1 (1)	0 (0)	0 (0)	2 (2)
Sleep disturbance	30	5 (6)	18 (21)	5 (6)	1 (1)	0 (0)	0 (0)	0 (0)	1 (1)
Soft tissue problems or injuries	37	3 (4)	16 (19)	9 (11)	2 (2)	1 (1)	2 (2)	0 (0)	4 (5)
Stomach and bowel problems	21	4 (5)	9 (11)	4 (5)	2 (2)	0 (0)	0 (0)	0 (0)	2 (2)

OI, osteogenesis imperfecta

^a^Questions 319, 321, 323, 324, 325, 327–345 “In the past 12 months, how has x impacted your life”, where x is any clinical sign, symptom or event participants reported to have experienced in the past 12 month in questions 318. Respondents chose a single answer option from a 5-level Likert scale “Not at all impacted–Severely impacted” and I don’t know, prefer not to say. ^b^Based on responses to question 318. Percentages of affected individuals were calculated for the overall population of 85 adults for all but gynaecological problems, where percentages were calculated based on the number of women (n = 39) in the survey
