# Supplementary material for: The IMPACT survey: a mixed methods study to understand the experience of children, adolescents and adults with osteogenesis imperfecta and their caregivers
Source: Orphanet J Rare Dis. 2024 Mar 21;19:128. doi: 10.1186/s13023-024-03126-9 (PMC10956293; doi:10.1186/s13023-024-03126-9)
Supplement: Supplementary file 1 — Additional file 1. IMPACT Survey Questionnaire. [file 13023_2024_3126_MOESM1_ESM.docx]

| **IMPACT questionnaire** |
| --- |

Table 1 IMPACT Questionnaire with 377 questions. Populations for which each question was available are indicated by an x in respective columns; Abbreviations: CR: care recipient

| No | Question | Instruction | Multi choice | Answer options | Free text field | Adult with OI | Adolescent with OI | Caregivers with OI | | | Caregivers without OI | | | Relative |
| --- | --- | --- | --- | --- | --- | --- | --- | --- | --- | --- | --- | --- | --- | --- |
|  |  |  |  |  |  |  |  | 1 CR | 2 CR | 3 CR | 1 CR | 2 CR | 3 CR |  |
| 1 | What is your age? (years) |  | No | Younger than 12 Integer (12 to 100) | No | x | x | x | x | x | x | x | x | x |
| 2 | Please indicate which of the following best describes you: | Please select only one answer | No | I am a person with OI I am the parent or caregiver of a child/children with OI I am a person with OI and the parent or caregiver of a child/children with OI I am a parent of an adult with OI None of the above | No | x | x | - | - | - | - | - | - | - |
| 3 | Do you wish to continue? | Please select only one answer | No | Yes No | No | - | - | x | x | x | x | x | x | x |
| 4 | Has another person (for example caregiver, parent, spouse, sibling) already completed this survey about your OI (if applicable)? | Please select only one answer | No | Yes, someone has already completed the survey about my OI No, but someone is planning on completing the survey about my OI No I don’t know | No | x | x | - | - | - | - | - | - | - |
| 5 | Has your child or another parent or caregiver completed the survey about your child's/children's OI? | Please select only one answer | No | Yes, my child/children have completed the survey about their OI Yes, my child's/children's other parent has completed the survey about my child’s/children's OI No, but my child/children are planning on completing the survey about their OI No, but my child's/children's other parent is planning on completing the survey about my child's/children's OI No I don't know | No | - | - | - | - | - | - | - | - | - |
| 6 | Has your child or another parent or caregiver completed this survey about your child's/children's OI? | Please select only one answer | No | Yes, my child/children have completed the survey about their OI Yes, my child's/children's other parent has completed the survey about my child's/children's OI No, but my child/children are planning on completing the survey about their OI No, but my child's/children's other parent is planning on completing the survey about my child's/children's OI No I don't know | No | - | - | x | x | x | - | - | - | - |
| 7 | What is your country of residence? |  | No | Afghanistan – Zimbabwe (195 alternatives) | No | x | - | - | - | - | x | x | x | - |
| 8 | What is your sex? | Please select only one answer | No | Male Female Other Prefer not to say | No | x | - | x | x | x | x | x | x | - |
| 9 | Please indicate which of the following best describe you. | Please select all that apply to you | Yes | I am a student I am in paid employment/self-employed I am retired because of my age I am retired because of my disability I am a homemaker (housewife/househusband) I am a volunteer I am between jobs I am unable to find a job I am not employed I am not fit to work Prefer not to say Other (please specify):   - Other (please specify): | Yes | x | - | x | x | x | x | x | x | - |
| 10 | What is your current paid employment status? | Please select only one answer | No | Employed full time Employed part time Self-employed Prefer not to say  Other (please specify):   - Other (please specify): | Yes | x | - | x | x | x | x | x | x | - |
| 11 | Who do you live with? | Please select all that apply to you | Yes | I live alone I live with my partner/spouse I live with my parents (and other family members if applicable) I live with my partner and our child/children I am a single parent living with my child/children I live with friends (or house share) I live with my caregiver/assistant I live in supported living accommodation or a care home Prefer not to say Other (please specify):   - Other (please specify): | Yes | x | - | x | x | x | x | x | x | - |
| 12 | Who do you live with? | Please select all that apply and note here and for the duration of the survey 'my child/children' will refer to the child/children that you care for | Yes | I live with my partner/spouse and our child/children I live with my parents and my child/children (and other family members if applicable)  I am a single parent living with my child/children I live with friends (or house share) and my child/children I live with my caregiver/assistant and my child/children I live in supported living accommodation or a care home with my child/children Prefer not to say Other (please specify):   - Other (please specify): | Yes | - | - | - | - | - | - | - | - | - |
| 13 | Do you prefer the metric or imperial system to describe your height? | Please select only one answer | No | Metric (centimetres) Imperial (feet/inches) | No | x | - | - | - | - | x | x | x | - |
| 14 | What is your height? | Please select your height in centimetres | No | I don't know Prefer not to say Integer (40 to 200) | No | x | - | - | - | - | x | x | x | - |
| 15 | What is your height? | Please select your height in feet and inches | No | I don't know Prefer not to say Integer (1 feet, 4 inches to 6 feet, 8 inches) | No | x | - | - | - | - | x | x | x | - |
| 16 | How do you get around? | Please select all that apply | Multiple answers per row | Columns: Inside your home Outside your home Rows: Walking unaided Cane/walking stick Walking frame Rollator (wheeled walker) Crutches Manual wheelchair Powered wheelchair Mobility scooter Crawling Being carried Laying in bed/stretcher Other (please specify below)   - Other: | Yes | x | - | - | - | - | x | x | x | - |
| 17 | If you have received an OI type as part of your OI diagnosis or treatment, please indicate your type using the dropdown below. | Please note: If you were not diagnosed with a specific type, please select 'Undefined type', if you do not know your type please select 'I don't know'. You may have been diagnosed with a specific OI type even if you did not have genetic testing. In all cases we will use your responses to other questions in this survey to understand more about the kind of OI you have. | No | Undefined type I don't know Prefer not to say Type 1 (I) - Type 18 (XVIII) Other   - Other (please specify) | Yes | x | - | - | - | - | x | x | x | - |
| 18 | How would you describe the severity of your OI? | Please select only one answer | No | Mild Moderate Severe I don't know Prefer not to say | No | x | - | - | - | - | x | x | x | - |
| 19 | Do you have a genetically confirmed diagnosis of OI? | Please select only one answer | No | Yes No I don't know Prefer not to say | No | x | - | - | - | - | x | x | x | - |
| 20 | Why is your OI not genetically confirmed? | Please select all that apply | Yes | My test was inconclusive I do not want a test The test was too expensive for me Genetic testing is not available in my country I wasn't offered a test I don't know Prefer not to say For other reasons (please specify):   - For other reasons (please specify): | Yes | x | - | - | - | - | x | x | x | - |
| 21 | Which gene is the cause of your OI diagnosis? | Please select all that apply | Yes | BMP1 COL1A1 COL1A2 CREB3L1 CRTAP FKBP10 IFITM5 LEPRE1/P3H1 MBTPS2 MESD P4HB PLOD2 PLS3 PPIB SEC24D SERPINF1 SERPINH1 SP7 SPARC TENT5A TMEM38 WNT1 Other I don't know Prefer not to say   - Other (please specify) | Yes | x | - | - | - | - | x | x | x | - |
| 22 | How many children with OI do you live with? |  | No | Integer (0 to 3) | No | - | - | - | - | - | x | x | x | - |
| 23 | How many children without OI do you live with? |  | No | Integer (0 to 4) | No | - | - | x | x | x | x | x | x | - |
| 24 | What is your relationship to the child/children with OI in your care? | Please select all that apply | Yes | Parent Sibling Grandparent Legal guardian Prefer not to say Other family member (please specify):   - Other family member (please specify): | Yes | - | - | x | x | x | x | x | x | - |
| 25 | Who do you live with? | Please select all that apply and note here and for the duration of the survey 'my child/children' will refer to the child/children that you care for | Yes | I live with my partner and our child/children I am a single parent living with my child/children I live with my parents (and other family members if applicable) and my child/children I live with friends (or house share) and my child/children Prefer not to say Other (please specify):   - Other (please specify): | Yes | - | - | x | x | x | x | x | x | - |
| 26 | Do you prefer the metric or imperial system to describe your child's height? | Please select only one answer | No | Metric (centimetres) Imperial (feet/inches) | No | - | - | x | x | x | - | x | - | - |
| 27 | What is your child's height? | Please select the height of your child with OI in centimetres | No | I don't know Prefer not to say Integer (20 to 200) | No | - | - | x | - | - | - | - | - | - |
| 28 | What is your child's height? | Please select the height of your child with OI in feet and inches | No | I don't know Prefer not to say Integer   10 inches   11 inches   1 foot  1 foot, 1 inch to 6 feet, 8 inches | No | - | - | x | - | - | x | - | - | - |
| 29 | What is the age of your child? (years) |  | No | Prefer not to say  <1 Integer (1 to 70) | No | - | - | x | - | - | x | - | - | - |
| 30 | What is the sex of your child? | Please select only one answer | No | Male Female Other Prefer not to say | No | - | - | x | - | - | x | - | - | - |
| 31 | If your child has received an OI type as part of their OI diagnosis or treatment, please select their type using the dropdown menu below. | Please note: If they were not diagnosed with a specific type, please select 'Undefined type', if you do not know their type please select 'I don't know'. They may have been diagnosed with a specific OI type even if they did not have genetic testing. In all cases we will use your responses to other questions in this survey to understand more about the kind of OI your child has. | No | Undefined type I don't know Prefer not to say Type 1 (I)- Type 18 (XVIII) Other   - Other (please specify) | Yes | - | - | x | - | - | x | - | - | - |
| 32 | How would you describe the severity of your child's OI? | Please select only one answer | No | Mild Moderate Severe I don't know Prefer not to say | No | - | - | x | - | - | x | - | - | - |
| 33 | Does your child have a genetically confirmed diagnosis of OI? | Please select only one answer | No | Yes No I don't know Prefer not to say | No | - | - | x | - | - | x | - | - | - |
| 34 | Why is your child's OI not genetically confirmed? | Please select all that apply | Yes | Their test was inconclusive We do not want a test The test was too expensive for me Genetic testing is not available in my country They were not offered a test I don't know Prefer not to say For other reasons (please specify):   - For other reasons (please specify): | Yes | - | - | x | - | - | x | - | - | - |
| 35 | Which gene is the cause of your child's OI diagnosis? |  | No | BMP1 COL1A1 COL1A2 CREB3L1 CRTAP FKBP10 IFITM5 LEPRE1/P3H1 MBTPS2 MESD P4HB PLOD2 PLS3 PPIB SEC24D SERPINF1 SERPINH1 SP7 SPARC TENT5A TMEM38 WNT1 Other I don't know Prefer not to say   - Other (please specify) | Yes | - | - | x | - | - | x | - | - | - |
| 36 | How does your child get around? | Please select all that apply to each child | Yes | Columns:  Child 1 Inside your home Child 1 Outside your home Child 2 Inside your home Child 2 Outside your home Rows: Walking unaided Cane/walking stick Rollator (wheeled walker) Walking frame Crutches Manual wheelchair Powered wheelchair Mobility scooter Crawling Being carried Laying in bed/stretcher Other (please specify below)   - Other: | Yes | - | - | x | - | - | x | - | - | - |
| 37 | Does your child attend school? | Please select only one answer for each child | Yes | Columns:  Child 1 Child 2  Rows:  Yes, my child attends our school of choice Yes, my child attends school but not our school of choice No, my child is home schooled No, my child is not of school age Prefer not to say Other (please specify below)   - Other (please specify) | Yes | - | - | x | - | - | x | - | - | - |
| 38 | Why can't your child attend your school of choice? | Please select all that apply | Yes | Our school of choice does not provide adequate facilities to cater for my child’s accessibility needs Our school of choice will not accept my child because of their needs For reasons that are not related to my child's OI I don't know Prefer not to say Other (please specify):   - Other (please specify): | Yes | - | - | x | - | - | x | - | - | - |
| 39 | Why do you feel home schooling is the best option for your child? | Please select all that apply | Yes | My child’s healthcare needs mean that they miss too many school days and fall behind Schools do not provide adequate facilities to cater for my child’s accessibility needs My/their school of choice will not accept my child because of their needs I am concerned about my child’s safety For reasons that are not related to my child's OI I don't know Prefer not to say Other (please specify):  - Other (please specify): | Yes | - | - | x | - | - | x | - | - | - |
| 40 | What is the age of your children? |  | No | Column: Age (years)  Prefer not to say   <1  Integer (1 to 60) Rows: Child 1 Child 2 | No | - | - | x | - | - | x | - | - | - |
| 41 | What is the sex of your children? |  | No | Column: Sex  Male Female Other  Prefer not to say Rows: Child 1 Child 2 | No | - | - | - | x | - | - | x | - | - |
| 42 | Do you prefer the metric or imperial system to describe your children's height? Please select only one answer | None | No | Metric (centimetres) Imperial (feet/inches) | No | - | - | - | x | - | - | x | - | - |
| 43 | What is your children's height? | Please select the height of your children with OI in feet and inches | No | Column: Height (feet)  I don't know Prefer not to say Integer 10 inches 11 inches 1 foot 1 foot, 1 inch to 6 feet, 5 inches Rows: Child 1 Child 2 | No | - | - | - | x | - | - | - | - | - |
| 44 | What is the height of your children? | Please select the height of your children with OI with centimetres | No | Column: Height (centimetres)  I don't know  Prefer not to say  Integer (20 to 200) Rows: Child 1 Child 2 | No | - | - | - | x | - | - | x | - | - |
| 45 | If your children have received an OI type as part of their OI diagnosis or treatment, please indicate their type using the dropdown below. | Please note: If they were not diagnosed with a specific type, please select 'Undefined type', if you do not know your children's type please select 'I don't know'. They may have been diagnosed with a specific OI type even if they did not have genetic testing. In all cases, we will use your responses to other questions in this survey to understand more about the kind of OI your children experience. | No | Column: OI type  Undefined type  I don't know  Type 1 (I) – Type 18 (XVIII)  Prefer not to say Rows: Child 1 Child 2 | No | - | - | - | x | - | - | x | - | - |
| 46 | How would describe the severity of your children's OI? |  | No | Column: Severity  Mild Moderate Severe I don't know Prefer not to say Rows: Child 1 Child 2 | No | - | - | - | x | - | - | x | - | - |
| 47 | Do your children have a genetically confirmed diagnosis of OI? | Please select one answer for each child | No | Columns:  Child 1 Child 2 Rows:  Yes No I don't know Prefer not to say   - Other (please specify): | Yes | - | - | - | x | - | - | x | - | - |
| 48 | What is the age of your children? |  | No | Column: Age (years)  Prefer not to say   <1  Integer (1 to 60) Rows: Child 1 Child 2 Child 3 | No | - | - | - | x | - | - | x | - | - |
| 49 | What is the sex of your children? |  | No | Column: Sex  Male Female Other  Prefer not to say Rows: Child 1 Child 2 Child 3 | No | - | - | - | - | x | - | - | x | - |
| 50 | Do you prefer the metric or imperial system to describe your children's height? Please select only one answer | None | No | Metric (centimetres) Imperial (feet/inches) | No | - | - | - | - | x | - | - | x | - |
| 51 | What is your children's height? | Please select the height of your children with OI in feet and inches | No | Column: Height (feet)  I don't know Prefer not to say Integer 10 inches 11 inches 1 foot 1 foot, 1 inch to 6 feet, 5 inches Rows: Child 1 Child 2 Child 3 | No | - | - | - | - | x | - | - | x | - |
| 52 | What is the height of your children? | Please select the height of your children with OI with centimetres | No | Column: Height (centimetres)  I don't know  Prefer not to say  Integer (20 to 200) Rows: Child 1 Child 2 Child 3 | No | - | - | - | - | x | - | - | x | - |
| 53 | If your children have received an OI type as part of their OI diagnosis or treatment, please indicate their type using the dropdown below. | Please note: If they were not diagnosed with a specific type, please select 'Undefined type', if you do not know your children's type please select 'I don't know'. They may have been diagnosed with a specific OI type even if they did not have genetic testing. In all cases, we will use your responses to other questions in this survey to understand more about the kind of OI your children experience. | No | Column: OI type  Undefined type  I don't know  Type 1 (I) - Type 18 (XVIII)  Prefer not to say Rows: Child 1 Child 2 Child 3 | No | - | - | - | - | x | - | - | x | - |
| 54 | How would describe the severity of your children's OI? |  | No | Column: Severity  Mild Moderate Severe I don't know Prefer not to say Rows: Child 1 Child 2 Child 3 | No | - | - | - | - | x | - | - | x | - |
| 55 | Do your children have a genetically confirmed diagnosis of OI? | Please select one answer for each child | No | Columns:  Child 1 Child 2 Child 3 Rows:  Yes No I don't know Prefer not to say   - Other (please specify): | Yes | - | - | - | - | x | - | - | x | - |
| 56 | Why is child 1's OI not genetically confirmed? | Please select all that apply | Yes | Their test was inconclusive We do not want a test The test was too expensive for me Genetic testing is not available in my country They were not offered a test I don't know Prefer not to say For other reasons (please specify):   - For other reasons (please specify): | Yes | - | - | - | - | x | - | - | x | - |
| 57 | Which gene is the cause of the OI diagnosis of child 1? |  | No | BMP1 COL1A1 COL1A2 CREB3L1 CRTAP FKBP10 IFITM5 LEPRE1/P3H1 MBTPS2 MESD P4HB PLOD2 PLS3 PPIB SEC24D SERPINF1 SERPINH1 SP7 SPARC TENT5A TMEM38 WNT1 Other I don't know Prefer not to say   - Other (please specify) | Yes | - | - | - | x | - | - | x | - | - |
| 58 | Why is child 1's OI not genetically confirmed? | Please select all that apply | Yes | Their test was inconclusive We do not want a test The test was too expensive for me Genetic testing is not available in my country They were not offered a test I don't know Prefer not to say For other reasons (please specify):   - For other reasons (please specify): | Yes | - | - | - | x | - | - | x | - | - |
| 59 | Which gene is the cause of the OI diagnosis of child 1? |  | No | BMP1 COL1A1 COL1A2 CREB3L1 CRTAP FKBP10 IFITM5 LEPRE1/P3H1 MBTPS2 MESD P4HB PLOD2 PLS3 PPIB SEC24D SERPINF1 SERPINH1 SP7 SPARC TENT5A TMEM38 WNT1 Other I don't know Prefer not to say   - Other (please specify) | Yes | - | - | - | - | x | - | - | x | - |
| 60 | Why is child 2's OI not genetically confirmed? | Please select all that apply | Yes | Their test was inconclusive We do not want a test The test was too expensive for me Genetic testing is not available in my country They were not offered a test I don't know Prefer not to say For other reasons (please specify):   - For other reasons (please specify): | Yes | - | - | - | - | x | - | - | x | - |
| 61 | Which gene is the cause of the OI diagnosis of child 2? |  | No | BMP1 COL1A1 COL1A2 CREB3L1 CRTAP FKBP10 IFITM5 LEPRE1/P3H1 MBTPS2 MESD P4HB PLOD2 PLS3 PPIB SEC24D SERPINF1 SERPINH1 SP7 SPARC TENT5A TMEM38 WNT1 Other I don't know Prefer not to say   - Other (please specify) | Yes | - | - | - | x | - | - | x | - | - |
| 62 | Why is child 2's OI not genetically confirmed? | Please select all that apply | Yes | Their test was inconclusive We do not want a test The test was too expensive for me Genetic testing is not available in my country They were not offered a test I don't know Prefer not to say For other reasons (please specify):   - For other reasons (please specify): | Yes | - | - | - | x | - | - | x | - | - |
| 63 | Why is child 3's OI not genetically confirmed? | Please select all that apply | Yes | Their test was inconclusive We do not want a test The test was too expensive for me Genetic testing is not available in my country They were not offered a test I don't know Prefer not to say For other reasons (please specify):   - For other reasons (please specify): | Yes | - | - | - | - | x | - | - | x | - |
| 64 | Which gene is the cause of the OI diagnosis of child 2? |  | No | BMP1 COL1A1 COL1A2 CREB3L1 CRTAP FKBP10 IFITM5 LEPRE1/P3H1 MBTPS2 MESD P4HB PLOD2 PLS3 PPIB SEC24D SERPINF1 SERPINH1 SP7 SPARC TENT5A TMEM38 WNT1 Other I don't know Prefer not to say   - Other (please specify) | Yes | - | - | - | - | x | - | - | x | - |
| 65 | Which gene is the cause of the OI diagnosis of child 3? |  | No | BMP1 COL1A1 COL1A2 CREB3L1 CRTAP FKBP10 IFITM5 LEPRE1/P3H1 MBTPS2 MESD P4HB PLOD2 PLS3 PPIB SEC24D SERPINF1 SERPINH1 SP7 SPARC TENT5A TMEM38 WNT1 Other I don't know Prefer not to say   - Other (please specify) | Yes | - | - | - | - | x | - | - | x | - |
| 66 | How do your children get around? | Please select all that apply to each child | Yes | Columns:  Child 1 Inside your home Child 1 Outside your home Child 2 Inside your home Child 2 Outside your home Rows: Walking unaided Cane/walking stick Rollator (wheeled walker) Walking frame Crutches Manual wheelchair Powered wheelchair Mobility scooter Crawling Being carried Laying in bed/stretcher Other (please specify below)   - Other: | Yes | - | - | - | - | x | - | - | x | - |
| 67 | How do your children get around? | Please select all that apply to each child | Yes | Columns:  Child 1 Inside your home Child 1 Outside your home Child 2 Inside your home Child 2 Outside your home Rows: Walking unaided Cane/walking stick Rollator (wheeled walker) Walking frame Crutches Manual wheelchair Powered wheelchair Mobility scooter Crawling Being carried Laying in bed/stretcher Other (please specify below)   - Other: | Yes | - | - | - | x | - | - | x | - | - |
| 68 | Do your children attend school? | Please select only one answer for each child | Yes | Columns:  Child 1 Child 2  Rows:  Yes, my child attends our school of choice Yes, my child attends school but not our school of choice No, my child is home schooled No, my child is not of school age Prefer not to say Other (please specify below)   - Other (please specify) | Yes | - | - | - | - | x | - | - | x | - |
| 69 | Do your children attend school? | Please select only one answer for each child | Yes | Columns:  Child 1 Child 2 Child 3  Rows:  Yes, my child attends our school of choice Yes, my child attends school but not our school of choice No, my child is home schooled No, my child is not of school age Prefer not to say Other (please specify below)   - Other (please specify) | Yes | - | - | - | x | - | - | x | - | - |
| 70 | Why can't child 1 attend your school of choice? | Please select all that apply | Yes | Our school of choice does not provide adequate facilities to cater for my child’s accessibility needs Our school of choice will not accept my child because of their needs For reasons that are not related to my child's OI I don't know Prefer not to say Other (please specify):   - Other (please specify): | Yes | - | - | - | - | x | - | - | x | - |
| 71 | Why can't child 1 attend your school of choice? | Please select all that apply | Yes | Our school of choice does not provide adequate facilities to cater for my child’s accessibility needs Our school of choice will not accept my child because of their needs For reasons that are not related to my child's OI I don't know Prefer not to say Other (please specify):   - Other (please specify): | Yes | - | - | - | x | - | - | x | - | - |
| 72 | Why can't child 2 attend your school of choice? | Please select all that apply | Yes | Our school of choice does not provide adequate facilities to cater for my child’s accessibility needs Our school of choice will not accept my child because of their needs For reasons that are not related to my child's OI I don't know Prefer not to say Other (please specify):   - Other (please specify): | Yes | - | - | - | - | x | - | - | x | - |
| 73 | Why can't child 2 attend your school of choice? | Please select all that apply | Yes | Our school of choice does not provide adequate facilities to cater for my child’s accessibility needs Our school of choice will not accept my child because of their needs For reasons that are not related to my child's OI I don't know Prefer not to say Other (please specify):   - Other (please specify): | Yes | - | - | - | x | - | - | x | - | - |
| 74 | Why can't child 3 attend your school of choice? | Please select all that apply | Yes | Our school of choice does not provide adequate facilities to cater for my child’s accessibility needs Our school of choice will not accept my child because of their needs For reasons that are not related to my child's OI I don't know Prefer not to say Other (please specify):   - Other (please specify): | Yes | - | - | - | - | x | - | - | x | - |
| 75 | Why do you feel home schooling is the best option for child 1? | Please select all that apply | Yes | My child’s healthcare needs mean that they miss too many school days and fall behind Schools do not provide adequate facilities to cater for my child’s accessibility needs My/their school of choice will not accept my child because of their needs I am concerned about my child’s safety For reasons that are not related to my child's OI I don't know Prefer not to say Other (please specify):  - Other (please specify): | Yes | - | - | - | - | x | - | - | x | - |
| 76 | Why do you feel home schooling is the best option for child 1? | Please select all that apply | Yes | My child’s healthcare needs mean that they miss too many school days and fall behind Schools do not provide adequate facilities to cater for my child’s accessibility needs My/their school of choice will not accept my child because of their needs I am concerned about my child’s safety For reasons that are not related to my child's OI I don't know Prefer not to say Other (please specify):  - Other (please specify): | Yes | - | - | - | x | - | - | x | - | - |
| 77 | Why do you feel home schooling is the best option for child 2? | Please select all that apply | Yes | My child’s healthcare needs mean that they miss too many school days and fall behind Schools do not provide adequate facilities to cater for my child’s accessibility needs My/their school of choice will not accept my child because of their needs I am concerned about my child’s safety For reasons that are not related to my child's OI I don't know Prefer not to say Other (please specify):  - Other (please specify): | Yes | - | - | - | - | x | - | - | x | - |
| 78 | Why do you feel home schooling is the best option for child 2? | Please select all that apply | Yes | My child’s healthcare needs mean that they miss too many school days and fall behind Schools do not provide adequate facilities to cater for my child’s accessibility needs My/their school of choice will not accept my child because of their needs I am concerned about my child’s safety For reasons that are not related to my child's OI I don't know Prefer not to say Other (please specify):  - Other (please specify): | Yes | - | - | - | x | - | - | x | - | - |
| 79 | Why do you feel home schooling is the best option for child 3? | Please select all that apply | Yes | My child’s healthcare needs mean that they miss too many school days and fall behind Schools do not provide adequate facilities to cater for my child’s accessibility needs My/their school of choice will not accept my child because of their needs I am concerned about my child’s safety For reasons that are not related to my child's OI I don't know Prefer not to say Other (please specify):  - Other (please specify): | Yes | - | - | - | - | x | - | - | x | - |
| 80 | In the past 12 months, how would you describe the impact that caring for a child with OI has had on your life? | This question is about understanding the ‘negative’ impacts or challenges you have faced. We will ask you about any positive impacts later in the survey. Please select one answer per row. | Only one answer per row | Columns: Severely impacted Moderately impacted Mildly impacted Very mildly impacted Not impacted Not applicable I don't know Prefer not to say Rows: The number of hours you work in your paid job The type of job you can do Your career choices The amount of time you have free for leisure activities (for example, hobbies, sports, reading) Your social life (for example, socialising with friends, attending events, participating in activities) Your relationships with family and friends Your romantic relationships Your mental health Your physical health Your happiness   - Are there any other aspects of your life that you feel are impacted by caring for a child with OI? | Yes | - | - | - | - | x | - | - | x | - |
| 81 | Do you feel worried or concerned about any of the following things? | Please select one answer per row | Only one answer per row | Columns: Worry a lot Worry a little Don't worry Not applicable I don't know Prefer not to say Rows: Your child's future Your child's opportunities How your child's OI impact your other family members How your child's OI impacts your relationships with family and friends How your child's OI impacts your romantic relationships Your child not having access to doctors who understand their OI Your child not having access to medicines to manage their OI Your child not having access to care to manage their OI Side effects of the treatment your child receives Your financial situation in the future Your child's future transition from paediatric to adult care   - Do you have any other worries or concerns? | Yes | - | - | x | - | - | - | - | - | - |
| 82 | In the past 12 months, how would you describe the impact that caring for children with OI has had on your life? | This question is about understanding the ‘negative’ impacts or challenges you have faced. We will ask you about any positive impacts later in the survey. Please select one answer per row. | Only one answer per row | Columns: Severely impacted Moderately impacted Mildly impacted Very mildly impacted Not impacted Not applicable I don't know Prefer not to say Rows: The number of hours you work in your paid job The type of job you can do Your career choices The amount of time you have free for leisure activities (for example, hobbies, sports, reading) Your social life (for example, socialising with friends, attending events, participating in activities) Your relationships with family and friends Your romantic relationships Your mental health Your physical health Your happiness   - Are there any other aspects of your life that you feel are impacted by caring for children with OI? | Yes | - | - | x | - | - | - | - | - | - |
| 83 | Do you feel worried or concerned about any of the following things? | Please select one answer per row | Only one answer per row | Columns: Worry a lot Worry a little Don't worry Not applicable I don't know Prefer not to say Rows: Your children's future Your children's opportunities How your children's OI impacts your other family members How your children's OI impacts your relationships with family and friends How your children's OI impacts your romantic relationships Your children not having access to doctors who understand their OI Your children not having access to medicines to manage their OI Your children not having access to care to manage their OI Side effects of the treatment your children receive Your financial situation in the future Your children's future transition from paediatric to adult care   - Do you have any other worries or concerns? | Yes | - | - | - | x | x | - | - | - | - |
| 84 | How do you feel your child's OI impacts your other child/children without OI? | Please select one answer per row | Only one answer per row | Columns: Strongly agree Agree Neither agree nor disagree Disagree Strongly disagree Not applicable I don't know Prefer not to say  Rows: My other child/children miss out on leisure opportunities because of my caregiving responsibilities for my child with OI My other child/children are alone a lot because of my caregiving responsibilities for my child with OI My other child/children have to help out more with chores because of my caregiving responsibilities for my child with OI My other child/children worry about the wellbeing and safety of their sibling with OI My other child/children get less attention because of my caregiving responsibilities for my child with OI I feel that my other child/children are resentful towards me because I give more attention to my child with OI   - Are there any other aspects of your other child/children's life that you feel are impacted by OI? | Yes | - | - | - | x | x | - | - | - | - |
| 85 | How do you feel your children's OI impacts your other child/children without OI? | Please select one answer per row | Only one answer per row | Columns:  Strongly agree Agree Neither agree nor disagree Disagree Strongly disagree Not applicable I don't know Prefer not to say Rows: My other child/children miss out on leisure opportunities because of my caregiving responsibilities for my children with OI My other child/children are alone a lot because of my caregiving responsibilities for my children with OI My other child/children have to help out more with chores because of my caregiving responsibilities for my children with OI My other child/children worry about the wellbeing and safety of their siblings with OI My other child/children get less attention because of my caregiving responsibilities for my children with OI I feel that my other child/children are resentful towards me because I give more attention to my children with OI  - Are there any other aspects of your other child/children's life that you feel are impacted by OI? | Yes | - | - | x | - | - | - | - | - | - |
| 86 | In the past 4 weeks, how many days of work have you missed due to your care responsibilities for your child/children with OI? | None | No | 0 Less than 1 Integer (1 to 28) I don't know Prefer not to say | No | - | - | - | x | x | - | - | - | - |
| 87 | Please indicate your local currency | None | No | AED, (United Arab Emirates dirham) - ZMW, (Zambian kwacha) **(136 currencies)** | No | - | - | x | x | x | - | - | - | - |
| 88 | Is your child/children (with OI) covered by private health insurance? | Please select only one answer | No | Yes No I don't know Prefer not to say | No | - | - | x | x | x | - | - | - | - |
| 89 | Why is your child/children (with OI) not covered by private health insurance? | Please select all that apply | Yes | We don't want private health insurance We don't need private health insurance We can't afford private health insurance Private health insurance is not an option where we live We don't qualify for private health insurance because of their OI Prefer not to say I don't know Other reasons   - Other reasons: | Yes | - | - | x | x | x | - | - | - | - |
| 90 | How are your child's/children's (with OI) healthcare costs covered? | Please select all that apply | Yes | Public statutory insurance (for example TK or AOK in Germany) Private health insurance (for example United Healthcare in the US or Bupa in the UK) National healthcare provision (for example Medicaid, CHIP, Canadian Medicare or NHS) Charity funding I pay for healthcare myself (out of pocket) Financial support from family and friends Employer Other public funding (for example disability benefits, welfare, social security) Other funding (please specify):   - Other funding (please specify): | Yes | - | - | x | x | x | - | - | - | - |
| 91 | If your child/children with OI use any of the following things, who covers the cost? | Please select all that apply for each item they use. If they do not use an item, please select 'Not applicable'. | Multiple answers per row | Columns: Not applicable I pay myself Private health insurance Public statutory insurance Charity funding Family/friends National healthcare provision Employer Other public funding I don't know Rows: Manual wheelchair Powered wheelchair Walking aids (for example crutches, walking frame) Hearing aids Breathing aid/machine (for example CPAP [continuous positive airway pressure therapy] machine) Home modifications (for example wheelchair ramps and other changes for easy access) Vehicle modifications Modifications at school (for example wheelchair ramps and other changes for easy access) Personal care/support assistance Dental work   - If there are any other things your child/children use (excluding medicines and hospital care), you can use the space below to describe how they are funded | Yes | - | - | x | x | x | - | - | - | - |
| 92 | In the past 4 weeks, how much have you spent out of pocket (using your own money) on the following things? | Indicate the costs for all that apply. If you did not spend money on something, please enter '0'. Give your best estimate if you are not sure. Example: If you have spent £85 on medicines please enter '85'. | Only one answer per row | Columns: Cost/4 weeks in your local currency Rows: Medicines Physiotherapy (including osteopathy, massage therapy) Psychotherapist Travel to medical appointments Personal care/support assistance   - Other (please specify): | Yes | - | - | x | x | x | - | - | - | - |
| 93 | Have you experienced any challenges accessing any of the following things? | Please select all that apply | Yes | Manual wheelchair Powered wheelchair Walking aids (for example crutches, walking frame) Hearing aids Breathing aid/machine (for example CPAP [continuous positive airway pressure therapy] machine) Home modifications (for example wheelchair ramps and other changes for easy access) Vehicle modifications Modifications at school (for example wheelchair ramps and other changes for easy access) Personal care/support assistance Dental work No I don't know Prefer not to say | No | - | - | x | x | x | - | - | - | - |
| 94 | Please indicate how you feel the following statements describe the difficulties you have experienced when trying to access devices and services to manage your child/children's OI. | Please select one answer per row | Only one answer per row | Columns: Strongly agree Agree Neither agree nor disagree Disagree Strongly disagree Not applicable I don't know Prefer not to say Rows: I don't have enough information to access the things they need The things they need are not offered to us by our healthcare provider The things they need are not available to us (for example not available in our country or region) I cannot afford the things they need to manage my OI   - Other (please specify): | Yes | - | - | x | x | x | - | - | - | - |
| 95 | Excluding school and therapy sessions, who are the people that take care of your child/children with OI? | Please select all that apply | Yes | Me My partner Other family members or relatives Friends Caregivers/childminders I pay for Caregivers/childminders provided by social services Caregivers/childminders provided by charities Prefer not to say Other (please specify):   - Other (please specify): | Yes | - | - | x | x | x | - | - | - | - |
| 96 | Please indicate if you feel any of the following aspects of your child's life are negatively impacted by OI. | Please select one answer per row | Only one answer per row | Columns: Severely impacted Moderately impacted Mildly impacted Very mildly impacted Not impacted Not applicable I don't know Prefer not to say  Rows: School attendance or ability to participate in other educational activities Social life (for example, interactions with other children) The type of leisure activities they can do Ability to get on with normal daily tasks of living (for example, bathing, play, eating, dressing) Mental health Happiness Relationships with family   - Other (please specify): | Yes | - | - | x | x | x | - | - | - | - |
| 97 | Please indicate if you feel any of the following aspects of child's 1 life are negatively impacted by OI | Please select one answer per row | Only one answer per row | Columns: Severely impacted Moderately impacted Mildly impacted Very mildly impacted Not impacted Not applicable I don't know Prefer not to say Rows: School attendance or ability to participate in other educational activities Social life (for example, interactions with other children) The type of leisure activities they can do Ability to get on with normal daily tasks of living (for example, bathing, play, eating, dressing) Mental health Happiness Relationships with family   - Other (please specify): | Yes | - | - | x | - | - | - | - | - | - |
| 98 | Please indicate if you feel any of the following aspects of child's 2 life are negatively impacted by OI | Please select one answer per row | Only one answer per row | Columns: Severely impacted Moderately impacted Mildly impacted Very mildly impacted Not impacted Not applicable I don't know Prefer not to say Rows: School attendance or ability to participate in other educational activities Social life (for example, interactions with other children) The type of leisure activities they can do Ability to get on with normal daily tasks of living (for example, bathing, play, eating, dressing) Mental health Happiness Relationships with family   - Other (please specify): | Yes | - | - | - | x | - | - | - | - | - |
| 99 | Please indicate if you feel any of the following aspects of child's 1 life are negatively impacted by OI | Please select one answer per row | Only one answer per row | Columns: Severely impacted Moderately impacted Mildly impacted Very mildly impacted Not impacted Not applicable I don't know Prefer not to say Rows: School attendance or ability to participate in other educational activities Social life (for example, interactions with other children) The type of leisure activities they can do Ability to get on with normal daily tasks of living (for example, bathing, play, eating, dressing) Mental health Happiness Relationships with family  - Other (please specify): | Yes | - | - | - | x | - | - | - | - | - |
| 100 | Please indicate if you feel any of the following aspects of child’s 2 life are negatively impacted by OI | Please select one answer per row | Only one answer per row | Columns: Severely impacted Moderately impacted Mildly impacted Very mildly impacted Not impacted Not applicable I don't know Prefer not to say Rows: School attendance or ability to participate in other educational activities Social life (for example, interactions with other children) The type of leisure activities they can do Ability to get on with normal daily tasks of living (for example, bathing, play, eating, dressing) Mental health Happiness Relationships with family  - Other (please specify): | Yes | - | - | - | - | x | - | - | - | - |
| 101 | Please indicate if you feel any of the following aspects of child’s 3 life are negatively impacted by OI | Please select one answer per row | Only one answer per row | Columns: Severely impacted Moderately impacted Mildly impacted Very mildly impacted Not impacted Not applicable I don't know Prefer not to say Rows: School attendance or ability to participate in other educational activities Social life (for example, interactions with other children) The type of leisure activities they can do Ability to get on with normal daily tasks of living (for example, bathing, play, eating, dressing) Mental health Happiness Relationships with family  - Other (please specify): | Yes | - | - | - | - | x | - | - | - | - |
| 102 | In the past 12 months, how would you describe the impact that OI has had on your life? | This question is about understanding the 'negative' impacts or challenges you have faced. We will ask you about any positive impacts later in the survey, Please select one answer per row. | Only one answer per row | Columns: Severely impacted Moderately impacted Mildly impacted Very mildly impacted Not impacted I don't know Prefer not to say Rows: The number of hours you work The type of job you can do Your career choices Your ability to care for yourself (for example, dressing, cooking, bathing) Your ability to live independently The type of leisure activities you can do (for example, hobbies, sports, reading) Your social life (socialising with friends, attending events, participating in activities) Your relationships with family and friends Your romantic relationships Your sexual health Your mental health Your happiness  - Are there any other aspects of your life that you feel are impacted by OI? | Yes | - | - | - | - | x | - | - | - | - |
| 103 | Do you feel worried or concerned about any of the following things? | Please select one answer per row | Only one answer per row | Columns: Worry a lot Worry a little Don't worry Not applicable I don't know  Prefer not to say Rows: How getting older will impact your life Not being able to care for yourself in the future Losing your independence Losing your mobility Complications because of your OI Fractures Losing your job Your future financial situation How OI impacts your relationships with family and friends How OI impacts your romantic relationships The future of your child/children with OI  - If you have other worries or concerns, please list them here: | Yes | - | - | - | - | - | x | x | x | - |
| 104 | Do you feel worried or concerned about any of the following things? | Please select one answer per row | Only one answer per row | Columns: Worry a lot Worry a little Don't worry Not applicable I don't know  Prefer not to say  Rows: Not having access to doctors who understand OI Not having access to medicines to manage you or your child/children's OI Not having access to care to manage you or your child/children's OI Side effects of the treatment you or child/children are receiving Having financial means to pay for you or your child/children's (with OI) medical treatment and care | No | - | - | - | - | - | x | x | x | - |
| 105 | Do you feel worried or concerned about any of the following things? | Please select one answer per row | Only one answer per row | Columns: Worry a lot Worry a little Don't worry Not applicable I don't know  Prefer not to say  Rows: Pregnancy Menopause | No | - | - | - | - | - | x | x | x | - |
| 106 | In the past 12 months, how would you describe the impact that OI has had on your life? | This question is about understanding the 'negative' impacts or challenges you have faced. We will ask you about any positive impacts later in the survey. Please select one answer per row. | Only one answer per row | Columns: Severely impacted Moderately impacted Mildly impacted Very mildly impacted Not impacted I don't know Prefer not to say  Rows: The number of hours you work The type of job you can do Your career choices Your ability to care for yourself (for example, dressing, cooking, bathing) Your ability to live independently The type of leisure activities you can do (for example, hobbies, sports, reading) Your social life (socialising with friends, attending events, participating in activities) Your relationships with family and friends Your romantic relationships Your sexual health Your mental health Your happiness   - Are there any other aspects of your life that you feel are impacted by OI? | Yes | x | - | - | - | - | x | x | x | - |
| 107 | Do you feel worried or concerned about any of the following things? | Please select one answer per row | No | Columns:  Worry a lot  Worry a little  Don't worry  Not applicable  I don't know  Prefer not to say  Rows:  How getting older will impact your health  Not being able to care for yourself in the future  Living independently  Losing your independence  Losing your mobility  Being able to have a family of your own  Complications because of your OI  Fractures  Losing your job  Your future financial situation  How OI impacts your relationships with family and friends  How OI impacts your romantic relationships  - If you have other worries or concerns, please list them here | Yes | x | - | - | - | - | - | - | - | - |
| 108 | Do you feel worried or concerned about any of the following things? | Please select one answer per row | No | Columns: Worry a lot Worry a little Don't worry Not applicable I don't know Prefer not to say  Rows: Not having access to doctors who understand OI  Not having access to medicines to manage your OI  Not having access to care to manage your OI  Side effects of the treatment you are receiving  Having financial means to pay for your medical treatment and care | No | x | - | - | - | - | - | - | - | - |
| 109 | Do you feel worried or concerned about any of the following things? | Please select one answer per row | No | Columns: Worry a lot Worry a little Don't worry Not applicable I don't know Prefer not to say  Rows: Pregnancy Menopause | No | x | - | - | - | - | - | - | - | - |
| 110 | At what age (years) were you diagnosed with OI? | If you are not sure, please give your best estimate. | No | I don't know Prenatally At birth <1 Integer (1 to 100) | No | x | - | - | - | - | - | - | - | - |
| 111 | Do any of the following apply to you? | Please select all that apply | Yes | I was initially diagnosed with a different condition(s) My OI was initially suspected to be child abuse None of the above Prefer not to say | No | x | - | - | - | - | x | x | x | - |
| 112 | Which condition(s) where you initially diagnosed with? | Please write the name of the condition you were diagnosed with. If you don't know, please write 'I don't know'. | No | Free text | Yes | x | - | - | - | - | x | x | x | - |
| 113 | Over the past 12 months, have you experienced any of the following signs, symptoms, or events? | Please select all that apply | Yes | Pain Fractures Fatigue Scoliosis or other bone problems Soft tissue problems or injuries (muscles, tendons, ligaments) Hypermobility Joint problems (dislocations, osteoarthritis) Hearing problems Eye or vision problems Dental problems Lung or breathing problems Stomach and bowel problems (for example, constipation, diarrhoea) Kidney and bladder problems (for example, kidney stones, urinary tract infection, incontinence) High blood pressure Heart problems (for example, atrial fibrillation/flutter, valve regurgitation, heart attack, arrhythmia) Sleep disturbance Sexual problems Depression, anxiety, or other mental health problems Basilar invagination (which may include pain in the back of the head or upper neck, trouble talking or swallowing, confusion, feeling dizzy or lightheaded) Gynaecological problems/menstruation problems Fertility/reproductive problems Problems with chewing, swallowing, and speaking Obesity/overweight Low/underweight None of the above Prefer not to say  - Other signs and symptoms you have experienced | Yes | x | - | - | - | - | x | x | x | - |
| 114 | In the past 12 months, how has pain impacted your life? | This question is referring to pain of any type. Please select only one answer. | No | Severely Moderately Mildly Very mildly Not at all I don't know Prefer not to say | No | x | - | - | - | - | x | x | x | - |
| 115 | How would you describe the type and frequency of pain you have experienced in the past 12 months? | Please include all types of pain you experience and select the frequency of each type from the dropdown menus. If you do not experience a type/severity of pain you may leave that field blank. | Yes - several drop down menus per row Severely Moderately Mildly Very mildly Not at all I don't know Prefer not to say | Columns: Mild   Every day Often Sometimes Rarely Never Moderate  Every day Often Sometimes Rarely Never Severe  Every day Often Sometimes Rarely Never Rows: Pain all over your body from nowhere specific Sharp, stabbing, shooting, electric shocks that radiate out Dull, aching, throbbing, squeezing deep inside (from internal organ or non-specific areas) Dull, aching, throbbing in your joints, bones, or muscles Burning, stinging, numbness, tingling, pricking from a specific area   - Other (please specify): | Yes | x | - | - | - | - | x | x | x | - |
| 116 | In the past 12 months, how have fractures impacted your life? | Please select only one answer | No | Severely Moderately Mildly Very mildly Not at all I don't know Prefer not to say | No | x | - | - | - | - | x | x | x | - |
| 117 | In the past 12 months, how many times have you experienced any of the following events? | Please use the dropdown menu to indicate the number of times you have experienced each event. If you have not experienced the event in the past 12 months, please select '0'. | Only one answer per row | Columns: Number of times  Prefer not to say  Integer (0 to 50)  >50  Rows: Arm fractures Leg fractures Vertebral fractures Rib fractures Other fractures (for example, pelvis, skull, fingers, toes, sternum; please specify below)   - Please specify which other fractures you have experienced | Yes | x | - | - | - | - | x | x | x | - |
| 118 | In the past 12 months, how has fatigue impacted your life? | Please select only one answer | No | Severely Moderately Mildly Very mildly Not at all I don't know Prefer not to say | No | x | - | - | - | - | x | x | x | - |
| 119 | In the past 12 months, how have scoliosis/other bone problems impacted your life? | Please select only one answer | No | Severely Moderately Mildly Very mildly Not at all I don't know Prefer not to say | No | x | - | - | - | - | x | x | x | - |
| 120 | In the past 12 months, how have soft tissue (muscles, tendons, ligaments) problems impacted your life? | Please select only one answer | No | Severely Moderately Mildly Very mildly Not at all I don't know Prefer not to say | No | x | - | - | - | - | x | x | x | - |
| 121 | In the past 12 months, how many times have you experienced soft tissue injuries (muscles, tendons, ligaments)? | Please use the dropdown menu to indicate the number of times you have experienced soft tissue injuries. If you have not experienced the event in the past 12 months, please select '0'. | No | Integer (0-50) >50 I don't know Prefer not to say | No | x | - | - | - | - | x | x | x | - |
| 122 | In the past 12 months, how has hypermobility impacted your life? | Please select only one answer | No | Severely Moderately Mildly Very mildly Not at all I don't know Prefer not to say | No | x | - | - | - | - | x | x | x | - |
| 123 | In the past 12 months, how have joint problems (for example dislocations or osteoarthritis) impacted your life? | Please select only one answer | No | Severely Moderately Mildly Very mildly Not at all I don't know Prefer not to say | No | x | - | - | - | - | x | x | x | - |
| 124 | In the past 12 months, how have hearing problems impacted your life? | Please select only one answer | No | Severely Moderately Mildly Very mildly Not at all I don't know Prefer not to say | No | x | - | - | - | - | x | x | x | - |
| 125 | In the past 12 months, how have eye or vision problems impacted your life? | Please select only one answer | No | Severely Moderately Mildly Very mildly Not at all I don't know Prefer not to say | No | x | - | - | - | - | x | x | x | - |
| 126 | In the past 12 months, how have dental problems impacted your life? | Please select only one answer | No | Severely Moderately Mildly Very mildly Not at all I don't know Prefer not to say | No | x | - | - | - | - | x | x | x | - |
| 127 | In the past 12 months, how have lung or breathing problems impacted your life? | Please select only one answer | No | Severely Moderately Mildly Very mildly Not at all I don't know Prefer not to say | No | x | - | - | - | - | x | x | x | - |
| 128 | In the past 12 months, how have stomach and bowel problems (for example constipation or diarrhoea) impacted your life? | Please select only one answer | No | Severely Moderately Mildly Very mildly Not at all I don't know Prefer not to say | No | x | - | - | - | - | x | x | x | - |
| 129 | In the past 12 months, how have kidney and bladder problems impacted your life? | Please select only one answer | No | Severely Moderately Mildly Very mildly Not at all I don't know Prefer not to say | No | x | - | - | - | - | x | x | x | - |
| 130 | In the past 12 months, how has high blood pressure impacted your life? | Please select only one answer | No | Severely Moderately Mildly Very mildly Not at all I don't know Prefer not to say | No | x | - | - | - | - | x | x | x | - |
| 131 | In the past 12 months, how have heart events (for example heart attacks, arrhythmia) impacted your life? | Please select only one answer | No | Severely Moderately Mildly Very mildly Not at all I don't know Prefer not to say | No | x | - | - | - | - | x | x | x | - |
| 132 | In the past 12 months, how many times have you experienced cardiac events (such as heart attacks, arrhythmia)? | Please use the dropdown menu to indicate the number of times you have experienced cardiac events. If you have not experienced cardiac event in the past 12 months, please select '0'. | No | Integer (0-50) >50 I don't know Prefer not to say | No | x | - | - | - | - | x | x | x | - |
| 133 | In the past 12 months, how has sleep disturbance impacted your life? | Please select only one answer | No | Severely Moderately Mildly Very mildly Not at all I don't know Prefer not to say | No | x | - | - | - | - | x | x | x | - |
| 134 | In the past 12 months, how have sexual problems impacted your life? | Please select only one answer | No | Severely Moderately Mildly Very mildly Not at all I don't know Prefer not to say | No | x | - | - | - | - | x | x | x | - |
| 135 | In the past 12 months, how have depression/anxiety or other mental health problems impacted your life? | Please select only one answer | No | Severely Moderately Mildly Very mildly Not at all I don't know Prefer not to say | No | x | - | - | - | - | x | x | x | - |
| 136 | In the past 12 months, how has basilar invagination (which may include pain in the back of the head or upper neck, trouble talking or swallowing, confusion, feeling dizzy or lightheaded) impacted your life? | Select only one answer | No | Severely Moderately Mildly Very mildly Not at all I don't know Prefer not to say | No | x | - | - | - | - | x | x | x | - |
| 137 | In the past 12 months, how have gynaecological problems/menstruation problems impacted your life? | Please select only one answer | No | Severely Moderately Mildly Very mildly Not at all I don't know Prefer not to say | No | x | - | - | - | - | x | x | x | - |
| 138 | In the past 12 months, how have fertility/reproductive problems impacted your life? | Please select only one answer | No | Severely Moderately Mildly Very mildly Not at all I don't know Prefer not to say | No | x | - | - | - | - | x | x | x | - |
| 139 | In the past 12 months, how have problems with chewing, swallowing, and speaking impacted your life? | Please select only one answer | No | Severely Moderately Mildly Very mildly Not at all I don't know Prefer not to say | No | x | - | - | - | - | x | x | x | - |
| 140 | In the past 12 months, how have obesity/overweight problems impacted your life? | Please select only one answer | No | Severely Moderately Mildly Very mildly Not at all I don't know Prefer not to say | No | x | - | - | - | - | x | x | x | - |
| 141 | In the past 12 months, how have problems with low/underweight impacted your life? | Please select only one answer | No | Severely Moderately Mildly Very mildly Not at all I don't know Prefer not to say | No | x | - | - | - | - | x | x | x | - |
| 142 | In the past 12 months, how have mobility challenges impacted your life? | Please select only one answer | No | Severely Moderately Mildly Very mildly Not at all I don't know Prefer not to say | No | x | - | - | - | - | x | x | x | - |
| 143 | Prior to the past 12 months (during your lifetime) have you experienced any of the following signs, symptoms, or events? | Please select all that apply | Yes | Pain Fractures Fatigue Scoliosis or other bone problems Soft tissue problems or injuries (muscles, tendons, ligaments) Hypermobility Joint problems (dislocations, osteoarthritis) Hearing problems Eye or vision problems Dental problems Lung or breathing problems Stomach and bowel problems (for example, constipation, diarrhoea) Kidney and bladder problems (for example, kidney stones, urinary tract infection, incontinence) High blood pressure Heart problems (for example, atrial fibrillation/flutter, valve regurgitation, heart attack, arrhythmia) Sleep disturbance Sexual problems Depression, anxiety, or other mental health problems Basilar invagination (which may include pain in the back of the head or upper neck, trouble talking or swallowing, confusion, feeling dizzy or lightheaded) Gynaecological problems/menstruation problems Fertility/reproductive problems Problems with chewing, swallowing, and speaking Obesity/overweight Low/underweight None of the above Prefer not to say   - Other signs and symptoms you have experienced | Yes | x | - | - | - | - | x | x | x | - |
| 144 | In the past 12 months, how many times have you visited hospital? | This includes both planned and emergency visits for any reason, including OI-unrelated reasons. If you are not sure, please give your best estimate. | No | Prefer not to say Integer (0 to 100) | No | x | - | - | - | - | x | x | x | - |
| 145 | Of these times (in the past 12 months), how many times have you visited the emergency department? | Please include all visits, for any reason, including OI-unrelated visits. If you are not sure, please give your best estimate. | No | Prefer not to say Integer (0 to 100) | No | x | - | - | - | - | x | x | x | - |
| 146 | In the past 12 months, how many nights did you spend in hospital overall (for both planned and emergency visits)? | Please include all visits, for any reason, including OI-unrelated visits. If you are not sure, please give your best estimate. | No | Prefer not to say Integer (0 to 100) | No | x | - | - | - | - | x | x | x | - |
| 147 | In the past 12 months, how many nights did you spend in a rehabilitation facility? | Please include all visits, for any reason, including OI-unrelated visits. If you are not sure, please give your best estimate. | No | Not applicable Prefer not to say Integer (0 to 100) | No | x | - | - | - | - | x | x | x | - |
| 148 | In the past 12 months, how many times have your received the following tests and examinations? | Please include any tests and examinations, including OI-unrelated procedures. If you are not sure, please give your best estimate. If you have not received any of these tests in the past 12 months, please select '0'. | Only one answer per row | Columns: Number of times  I don't know Prefer not to say Integer (0 to 49) >50 Rows: Blood test Urine test X-ray CT scan (computed tomography) DEXA scan (dual energy X-ray absorptiometry) MRI scan (magnetic resonance imaging) Ultrasound scan Echo scan (echocardiogram, heart scan) Audiology (hearing) test Other (please specify)  - Please specify which other tests you have received | Yes | x | - | - | - | - | x | x | x | - |
| 149 | Please indicate how often you have visited the following healthcare professionals in the past 12 months. | Please include all visits, for any reason, including OI-unrelated visits. If you are not sure, please give your best estimate. Please also include appointments that took place over the phone or video chat. Please leave blank if you have not seen a healthcare professional. | Only one answer per row | Columns: More than twice weekly Twice weekly Weekly Every other week Monthly Every six weeks Every other month Once per quarter Once every six months Once per year  Rows: General practitioner/family doctor Nurse practitioner/care coordinator Paediatrician (children's doctor) Orthopaedic surgeon/orthopaedist (bone specialist) Nutritionist Psychotherapist/counsellor Physiotherapist Dentist/orthodontist Audiology (hearing) Ophthalmologist (eye) Gynaecologist/obstetrician (women's reproductive health) Endocrinologist (hormones, bone turnover/density) Cardiologist (heart) Neurologist (brain and nervous system) Gastroenterologist (digestive system) Rheumatologist (musculoskeletal system) Pulmonologist (lung/respiratory system) Rehabilitation therapist/doctor Occupational therapist (helps to recover, improve, and maintain skills needed for daily living and working)   - If you have visited any other healthcare professionals, please specify below: | Yes | x | - | - | - | - | x | x | x | - |
| 150 | Have you visited any of the following healthcare professionals in the past (prior to the past 12 months)? | Please include all visits, for any reason, including OI-unrelated visits. Please select all that apply | Yes | General practitioner/family doctor Nurse practitioner/care coordinator Paediatrician (children's doctor) Orthopaedic surgeon/orthopaedist (bone specialist) Nutritionist Psychotherapist/counsellor Physiotherapist Dentist/orthodontist Audiology (hearing) Ophthalmologist (eye) Gynaecologist/obstetrician (women's reproductive health) Endocrinologist (hormones, bone turnover/density) Cardiologist (heart) Neurologist (brain and nervous system) Gastroenterologist (digestive system) Rheumatologist (musculoskeletal system) Pulmonologist (lung/respiratory system) Rehabilitation therapist/doctor Occupational therapist (helps to recover, improve, and maintain skills needed for daily living and working) None of the above Other healthcare professionals you have visited in the past (please specify below)   - Other healthcare professionals you have visited in the past (please specify below) | Yes | x | - | - | - | - | x | x | x | - |
| 151 | Do you use pain medication regularly? | Please select only one answer | No | Yes No Prefer not to say | No | x | - | - | - | - | x | x | x | - |
| 152 | Over the past 4 weeks, how often (on average) have you used pain medication? | If you are not sure, give your best estimate. | No | Daily Several times per week Once per week Less than once per week I haven't used painkillers in the last 4 weeks I don't know Prefer not to say | No | x | - | - | - | - | x | x | x | - |
| 153 | What kinds of other OI treatments are you taking now, or have you taken before? | If you take other treatments not listed below, please specify them in the comments box | Only one answer per row | Columns: I currently take this treatment (in the past 12 months) I used to take this treatment (prior to the past 12 months) I have never taken this treatment  I don't know Prefer not to say  Rows: Bisphosphonates Vitamin D supplements Calcium supplements Muscle relaxants Parathyroid hormone (PTH) (for example, abaloparatide [Tymlos®], teriparatide [Forteo®]) Oestrogen hormone (for example, raloxifene [Evista®], tamoxifen [Nolvadex®]) Antibodies (for example, denosumab [Prolia®], romosozumab [Evenity®], setrusumab)   - Other treatments (please specify): | Yes | x | - | - | - | - | x | x | x | - |
| 154 | What type of bisphosphonates do you currently take? |  | No | Pamidronate (Aredia®) Alendronate (Fosamax®, Fosavance®) Zoledronate (Zometa®, Reclast®, Aclasta®) Risedronate (Actonel®, Atelvia®, Benet®, Ribastamin®) Ibandronate (Boniva®, Bonviva®, Bondronat®, Lasibon®, Quodixor®) Neridronate (Nerixia®, Attilia®) Clodronate (Bonefos®, Clasteon®, Loron®, Sindronat®, Lodronat®) Etidronate (Didronel®) Other (please specify in the comment box) I don't know Prefer not to say   - Other (please specify) | Yes | x | - | - | - | - | x | x | x | - |
| 155 | Following bisphosphonate treatment, how do you feel things have changed for you? | Please select one answer per row | Only one answer per row | Columns: Improved Stayed the same Got worse Not applicable I don't know Prefer not to say  Rows: Growth rate Mobility Fatigue Pain How easily you fracture Bone mass   - Other (please specify): | Yes | x | - | - | - | - | x | x | x | - |
| 156 | Following bisphosphonate treatment, have you experienced any of the following? | Please select all that apply | Yes | Osteonecrosis of the jaw (which may include symptoms like delayed healing, exposed bone, swelling) Sudden hearing loss Stomach problems I have not experienced any of the listed issues Prefer not to say Other (please specify)   - Other (please specify): | Yes | x | - | - | - | - | x | x | x | - |
| 157 | In your life, how many surgeries have you had for the following things? | Please include all surgeries, including those which were not related to your OI. If you are not sure, please give your best estimate. For those that do not apply, please select '0'. | Only one answer per row | Columns: Number of surgeries  I don't know Prefer not to say Integer (0 to 50)  Rows: Rodding Fracture repairs Spine Hearing Teeth Heart Basilar invagination (skull/neck) Soft tissue (for example, tendons, hypermobility, dislocations) Other (please specify below)   - Please specify which other surgeries you have had. | Yes | x | - | - | - | - | x | x | x | - |
| 158 | Overall, how do you feel about the following aspects of your OI treatment and care? | Please select one answer per row | Only one answer per row | Columns: Strongly agree Agree Neither agree nor disagree Disagree Strongly disagree Not applicable I don't know Prefer not to say  Rows: Doctors and other healthcare professionals understand your OI You are provided with sufficient information about your OI You have access to the treatment and care that you need You have access to regular follow up Treatments and treatment options are explained to you sufficiently Doctors and other healthcare professionals understand your needs and concerns Your care is continuous You feel you need to coordinate your own care You feel/felt supported in your transition from paediatric to adult care | No | x | - | - | - | - | x | x | x | - |
| 159 | Please indicate whether you agree with the following statements about how your healthcare might have changed since the start of the COVID-19 pandemic. | Please select one answer per row | Only one answer per row | Columns: Strongly agree Agree Neither agree nor disagree Disagree Strongly disagree Not applicable I don't know Prefer not to say Rows: You have had fewer visits with healthcare providers You have had fewer medical tests and examinations Appointments that previously took place in person are now online/on the phone You have been able to access additional healthcare because more doctors offer phone/online appointments You have avoided seeking medical care because you are worried about exposure to COVID-19 You have avoided visiting the emergency department because of COVID-19 | No | x | - | - | - | - | x | x | x | - |
| 160 | Do you sometimes avoid seeking medical care for reasons other than the COVID-19 pandemic? | Please select only one answer | No | Yes No I don't know Prefer not to say | No | x | - | - | - | - | x | x | x | - |
| 161 | Please indicate whether you avoid seeking medical care due to any of the reasons below: | Please select one answer per row | Only one answer per row | Columns: Strongly agree Agree Neither agree nor disagree Disagree Strongly disagree Not applicable I don't know Prefer not to say Rows: High costs Because of past trauma or negative experiences You can do it better yourself Your healthcare provider is too far away from your home You don't have transportation to access your healthcare provider easily Fear Healthcare professionals in your area are not familiar with OI You don't trust healthcare professionals  - Other reasons: | Yes | x | - | - | - | - | x | x | x | - |
| 162 | In the past 4 weeks, how many days of work have you missed due to your OI or your care responsibilities for your child/children with OI? | None | No | 0 <1 Integer (1 to 28) I don't know Prefer not to say | No | - | - | - | - | - | x | x | x | - |
| 163 | Please indicate your local currency | None | No | AED, (United Arab Emirates dirham) - ZMW, (Zambian kwacha) **(136 currencies)** | No | - | - | - | - | - | x | x | x | - |
| 164 | Are you covered by private health insurance? | Please select only one answer | No | Yes No I don't know Prefer not to say | No | - | - | - | - | - | x | x | x | - |
| 165 | Are your child/children (with OI) covered by private health insurance? | Please select only one answer | No | Yes No I don't know Prefer not to say | No | - | - | - | - | - | x | x | x | - |
| 166 | Why are you not covered by private health insurance? | Please select all that apply | Yes | I don't want private health insurance I don't need private health insurance I can't afford private health insurance Private health insurance is not an option where I live I don't qualify for private health insurance because of my OI Prefer not to say I don't know Other reasons  - other reasons: | Yes | - | - | - | - | - | x | x | x | - |
| 167 | Why is your child/children (with OI) not covered by private health insurance? | Please select all that apply | Yes | We don't want private health insurance We don't need private health insurance We can't afford private health insurance Private health insurance is not an option where we live We don't qualify for private health insurance because of their OI Prefer not to say I don't know Other reasons  - Other reasons: | Yes | - | - | - | - | - | x | x | x | - |
| 168 | How are your healthcare costs covered? | Please select all that apply | Multiple answers per row | Columns: You Your child/children (with OI) Rows: I pay for healthcare myself (out of pocket) Public statutory insurance (for example TK or AOK in Germany) Private health insurance (for example United Healthcare in the US or Bupa in the UK) National healthcare provision (for example Medicaid, CHIP, Canadian Medicare or NHS) Charity funding Financial support from family and friends Employer Other public funding (for example disability benefits, welfare, social security) | No | - | - | - | - | - | x | x | x | - |
| 169 | Do you or your child/children with OI require any of the following? | Please select all that apply | Multiple answers per row | Columns: You Your child/children (with OI) Rows: Manual wheelchair Powered wheelchair Walking aids (for example crutches, walking frame) Hearing aids Breathing aid/machine (for example CPAP [continuous positive airway pressure therapy] machine) Home modifications (for example wheelchair ramps and other changes for easy access) Vehicle modifications Modifications at work/school (for example wheel chair ramps and other changes for easy access) Personal care/support assistance Dental work  - If there are any other things you or your child/children with OI use (excluding medicines and hospital care), you can use the space below to describe how they are funded | No | - | - | - | - | - | x | x | x | - |
| 170 | In the past 4 weeks, how much have you spent out of pocket (using your own money) on the following things for yourself and your child/children (with OI)? | Indicate the costs for all that apply. If you did not spend money on something please enter '0'. Give your best estimate if you are not sure. Example: If you have spent £85 on medicines please enter '85'. | Only one answer per row | Columns: Cost/4 weeks in your local currency  Rows: Medicines Physiotherapy (including osteopathy, massage therapy) Psychotherapist Travel to medical appointments Personal care/support assistance   - Other (please specify): | Yes | - | - | - | - | - | x | x | x | - |
| 171 | Have you or your child/children (with OI) experienced any challenges accessing any of the following things? | Please select all that apply | Yes | Manual wheelchair Powered wheelchair Walking aids (for example crutches, walking frame) Hearing aids Breathing aid/machine (for example CPAP [continuous positive airway pressure therapy] machine) Home modifications (for example wheelchair ramps and other changes for easy access) Vehicle modifications Modifications at work (for example wheel chair ramps and other changes for easy access) Personal care/support assistance Dental work No I don't know Prefer not to say | No | - | - | - | - | - | x | x | x | - |
| 172 | Please indicate how you feel the following statements describe the difficulties you or your child/children (with OI) have experienced when trying to access devices and services to manage your OI. | Please select one answer per row | Only one answer per row | Columns: Strongly agree Agree Neither agree nor disagree Disagree Strongly disagree Not applicable I don't know Prefer not to say Rows: I don't have enough information to access the things we need The things we need are not offered to us by our healthcare provider The things we need are not available to us (for example not available in our country or region) I cannot afford the things we need to manage our OI   - Other (please specify): | Yes | - | - | - | - | - | x | x | x | - |
| 173 | In the past 4 weeks, how many days of work have you missed because of your OI? |  | No | 0 less than 1 Integer (1 to 28) I don't know Prefer not to say | No | x | - | - | - | - | x | x | x | - |
| 174 | Please indicate your local currency |  | No | AED, (United Arab Emirates dirham) - ZMW, (Zambian kwacha) (136 currencies) | No | x | - | - | - | - | - | - | - | - |
| 175 | Are you covered by private health insurance? | Please select only one answer | No | Yes No I don't know Prefer not to say | No | x | - | - | - | - | - | - | - | - |
| 176 | Why are you not covered by private health insurance? | Please select all that apply | Yes | I don't want private health insurance I don't need private health insurance I can't afford private health insurance Private health insurance is not an option where I live I don't qualify for private health insurance because of my OI Prefer not to say I don't know Other reasons   - other reasons: | Yes | x | - | - | - | - | - | - | - | - |
| 177 | How are your healthcare costs covered? | Please select all that apply | Yes | Public statutory insurance (for example TK or AOK in Germany) Private health insurance (for example United Healthcare in the US or Bupa in the UK) National healthcare provision (for example Medicaid, CHIP, Canadian Medicare or NHS) Charity funding I pay for healthcare myself (out of pocket) Financial support from family and friends Employer Other public funding (for example disability benefits, welfare, social security) Other funding (please specify):   - other funding (please specify): | Yes | x | - | - | - | - | - | - | - | - |
| 178 | If you use any of the following things, who covers the cost? | Please select all that apply for each item you use. If you do not use an item please select 'Not applicable'. | Multiple answers per row | Columns: Not applicable I pay myself Private health insurance Public statutory insurance Charity funding Family/friends National healthcare provision Employer Other public funding I don't know  Rows: Manual wheelchair Powered wheelchair Walking aids (for example crutches, walking frame) Hearing aids Breathing aid/machine (for example CPAP [continuous positive airway pressure therapy] machine) Home modifications (for example wheelchair ramps and other changes for easy access) Vehicle modifications Modifications at work (for example wheelchair ramps and other changes for easy access) Personal care/support assistance Dental work   - If there are any other things you use (excluding medicines and hospital care), you can use the space below to describe how they are funded. | Yes | x | - | - | - | - | - | - | - | - |
| 179 | In the past 4 weeks, how much have you spent out of pocket (using your own money) on the following things? | Indicate the costs for all that apply. If you did not spend money on something, please enter '0'. Give your best estimate if you are not sure. Example: If you have spent £85 on medicines please enter '85'. | Only one answer per row | Columns: Cost/4 weeks in your local currency  Rows: Medicines Physiotherapy (including osteopathy, massage therapy) Psychotherapist Travel to medical appointments Personal care/support assistance Other (please specify below)   - Other (please specify): | Yes | x | - | - | - | - | - | - | - | - |
| 180 | Have you experienced any challenges accessing any of the following things? | Please select all that apply | Yes | Manual wheelchair Powered wheelchair Walking aids (for example crutches, walking frame) Hearing aids Breathing aid/machine (for example CPAP [continuous positive airway pressure therapy] machine) Home modifications (for example wheelchair ramps and other changes for easy access) Vehicle modifications Modifications at work (for example wheelchair ramps and other changes for easy access) Personal care/support assistance Dental work No I don't know Prefer not to say | No | x | - | - | - | - | - | - | - | - |
| 181 | Please indicate how you feel the following statements describe the difficulties you have experienced when trying to access devices and services to manage your OI. | Please select one answer per row | Only one answer per row | Columns: Strongly agree Agree Neither agree nor disagree Disagree Strongly disagree Not applicable I don't know Prefer not to say  Rows: I don't have enough information to access the things I need The things I need are not offered to me by my healthcare provider The things I need are not available to me (for example not available in my country or region) I cannot afford the things I need to manage my OI   - Other (please specify): | Yes | x | - | - | - | - | - | - | - | - |
| 182 | At what age (years) was your child diagnosed with OI? | If you are not sure, please give your best estimate | No | Prenatally At birth ˂1 Integer (1 to 18) I don't know Prefer not to say | No | - | - | - | - | - | - | - | - | - |
| 183 | Do any of the following apply to you? | Please select all that apply | Yes | My child was initially diagnosed with a different condition(s) My child's OI was initially suspected as child abuse None of the above Prefer not to say | No | - | - | x | - | - | - | - | - | - |
| 184 | Which condition(s) was your child initially diagnosed with? | Please write the name of the condition your child was diagnosed with. If you don't know, please write 'I don't know'. | Free text |  | Yes | - | - | x | - | - | - | - | - | - |
| 185 | How long did it take from your child's first fracture to receiving an OI diagnosis? | None | No | My child received their diagnosis before their first fracture My child received their diagnosis straight away <3 months 3-6 months 7-12 months 13-24 months 2-5 years 6-10 years More than 10 years I'm not sure Prefer not to say | No | - | - | x | - | - | - | - | - | - |
| 186 | How has diagnosis impacted your child's journey through the healthcare system? | Please select one answer per row | Only one answer per row | Columns: Strongly agree Agree Neither agree nor disagree Disagree Strongly disagree Not applicable I don't know Prefer not to say Rows: I am satisfied with the length of time taken for my child's OI diagnosis I am satisfied with the diagnostic services that were available to my child My child has received more tailored support and care after their diagnosis My child has had better access to specialist OI services after their diagnosis I feel I am better able to manage my child's condition after their diagnosis | No | - | - | x | - | - | - | - | - | - |
| 187 | Over the past 12 months, has your child experienced any of the following signs, symptoms, or events? | Please select all that apply | Yes | Pain Fractures Fatigue Scoliosis or other bone problems Soft tissue problems or injuries (muscles, tendons, ligaments) Hypermobility Joint problems (dislocations, osteoarthritis) Hearing problems Eye or vision problems Dental problems Lung or breathing problems Stomach and bowel problems (for example, constipation, diarrhoea) Kidney and bladder problems (for example, kidney stones, urinary tract infection, incontinence) High blood pressure Heart problems (for example, atrial fibrillation/flutter, valve regurgitation, heart attack, arrhythmia) Sleep disturbance Depression, anxiety, or other mental health problems Basilar invagination (which may include pain in the back of the head or upper neck, trouble talking or swallowing, confusion, feeling dizzy or lightheaded) Gynaecological problems/menstruation problems Problems with chewing, swallowing, and speaking Obesity/overweight Low/underweight None of the above Prefer not to say   - Other signs and symptoms your child has experienced | Yes | - | - | x | - | - | - | - | - | - |
| 188 | In the past 12 months, how has pain impacted your child's life? | This question is referring to pain of any type. Please select only one answer. | No | Severely impacted Moderately impacted Mildly impacted Very mildly impacted Not impacted Not applicable I don't know Prefer not to say | No | - | - | x | - | - | - | - | - | - |
| 189 | How would you describe the type and frequency of pain your child has experienced in the past 12 months? | This question is for parents of children who can describe the pain they experience. If your child cannot describe their pain, please leave this question blank, and respond to the next question instead. Please include all types of pain your child experiences and select the frequency of each type from the dropdown menus. If your child does not experience a type/severity of pain you may leave that field blank. | Yes - several drop down menus per row | Columns: Mild  Every day Often Sometimes Rarely Never  Moderate Every day Often Sometimes Rarely Never  Severe Every day Often Sometimes Rarely Never Rows: Pain all over their body from nowhere specific Sharp, stabbing, shooting, electric shocks that radiate out Dull, aching, throbbing, squeezing deep inside (from internal organ or non-specific areas) Dull, aching, throbbing in your joints, bones, or muscles Burning, stinging, numbness, tingling, pricking from a specific area For younger children: How often does your child experience any type of pain  - Other (please specify): | Yes | - | - | x | - | - | - | - | - | - |
| 190 | How would you describe the type and frequency of pain your child has experienced in the past 12 months? | This question is for parents of children who cannot describe the pain they experience. If your child can describe their pain, please respond to the question above and leave this question blank. Please select the frequency of pain from the dropdown menus. If your child does not experience a severity of pain you may leave that field blank. | Yes - several drop down menus per row | Columns: Mild  Every day Often Sometimes Rarely Never  Moderate Every day Often Sometimes Rarely Never  Severe Every day Often Sometimes Rarely Never Rows: How often does your child experience pain? | No | - | - | x | - | - | - | - | - | - |
| 191 | In the past 12 months, how have fractures impacted your child's life? | Please select only one answer | No | Severely impacted Moderately impacted Mildly impacted Very mildly impacted Not impacted Not applicable I don't know Prefer not to say | No | - | - | x | - | - | - | - | - | - |
| 192 | In the past 12 months, how many times has your child experienced any of the following events? | Please use the dropdown menu to indicate the number of times your child has experienced each event. If your child has not experienced the event in the past 12 months, please select '0'. | Only one answer per row | Columns: Number of times  Prefer not to say Integer (0 to 50) >50  Rows: Arm fractures Leg fractures Vertebral fractures Rib fractures Other fractures (for example, pelvis, skull, fingers, toes, sternum; please specify below)   - Please specify which other fractures your child has experienced | Yes | - | - | x | - | - | - | - | - | - |
| 193 | In the past 12 months, how has fatigue impacted your child's life? | Please select only one answer | No | Severely Moderately Mildly Very mildly Not at all I don't know Prefer not to say | No | - | - | x | - | - | - | - | - | - |
| 194 | In the past 12 months, how have scoliosis/other bone problems impacted your child's life? | Please select only one answer | No | Severely Moderately Mildly Very mildly Not at all I don't know Prefer not to say | No | - | - | x | - | - | - | - | - | - |
| 195 | In the past 12 months, how have soft tissue problems impacted your child's life? | Please select only one answer | No | Severely Moderately Mildly Very mildly Not at all I don't know Prefer not to say | No | - | - | x | - | - | - | - | - | - |
| 196 | In the past 12 months, how many times has your child experienced soft tissue injuries (muscles, tendons, ligaments)? | Please use the dropdown menu to indicate the number of times your child has experienced the event. If your child has not experienced the event in the past 12 months, please select '0'. | No | Prefer not to say Integer (0 to 50) >50 | No | - | - | x | - | - | - | - | - | - |
| 197 | In the past 12 months, how has hypermobility impacted your child's life? | Please select only one answer | No | Severely Moderately Mildly Very mildly Not at all I don't know Prefer not to say | No | - | - | x | - | - | - | - | - | - |
| 198 | In the past 12 months, how have joint problems (for example dislocations or osteoarthritis) impacted your child's life? | Please select only one answer | No | Severely Moderately Mildly Very mildly Not at all I don't know Prefer not to say | No | - | - | x | - | - | - | - | - | - |
| 199 | In the past 12 months, how have hearing problems impacted your child's life? | Please select only one answer | No | Severely Moderately Mildly Very mildly Not at all I don't know Prefer not to say | No | - | - | x | - | - | - | - | - | - |
| 200 | In the past 12 months, how have eye or vision problems impacted your child's life? | Please select only one answer | No | Severely Moderately Mildly Very mildly Not at all I don't know Prefer not to say | No | - | - | x | - | - | - | - | - | - |
| 201 | In the past 12 months, how have dental problems impacted your child's life? | Please select only one answer | No | Severely Moderately Mildly Very mildly Not at all I don't know Prefer not to say | No | - | - | x | - | - | - | - | - | - |
| 202 | In the past 12 months, how have lung or breathing problems impacted your child's life? | Please select only one answer | No | Severely Moderately Mildly Very mildly Not at all I don't know Prefer not to say | No | - | - | x | - | - | - | - | - | - |
| 203 | In the past 12 months, how have stomach and bowel problems (for example constipation or diarrhoea) impacted your child's life? | Please select only one answer | No | Severely Moderately Mildly Very mildly Not at all I don't know Prefer not to say | No | - | - | x | - | - | - | - | - | - |
| 204 | In the past 12 months, how have kidney and bladder problems impacted your child's life? | Please select only one answer | No | Severely Moderately Mildly Very mildly Not at all I don't know Prefer not to say | No | - | - | x | - | - | - | - | - | - |
| 205 | In the past 12 months, how has high blood pressure impacted your child's life? | Please select only one answer | No | Severely Moderately Mildly Very mildly Not at all I don't know Prefer not to say | No | - | - | x | - | - | - | - | - | - |
| 206 | In the past 12 months, how have heart events (for example heart attacks, arrhythmia) impacted your child's life? | Please select only one answer | No | Severely Moderately Mildly Very mildly Not at all I don't know Prefer not to say | No | - | - | x | - | - | - | - | - | - |
| 207 | In the past 12 months, how many times has your child experienced heart events (such as heart attacks, arrhythmia)? | Please use the dropdown menu to indicate the number of times your child has experienced heart events. If your child has not experienced heart events in the past 12 months, please select '0'. | No | Prefer not to say Integer (0 to 50) >50 | No | - | - | x | - | - | - | - | - | - |
| 208 | In the past 12 months, how has sleep disturbance impacted your child's life? | Please select only one answer | No | Severely Moderately Mildly Very mildly Not at all I don't know Prefer not to say | No | - | - | x | - | - | - | - | - | - |
| 209 | In the past 12 months, how have depression/anxiety or other mental health problems impacted your child's life? | Please select only one answer | No | Severely Moderately Mildly Very mildly Not at all I don't know Prefer not to say | No | - | - | x | - | - | - | - | - | - |
| 210 | In the past 12 months, how has basilar invagination (which may include pain in the back of the head or upper neck, trouble talking or swallowing, confusion, feeling dizzy or lightheaded) impacted your child's life? | Select only one answer | No | Severely Moderately Mildly Very mildly Not at all I don't know Prefer not to say | No | - | - | x | - | - | - | - | - | - |
| 211 | In the past 12 months, how have gynaecological problems/menstruation problems impacted your child's life? | Please select only one answer | No | Severely Moderately Mildly Very mildly Not at all I don't know | No | - | - | x | - | - | - | - | - | - |
| 212 | In the past 12 months, how have problems with chewing, swallowing, and speaking impacted your child's life? | Please select only one answer | No | Severely Moderately Mildly Very mildly Not at all I don't know Prefer not to say | No | - | - | x | - | - | - | - | - | - |
| 213 | In the past 12 months, how have obesity/overweight problems impacted your child's life? | Please select only one answer | No | Severely Moderately Mildly Very mildly Not at all I don't know Prefer not to say | No | - | - | x | - | - | - | - | - | - |
| 214 | In the past 12 months, how have problems with low/underweight impacted your child's life? | Please select only one answer | No | Severely Moderately Mildly Very mildly Not at all I don't know Prefer not to say | No | - | - | x | - | - | - | - | - | - |
| 215 | In the past 12 months, how have mobility challenges impacted your child's life? | Please select only one answer | No | Severely Moderately Mildly Very mildly Not at all I don't know Prefer not to say | No | - | - | x | - | - | - | - | - | - |
| 216 | Prior to the past 12 months (throughout their life), has your child experienced any of the following signs, symptoms, or events? | Please select all that apply | Yes | Pain Fractures Fatigue Scoliosis or other bone problems Soft tissue problems or injuries (muscles, tendons, ligaments) Hypermobility Joint problems (dislocations, osteoarthritis) Hearing problems Eye or vision problems Dental problems Lung or breathing problems Stomach and bowel problems (for example, constipation, diarrhoea) Kidney and bladder problems (for example, kidney stones, urinary tract infection, incontinence) High blood pressure Heart problems (for example, atrial fibrillation/flutter, valve regurgitation, heart attack, arrhythmia) Sleep disturbance Depression, anxiety, or other mental health problems Basilar invagination (which may include pain in the back of the head or upper neck, trouble talking or swallowing, confusion, feeling dizzy or lightheaded) Gynaecological problems/menstruation problems Problems with chewing, swallowing, and speaking Obesity/overweight Low/underweight None of the above Prefer not to say   - Other signs and symptoms your child has experienced | Yes | - | - | x | - | - | - | - | - | - |
| 217 | In the past 12 months, how many times has your child visited hospital? | Please include all visits, for any reason, including OI-unrelated visits. This includes both planned and emergency visits. If you are not sure, please give your best estimate. | No | Prefer not to say Integer (0 to 100) | No | - | - | x | - | - | - | - | - | - |
| 218 | Of these times (in the past 12 months), how many times has your child visited an emergency department? | Please include all visits, for any reason, including OI-unrelated visits. If you are not sure, please give your best estimate | No | Prefer not to say Integer (0 to 100) | No | - | - | x | - | - | - | - | - | - |
| 219 | In the past 12 months, how many nights did your child spend in hospital overall (for both planned and emergency visits)? | Please include all visits, for any reason, including OI-unrelated visits. If you are not sure, please give your best estimate | No | Prefer not to say Integer (0 to 100) | No | - | - | x | - | - | - | - | - | - |
| 220 | In the past 12 months, how many nights has your child spent in a rehabilitation facility? | Please include all visits, for any reason, including OI-unrelated visits. If you are not sure, please give your best estimate. | No | Not applicable Prefer not to say Integer (0 to 100) | No | - | - | x | - | - | - | - | - | - |
| 221 | In the past 12 months, how many times has your child received the following tests and examinations? | Please include any tests and examinations, including OI-unrelated procedures. If you are not sure, please give your best estimate. If your child has not received any of these tests in the past 12 months, please select '0'. | Only one answer per row | Columns: Number of times I don't know Prefer not to say Integer (0 to 49) >50 Rows: Blood test Urine test X-ray CT scan (computed tomography) DEXA scan (dual energy X-ray absorptiometry) MRI scan (magnetic resonance imaging) Ultrasound scan Echo scan (echocardiogram, heart scan) Audiology (hearing) test   - Other (please specify) | Yes | - | - | x | - | - | - | - | - | - |
| 222 | Please indicate how often your child has visited the following healthcare professionals in the past 12 months. | Please include all visits, for any reason, including OI-unrelated visits. If you are not sure, please give your best estimate. Please also include appointments that took place over the phone or video chat. If your child has not visited a healthcare professional please leave the field blank. | Only one answer per row | Columns: More than twice weekly Twice weekly Weekly Every other week Monthly Every six weeks Every other month Once per quarter Once every six months Once per year Rows: General practitioner/family doctor Nurse practitioner/care coordinator Paediatrician (children's doctor) Orthopaedic surgeon/orthopaedist (bone specialist) Nutritionist Psychotherapist/counsellor Physiotherapist Dentist/orthodontist Audiology (hearing) Ophthalmologist (eye) Gynaecologist/obstetrician (women's reproductive health) Endocrinologist (hormones, bone turnover/density) Cardiologist (heart) Neurologist (brain and nervous system) Gastroenterologist (digestive system) Rheumatologist (musculoskeletal system) Pulmonologist (lung/respiratory system) Rehabilitation therapist/doctor Occupational therapist (helps to recover, improve, and maintain skills needed for daily living and working)   - If your child has visited any other healthcare professionals, please specify below: | Yes | - | - | x | - | - | - | - | - | - |
| 223 | Has your child visited any of the following healthcare professions in the past (prior to the past 12 months)? | Please include all visits, for any reason, including OI-unrelated visits. Please select all that apply | Yes | General practitioner/family doctor Nurse practitioner/care coordinator Paediatrician (children's doctor) Orthopaedic surgeon/orthopaedist (bone specialist) Nutritionist Psychotherapist/counsellor Physiotherapist Dentist/orthodontist Audiology (hearing) Ophthalmologist (eye) Gynaecologist/obstetrician (women's reproductive health) Endocrinologist (hormones, bone turnover/density) Cardiologist (heart) Neurologist (brain and nervous system) Gastroenterologist (digestive system) Rheumatologist (musculoskeletal system) Pulmonologist (lung/respiratory system) Rehabilitation therapist/doctor Occupational therapist (helps to recover, improve, and maintain skills needed for daily living and working) | Yes | - | - | x | - | - | - | - | - | - |
| 224 | Does your child use pain medication regularly? | Please select only one answer | No | Yes No Prefer not to say | No | - | - | x | - | - | - | - | - | - |
| 225 | Over the past 4 weeks, how often (on average) has your child used pain medication? | If you are not sure, please give your best estimate. | No | Daily Several times per week Once per week Less than once per week I haven't used painkillers in the last 4 weeks I don't know Prefer not to say | No | - | - | x | - | - | - | - | - | - |
| 226 | What kinds of other OI treatments is your child taking now, or have they taken before? | Please select one answer per row. If your child takes other treatments not listed below, please specify them in the comments box | Only one answer per row | Columns: My child takes this treatment (in the past 12 months) My child used to take this treatment (prior to the past 12 months) My child has never taken this treatment  I don't know Prefer not to say Rows: Bisphosphonates Vitamin D supplements Calcium supplements Muscle relaxants Parathyroid hormone (PTH) (for example, abaloparatide [Tymlos®], teriparatide [Forteo®]) Oestrogen hormone (for example, raloxifene [Evista®], tamoxifen [Nolvadex®]) Antibodies (for example, denosumab [Prolia®], romosozumab [Evenity®], setrusumab)   - Other treatments (please specify): | Yes | - | - | x | - | - | - | - | - | - |
| 227 | What type of bisphosphonates does your child currently take? |  | No | Pamidronate (Aredia®) Alendronate (Fosamax®, Fosavance®) Zoledronate (Zometa®, Reclast®, Aclasta®) Risedronate (Actonel®, Atelvia®, Benet®, Ribastamin®) Ibandronate (Boniva®, Bonviva®, Bondronat®, Lasibon®, Quodixor®) Neridronate (Nerixia®, Attilia®) Clodronate (Bonefos®, Clasteon®, Loron®, Sindronat®, Lodronat®) Etidronate (Didronel®)¨Other (please specify in the comment box) I don't know Prefer not to say   - Other (please specify) | Yes | - | - | x | - | - | - | - | - | - |
| 228 | Following bisphosphonate treatment, how do you feel things have changed for your child? | Please select one answer per row | Only one answer per row | Columns: Improved Stayed the same Got worse Not applicable I don't know Prefer not to say Rows: Growth rate Mobility Fatigue Pain How easily they fracture Bone mass  - Other (please specify) | Yes | - | - | x | - | - | - | - | - | - |
| 229 | Following bisphosphonate treatment, has your child experienced any of the following? | Please select all that apply | Yes | Osteonecrosis of the jaw (which may include symptoms like delayed healing, exposed bone, swelling) Sudden hearing loss Stomach problems They have not experienced any of the listed issues Prefer not to say Other (please specify)   - Other (please specify): | Yes | - | - | x | - | - | - | - | - | - |
| 230 | In your child's life, how many surgeries have they had for the following things? | Please include surgeries for any reason, including OI-unrelated reasons. If you are not sure, please give your best estimate. For those that do not apply, please select '0'. | Only one answer per row | Columns: Number of surgeries  I don't know Prefer not to say Integer (0 to 49) >50 Rows: Rodding Fracture repairs Spine Hearing Teeth Heart Tonsils and adenoids Basilar invagination (skull/neck) Soft tissue (for example, tendons, hypermobility, dislocations) Other (please specify below)   - Other (please specify) | Yes | - | - | x | - | - | - | - | - | - |
| 231 | Overall, how do you feel about the following aspects of your child's OI treatment and care? | Please select one answer per row | Only one answer per row | Columns: Strongly agree Agree Neither agree nor disagree Disagree Strongly disagree Not applicable I don't know Prefer not to say  Rows: Doctors and other healthcare professionals understand their OI You are provided with sufficient information about OI You are provided with sufficient information on how to care for your child Treatments and treatment options are explained to you sufficiently Treatments and treatment options are explained to your child appropriately Doctors and other healthcare professionals understand your concerns Doctors and other healthcare professional understand your child's needs and concerns The care is continuous The care is coordinated You feel you need to coordinate your child's care yourself | No | - | - | x | - | - | - | - | - | - |
| 232 | Please indicate how you feel the following statements describe your child's situation since the start of the COVID-19 pandemic. | Please select one answer per row | Only one answer per row | Columns: Strongly agree Agree Neither agree nor disagree Disagree Strongly disagree Not applicable I don't know Prefer not to say  Rows: Your child has had fewer appointments with healthcare providers Your child has had fewer medical tests and examinations Appointments that previously took place in person are now online/on the phone Your child has been able to access additional healthcare because more doctors offer phone/online appointments You have avoided seeking medical care for your child because you are worried about exposure to COVID-19 You have avoided visiting the emergency department for your child because of COVID-19 | No | - | - | x | - | - | - | - | - | - |
| 233 | Do you sometimes avoid seeking medical care for your child? | Please select only one answer | No | Yes No I don't know Prefer not to say | No | - | - | x | - | - | - | - | - | - |
| 234 | Please indicate whether you avoid seeking medical care for your child due to any of the reasons below: | Please select one answer per row | Only one answer per row | Columns: Strongly agree Agree Neither agree nor disagree Disagree Strongly disagree Not applicable I don't know Prefer not to say  Rows: High costs Because of past trauma or negative experiences you or your child had You can do it better yourself Your child's healthcare provider is too far away from your home You don't have transportation to access your child's healthcare provider easily Fear Healthcare professionals in your area are not familiar with OI You don't trust healthcare professionals   - Other reasons: | Yes | - | - | x | - | - | - | - | - | - |
| 235 | At what age (years) were your children diagnosed with OI? | If you are not sure, please give your best estimate | No | Column: Age (years at diagnosis)  Prenatally At birth ˂1 Integer (1 to 18) I don't know Prefer not to say Rows: Child 1 Child 2 | No | - | - | x | - | - | - | - | - | - |
| 236 | Do any of the following apply to you? | Please all that apply for each child | Yes | Columns: Child 1 Child 2 Rows: My child was initially diagnosed with a different condition(s) My child's OI was initially suspected as child abuse None of the above Prefer not to say | No | - | - | - | x | - | - | - | - | - |
| 237 | At what age (years) were your children diagnosed with OI? | If you are not sure, please give your best estimate | No | Column: Age (years at diagnosis)  Prenatally At birth ˂1 Integer (1 to 18) I don't know Prefer not to say Rows: Child 1 Child 2 Child 3 | No | - | - | - | x | - | - | - | - | - |
| 238 | Do any of the following apply to you? | Please all that apply for each child | Yes | Columns: Child 1 Child 2 Child 3 Rows: My child was initially diagnosed with a different condition(s) My child's OI was initially suspected as child abuse None of the above Prefer not to say | No | - | - | - | - | x | - | - | - | - |
| 239 | Which condition(s) was child 1 diagnosed with? | Please write the name of the condition(s) your child was diagnosed with. If you don't know, please write 'I don't know'. | Free text |  | Yes | - | - | - | - | x | - | - | - | - |
| 240 | Which condition(s) was child 1 diagnosed with? | Please write the name of the condition(s) your child was diagnosed with. If you don't know, please write 'I don't know'. | Free text |  | Yes | - | - | - | x | - | - | - | - | - |
| 241 | Which condition(s) was child 2 diagnosed with? | Please write the name of the condition(s) your child was diagnosed with. If you don't know, please write 'I don't know'. | Free text |  | Yes | - | - | - | - | x | - | - | - | - |
| 242 | Which condition(s) was child 2 diagnosed with? | Please write the name of the condition(s) your child was diagnosed with. If you don't know, please write 'I don't know'. | Free text |  | Yes | - | - | - | x | - | - | - | - | - |
| 243 | Which condition(s) was child 3 diagnosed with? | Please write the name of the condition(s) your child was diagnosed with. If you don't know, please write 'I don't know'. | Free text |  | Yes | - | - | - | - | x | - | - | - | - |
| 244 | How long did it take from your children's first fracture to receiving an OI diagnosis | None | No | Column: Time until diagnosis  My child received their diagnosis before their first fracture My child received their diagnosis straight away <3 months 3-6 months 7-12 months 13-24 months 2-5 years 6-10 years More than 10 years I'm not sure Prefer not to say  Rows: Child 1 Child 2 | No | - | - | - | - | x | - | - | - | - |
| 245 | How has diagnosis impacted child 1's journey through the healthcare system? | Please select one answer per row | Only one answer per row | Columns: Strongly agree Agree Neither agree nor disagree Disagree Strongly disagree Not applicable I don't know Prefer not to say Rows: I am satisfied with the length of time taken for my child's OI diagnosis I am satisfied with the diagnostic services that were available to my child My child has received more tailored support and care after their diagnosis My child has had better access to specialist OI services after their diagnosis I feel I am better able to manage my child's condition after their diagnosis | No | - | - | - | x | - | - | - | - | - |
| 246 | How has diagnosis impacted child 2's journey through the healthcare system? | Please select one answer per row | Only one answer per row | Columns: Strongly agree Agree Neither agree nor disagree Disagree Strongly disagree Not applicable I don't know Prefer not to say  Rows: I am satisfied with the length of time taken for my child's OI diagnosis I am satisfied with the diagnostic services that were available to my child My child has received more tailored support and care after their diagnosis My child has had better access to specialist OI services after their diagnosis I feel I am better able to manage my child's condition after their diagnosis | No | - | - | - | x | - | - | - | - | - |
| 247 | How long did it take from your children's first fracture to receiving an OI diagnosis? | None | No | Column: Time until diagnosis  My child received their diagnosis before their first fracture My child received their diagnosis straight away <3 months 3-6 months 7-12 months 13-24 months 2-5 years 6-10 years More than 10 years I'm not sure Prefer not to say  Rows: Child 1 Child 2 Child 3 | No | - | - | - | x | - | - | - | - | - |
| 248 | How has diagnosis impacted child 1's journey through the healthcare system? | Please select one answer per row | Only one answer per row | Columns: Strongly agree Agree Neither agree nor disagree Disagree Strongly disagree Not applicable I don't know Prefer not to say Rows: I am satisfied with the length of time taken for my child's OI diagnosis I am satisfied with the diagnostic services that were available to my child My child has received more tailored support and care after their diagnosis My child has had better access to specialist OI services after their diagnosis I feel I am better able to manage my child's condition after their diagnosis | No | - | - | - | - | x | - | - | - | - |
| 249 | How has diagnosis impacted child 2's journey through the healthcare system? | Please select one answer per row | Only one answer per row | Columns: Strongly agree Agree Neither agree nor disagree Disagree Strongly disagree Not applicable I don't know Prefer not to say Rows: I am satisfied with the length of time taken for my child's OI diagnosis I am satisfied with the diagnostic services that were available to my child My child has received more tailored support and care after their diagnosis My child has had better access to specialist OI services after their diagnosis I feel I am better able to manage my child's condition after their diagnosis | No | - | - | - | - | x | - | - | - | - |
| 250 | How has diagnosis impacted child 3's journey through the healthcare system? | Please select one answer per row | Only one answer per row | Columns: Strongly agree Agree Neither agree nor disagree Disagree Strongly disagree Not applicable I don't know Prefer not to say Rows: I am satisfied with the length of time taken for my child's OI diagnosis I am satisfied with the diagnostic services that were available to my child My child has received more tailored support and care after their diagnosis My child has had better access to specialist OI services after their diagnosis I feel I am better able to manage my child's condition after their diagnosis | No | - | - | - | - | x | - | - | - | - |
| 251 | Over the past 12 months, has child 1 experienced any of the following signs, symptoms, or events? | Please select all that apply | Yes | Pain Fractures Fatigue Scoliosis or other bone problems Soft tissue problems or injuries (muscles, tendons, ligaments) Hypermobility Joint problems (dislocations, osteoarthritis) Hearing problems Eye or vision problems Dental problems Lung or breathing problems Stomach and bowel problems (for example, constipation, diarrhoea) Kidney and bladder problems (for example, kidney stones, urinary tract infection, incontinence) High blood pressure Heart problems (for example, atrial fibrillation/flutter, valve regurgitation, heart attack, arrhythmia) Sleep disturbance Depression, anxiety, or other mental health problems Basilar invagination (which may include pain in the back of the head or upper neck, trouble talking or swallowing, confusion, feeling dizzy or lightheaded) Gynaecological problems/menstruation problems Problems with chewing, swallowing, and speaking Obesity/overweight Low/underweight  None of the above  Prefer not to say  - Other signs and symptoms child 2 has experienced | Yes | - | - | - | - | x | - | - | - | - |
| 252 | Over the past 12 months, has child 2 experienced any of the following signs, symptoms, or events? | Please select all that apply | Yes | Pain Fractures Fatigue Scoliosis or other bone problems Soft tissue problems or injuries (muscles, tendons, ligaments) Hypermobility Joint problems (dislocations, osteoarthritis) Hearing problems Eye or vision problems Dental problems Lung or breathing problems Stomach and bowel problems (for example, constipation, diarrhoea) Kidney and bladder problems (for example, kidney stones, urinary tract infection, incontinence) High blood pressure Heart problems (for example, atrial fibrillation/flutter, valve regurgitation, heart attack, arrhythmia) Sleep disturbance Depression, anxiety, or other mental health problems Basilar invagination (which may include pain in the back of the head or upper neck, trouble talking or swallowing, confusion, feeling dizzy or lightheaded) Gynaecological problems/menstruation problems Problems with chewing, swallowing, and speaking Obesity/overweight Low/underweight  None of the above  Prefer not to say  - Other signs and symptoms child 1 has experienced | Yes | - | - | - | x | x | - | - | - | - |
| 253 | Over the past 12 months, has child 3 experienced any of the following signs, symptoms, or events? | Please select all that apply | Yes | Pain Fractures Fatigue Scoliosis or other bone problems Soft tissue problems or injuries (muscles, tendons, ligaments) Hypermobility Joint problems (dislocations, osteoarthritis) Hearing problems Eye or vision problems Dental problems Lung or breathing problems Stomach and bowel problems (for example, constipation, diarrhoea) Kidney and bladder problems (for example, kidney stones, urinary tract infection, incontinence) High blood pressure Heart problems (for example, atrial fibrillation/flutter, valve regurgitation, heart attack, arrhythmia) Sleep disturbance Depression, anxiety, or other mental health problems Basilar invagination (which may include pain in the back of the head or upper neck, trouble talking or swallowing, confusion, feeling dizzy or lightheaded) Gynaecological problems/menstruation problems Problems with chewing, swallowing, and speaking Obesity/overweight Low/underweight  None of the above  Prefer not to say  - Other signs and symptoms child 2 has experienced | Yes | - | - | - | x | x | - | - | - | - |
| 254 | In the past 12 months, how many times has child 1 visited hospital? | Please include all visits, for any reason, including OI-unrelated visits. This includes both planned and emergency visits. If you are not sure, please give your best estimate. | No | Prefer not to say Integer (0 to 100) | No | - | - | - | - | x | - | - | - | - |
| 255 | In the past 12 months, how many times has child 2 visited hospital? | Please include all visits, for any reason, including OI-unrelated visits. This includes both planned and emergency visits. If you are not sure, please give your best estimate. | No | Prefer not to say Integer (0 to 100) | No | - | - | - | x | - | - | - | - | - |
| 256 | In the past 12 months, how many times has child 1 visited hospital? | Please include all visits, for any reason, including OI-unrelated visits. This includes both planned and emergency visits. If you are not sure, please give your best estimate. | No | Prefer not to say Integer (0 to 100) | No | - | - | - | x | - | - | - | - | - |
| 257 | In the past 12 months, how many times has child 2 visited hospital? | Please include all visits, for any reason, including OI-unrelated visits. This includes both planned and emergency visits. If you are not sure, please give your best estimate. | No | Prefer not to say Integer (0 to 100) | No | - | - | - | - | x | - | - | - | - |
| 258 | In the past 12 months, how many times has child 3 visited hospital? | Please include all visits, for any reason, including OI-unrelated visits. This includes both planned and emergency visits. If you are not sure, please give your best estimate. | No | Prefer not to say Integer (0 to 100) | No | - | - | - | - | x | - | - | - | - |
| 259 | Of these times (in the past 12 months), how many times has child 1 visited an emergency department? | Please include all visits, for any reason, including OI-unrelated visits. If you are not sure, please give your best estimate. | No | Prefer not to say Integer (0 to 100) | No | - | - | - | - | x | - | - | - | - |
| 260 | Of these times (in the past 12 months), how many times has child 2 visited an emergency department? | Please include all visits, for any reason, including OI-unrelated visits. If you are not sure, please give your best estimate. | No | Prefer not to say Integer (0 to 100) | No | - | - | - | x | x | - | - | - | - |
| 261 | Of these times (in the past 12 months), how many times has child 3 visited an emergency department? | Please include all visits, for any reason, including OI-unrelated visits. If you are not sure, please give your best estimate. | No | Prefer not to say Integer (0 to 100) | No | - | - | - | x | x | - | - | - | - |
| 262 | In the past 12 months, how many nights did child 1 spend in hospital overall (for both planned and emergency visits)? | Please include all visits, for any reason, including OI-unrelated visits. If you are not sure, please give your best estimate | No | Prefer not to say Integer (0 to 100) | No | - | - | - | - | x | - | - | - | - |
| 263 | In the past 12 months, how many nights did child 2 spend in hospital overall (for both planned and emergency visits)? | Please include all visits, for any reason, including OI-unrelated visits. If you are not sure, please give your best estimate | No | Prefer not to say Integer (0 to 100) | No | - | - | - | x | x | - | - | - | - |
| 264 | In the past 12 months, how many nights did child 3 spend in hospital overall (for both planned and emergency visits)? | Please include all visits, for any reason, including OI-unrelated visits. If you are not sure, please give your best estimate | No | Prefer not to say Integer (0 to 100) | No | - | - | - | x | x | - | - | - | - |
| 265 | In the past 12 months, how many nights has your children spent in a rehabilitation facility? | Please include all visits, for any reason, including OI-unrelated visits. If you are not sure, please give your best estimate. | No | Column: Number of nights:  Not applicable Prefer not to say Integer (0 to 100) Rows: Child 1 Child 2 | No | - | - | - | - | x | - | - | - | - |
| 266 | In the past 12 months, how many times has your children received the following tests and examinations? | Please fill in those that apply to your children. Please include any tests and examinations, including OI-unrelated procedures. For those that do not apply, please leave blank. If you are not sure, please give your best estimate. | Yes | Columns: Child 1  Integer (1 to 49) >50 I don't know Prefer not to say Child 2  Integer (1 to 49) >50 I don't know  Prefer not to say Rows: Blood test Urine test X-ray CT scan (computed tomography) DEXA scan (dual energy X-ray absorptiometry) MRI scan (magnetic resonance imaging) Ultrasound scan Echo scan (echocardiogram, heart scan) Audiology (hearing) test  - Other (please specify) | Yes | - | - | - | x | - | - | - | - | - |
| 267 | In the past 12 months, how many nights has your children spent in a rehabilitation facility? | Please include all visits, for any reason, including OI-unrelated visits. If you are not sure, please give your best estimate. | No | Column: Number of nights:  Not applicable Prefer not to say Integer (0 to 100) Rows: Child 1 Child 2 Child 3 | No | - | - | - | x | - | - | - | - | - |
| 268 | In the past 12 months, how many times has your children received the following tests and examinations? | Please fill in those that apply to your children. Please include any tests and examinations, including OI-unrelated procedures. For those that do not apply, please leave blank. If you are not sure, please give your best estimate. | Yes | Columns: Child 1  Integer (1 to 49) >50 I don't know Prefer not to say Child 2  Integer (1 to 49) >50 I don't know  Prefer not to say Rows: Blood test Urine test X-ray CT scan (computed tomography) DEXA scan (dual energy X-ray absorptiometry) MRI scan (magnetic resonance imaging) Ultrasound scan Echo scan (echocardiogram, heart scan) Audiology (hearing) test  - Other (please specify) | Yes | - | - | - | - | x | - | - | - | - |
| 269 | Please indicate how often your children have visited the following healthcare professionals in the past 12 months. | Please fill in those that apply to your children. For those that do not apply, please leave blank. If you are not sure, please give your best estimate. Please also include appointments that took place over the phone or video chat. | Yes | Columns: Child 1  More than twice weekly Twice weekly Weekly Every other week Monthly Every six weeks Every other month Once per quarter Once every six months Once per year Child 2  More than twice weekly Twice weekly Weekly Every other week Monthly Every six weeks Every other month Once per quarter Once every six months Once per year Rows: General practitioner/family doctor Nurse practitioner/care coordinator Paediatrician (children's doctor) Orthopaedic surgeon/orthopaedist (bone specialist) Nutritionist Psychotherapist/counsellor Physiotherapist Dentist/orthodontist Audiology (hearing) Ophthalmologist (eye) Gynaecologist/obstetrician (women's reproductive health) Endocrinologist (hormones, bone turnover/density) Cardiologist (heart) Neurologist (brain and nervous system) Gastroenterologist (digestive system) Rheumatologist (musculoskeletal system) Pulmonologist (lung/respiratory system) Rehabilitation therapist/doctor Occupational therapist (helps to recover, improve, and maintain skills needed for daily living and working)   - If your children have visited any other healthcare professionals, please specify below: | Yes | - | - | - | - | x | - | - | - | - |
| 270 | Have your children visited any of the following healthcare professionals in the past (prior to the past 12 months)? | Please select all that apply to your children | Yes | Columns: Child 1 Child 2 Rows: General practitioner/family doctor Nurse practitioner/care coordinator Paediatrician (children's doctor) Orthopaedic surgeon/orthopaedist (bone specialist) Nutritionist Psychotherapist/counsellor Physiotherapist Dentist/orthodontist Audiology (hearing) Ophthalmologist (eye) Gynaecologist/obstetrician (women's reproductive health) Endocrinologist (hormones, bone turnover/density) Cardiologist (heart) Neurologist (brain and nervous system) Gastroenterologist (digestive system) Rheumatologist (musculoskeletal system) Pulmonologist (lung/respiratory system) Rehabilitation therapist/doctor Occupational therapist (helps to recover, improve, and maintain skills needed for daily living and working)  - If your children have visited any other healthcare professionals in the past, please specify below: | Yes | - | - | - | x | - | - | - | - | - |
| 271 | Does your children use pain medication regularly? | Please select one answer for each child | No | Columns: Yes No Prefer not to say  Rows: Child 1 Child 2 | No | - | - | - | x | - | - | - | - | - |
| 272 | Over the past 4 weeks, how often (on average) have your children used pain medication? | If you are not sure, please give your best estimate. | No | Column: Frequency  Daily Several times per week Once per week Less than once per week They haven't used painkillers in the last 4 weeks I don't know Prefer not to say Rows: Child 1 Child 2 | No | - | - | - | x | - | - | - | - | - |
| 273 | Please indicate how often your children have visited the following healthcare professionals in the past 12 months. | Please fill in those that apply to your children. For those that do not apply, please leave blank. If you are not sure, please give your best estimate. Please also include appointments that took place over the phone or video chat. | Yes | Columns: Child 1  More than twice weekly Twice weekly Weekly Every other week Monthly Every six weeks Every other month Once per quarter Once every six months Once per year Child 2  More than twice weekly Twice weekly Weekly Every other week Monthly Every six weeks Every other month Once per quarter Once every six months Once per year  Child 3  More than twice weekly Twice weekly Weekly Every other week Monthly Every six weeks Every other month Once per quarter Once every six months Once per year Rows: General practitioner/family doctor Nurse practitioner/care coordinator Paediatrician (children's doctor) Orthopaedic surgeon/orthopaedist (bone specialist) Nutritionist Psychotherapist/counsellor Physiotherapist Dentist/orthodontist Audiology (hearing) Ophthalmologist (eye) Gynaecologist/obstetrician (women's reproductive health) Endocrinologist (hormones, bone turnover/density) Cardiologist (heart) Neurologist (brain and nervous system) Gastroenterologist (digestive system) Rheumatologist (musculoskeletal system) Pulmonologist (lung/respiratory system) Rehabilitation therapist/doctor Occupational therapist (helps to recover, improve, and maintain skills needed for daily living and working)  - If your children have visited any other healthcare professionals, please specify below: | Yes | - | - | - | x | - | - | - | - | - |
| 274 | Have your children visited any of the following healthcare professionals in the past (prior to the past 12 months)? | Please select all that apply to your children | Yes | Columns: Child 1 Child 2 Child 3 Rows: General practitioner/family doctor Nurse practitioner/care coordinator Paediatrician (children's doctor) Orthopaedic surgeon/orthopaedist (bone specialist) Nutritionist Psychotherapist/counsellor Physiotherapist Dentist/orthodontist Audiology (hearing) Ophthalmologist (eye) Gynaecologist/obstetrician (women's reproductive health) Endocrinologist (hormones, bone turnover/density) Cardiologist (heart) Neurologist (brain and nervous system) Gastroenterologist (digestive system) Rheumatologist (musculoskeletal system) Pulmonologist (lung/respiratory system) Rehabilitation therapist/doctor Occupational therapist (helps to recover, improve, and maintain skills needed for daily living and working)  - If your children have visited any other healthcare professionals in the past, please specify below: | Yes | - | - | - | - | x | - | - | - | - |
| 275 | Does your children use pain medication regularly? | Please select one answer for each child | No | Columns: Yes No Prefer not to say  Rows: Child 1 Child 2 Child 3 | No | - | - | - | - | x | - | - | - | - |
| 276 | Over the past 4 weeks, how often (on average) have your children used pain medication? | If you are not sure, please give your best estimate. | No | Column: Frequency  Daily Several times per week Once per week Less than once per week They haven't used painkillers in the last 4 weeks I don't know Prefer not to say Rows: Child 1 Child 2 Child 3 | No | - | - | - | - | x | - | - | - | - |
| 277 | What kinds of other OI treatments have your children taken in the past 12 months? | Please select all that apply for each child. If your children take other treatments not listed below, please specify them in the comments box. | Yes | Columns: Child 1 Child 2 Rows: Bisphosphonates Vitamin D supplements Calcium supplements Muscle relaxants Parathyroid hormone (PTH) (for example, abaloparatide [Tymlos®], teriparatide [Forteo®]) Oestrogen hormone (for example, raloxifene [Evista®], tamoxifen [Nolvadex®]) Antibodies (for example, denosumab [Prolia®], romosozumab [Evenity®], setrusumab) None of the above   - Other treatments (please specify): | Yes | - | - | - | - | x | - | - | - | - |
| 278 | What kinds of other OI treatments have your children taken in the past 12 months? | Please select all that apply for each child. If your children take other treatments not listed below, please specify them in the comments box. | Yes | Columns: Child 1 Child 2 Child 3 Rows: Bisphosphonates Vitamin D supplements Calcium supplements Muscle relaxants Parathyroid hormone (PTH) (for example, abaloparatide [Tymlos®], teriparatide [Forteo®]) Oestrogen hormone (for example, raloxifene [Evista®], tamoxifen [Nolvadex®]) Antibodies (for example, denosumab [Prolia®], romosozumab [Evenity®], setrusumab) None of the above   - Other treatments (please specify): | Yes | - | - | - | x | - | - | - | - | - |
| 279 | What type of bisphosphonates does child 1 currently take? |  | No | Pamidronate (Aredia®) Alendronate (Fosamax®, Fosavance®) Zoledronate (Zometa®, Reclast®, Aclasta®) Risedronate (Actonel®, Atelvia®, Benet®, Ribastamin®) Ibandronate (Boniva®, Bonviva®, Bondronat®, Lasibon®, Quodixor®) Neridronate (Nerixia®, Attilia®) Clodronate (Bonefos®, Clasteon®, Loron®, Sindronat®, Lodronat®) Etidronate (Didronel®) Other (please specify in the comment box) I don't know Prefer not to say   - Other (please specify) | Yes | - | - | - | - | x | - | - | - | - |
| 280 | Following bisphosphonate treatment, how do you feel things have changed for child 1? | Please select one answer per row | Only one answer per row | Columns: Improved Stayed the same Got worse Not applicable I don't know Prefer not to say  Rows: Growth rate Mobility Fatigue Pain How easily they fracture Bone mass   - Other (please specify) | Yes | - | - | - | x | - | - | - | - | - |
| 281 | Following bisphosphonate treatment, has child 1 experienced any of the following? | Please select all that apply | Yes | Osteonecrosis of the jaw (which may include symptoms like delayed healing, exposed bone, swelling) Sudden hearing loss Stomach problems They have not experienced any of the listed issues Prefer not to say Other (please specify)   - Other (please specify): | Yes | - | - | - | x | - | - | - | - | - |
| 282 | What type of bisphosphonates does child 1 currently take? |  | No | Pamidronate (Aredia®) Alendronate (Fosamax®, Fosavance®) Zoledronate (Zometa®, Reclast®, Aclasta®) Risedronate (Actonel®, Atelvia®, Benet®, Ribastamin®) Ibandronate (Boniva®, Bonviva®, Bondronat®, Lasibon®, Quodixor®) Neridronate (Nerixia®, Attilia®) Clodronate (Bonefos®, Clasteon®, Loron®, Sindronat®, Lodronat®) Etidronate (Didronel®) Other (please specify in the comment box) I don't know Prefer not to say   - Other (please specify) | Yes | - | - | - | x | - | - | - | - | - |
| 283 | Following bisphosphonate treatment, how do you feel things have changed for child 1? | Please select one answer per row | Only one answer per row | Columns: Improved Stayed the same Got worse Not applicable I don't know Prefer not to say  Rows: Growth rate Mobility Fatigue Pain How easily they fracture Bone mass   - Other (please specify) | Yes | - | - | - | - | x | - | - | - | - |
| 284 | Following bisphosphonate treatment, has child 1 experienced any of the following? | Please select all that apply | Yes | Osteonecrosis of the jaw (which may include symptoms like delayed healing, exposed bone, swelling) Sudden hearing loss Stomach problems They have not experienced any of the listed issues Prefer not to say Other (please specify)   - Other (please specify): | Yes | - | - | - | - | x | - | - | - | - |
| 285 | What type of bisphosphonates does child 2 currently take? |  | No | Pamidronate (Aredia®) Alendronate (Fosamax®, Fosavance®) Zoledronate (Zometa®, Reclast®, Aclasta®) Risedronate (Actonel®, Atelvia®, Benet®, Ribastamin®) Ibandronate (Boniva®, Bonviva®, Bondronat®, Lasibon®, Quodixor®) Neridronate (Nerixia®, Attilia®) Clodronate (Bonefos®, Clasteon®, Loron®, Sindronat®, Lodronat®)Etidronate (Didronel®) Other (please specify in the comment box) I don't know Prefer not to say   - Other (please specify) | Yes | - | - | - | - | x | - | - | - | - |
| 286 | Following bisphosphonate treatment, how do you feel things have changed for child 2? | Please select one answer per row | Only one answer per row | Columns: Improved Stayed the same Got worse Not applicable I don't know Prefer not to say Rows: Growth rate Mobility Fatigue Pain How easily they fracture Bone mass   - Other (please specify) | Yes | - | - | - | x | - | - | - | - | - |
| 287 | Following bisphosphonate treatment, has child 2 experienced any of the following? | Please select all that apply | Yes | Osteonecrosis of the jaw (which may include symptoms like delayed healing, exposed bone, swelling) Sudden hearing loss Stomach problems They have not experienced any of the listed issues Prefer not to say Other (please specify)  - Other (please specify): | Yes | - | - | - | x | - | - | - | - | - |
| 288 | What type of bisphosphonates does child 2 currently take? |  | No | Pamidronate (Aredia®) Alendronate (Fosamax®, Fosavance®) Zoledronate (Zometa®, Reclast®, Aclasta®) Risedronate (Actonel®, Atelvia®, Benet®, Ribastamin®) Ibandronate (Boniva®, Bonviva®, Bondronat®, Lasibon®, Quodixor®) Neridronate (Nerixia®, Attilia®) Clodronate (Bonefos®, Clasteon®, Loron®, Sindronat®, Lodronat®)Etidronate (Didronel®) Other (please specify in the comment box) I don't know Prefer not to say   - Other (please specify) | Yes | - | - | - | x | - | - | - | - | - |
| 289 | Following bisphosphonate treatment, how do you feel things have changed for child 2? | Please select one answer per row | Only one answer per row | Columns: Improved Stayed the same Got worse Not applicable I don't know Prefer not to say Rows: Growth rate Mobility Fatigue Pain How easily they fracture Bone mass   - Other (please specify) | Yes | - | - | - | - | x | - | - | - | - |
| 290 | Following bisphosphonate treatment, has child 2 experienced any of the following? | Please select all that apply | Yes | Osteonecrosis of the jaw (which may include symptoms like delayed healing, exposed bone, swelling) Sudden hearing loss Stomach problems They have not experienced any of the listed issues Prefer not to say Other (please specify)  - Other (please specify): | Yes | - | - | - | - | x | - | - | - | - |
| 291 | What type of bisphosphonates does child 3 currently take? |  | No | Pamidronate (Aredia®) Alendronate (Fosamax®, Fosavance®) Zoledronate (Zometa®, Reclast®, Aclasta®) Risedronate (Actonel®, Atelvia®, Benet®, Ribastamin®) Ibandronate (Boniva®, Bonviva®, Bondronat®, Lasibon®, Quodixor®) Neridronate (Nerixia®, Attilia®) Clodronate (Bonefos®, Clasteon®, Loron®, Sindronat®, Lodronat®)Etidronate (Didronel®) Other (please specify in the comment box) I don't know Prefer not to say   - Other (please specify) | Yes | - | - | - | - | x | - | - | - | - |
| 292 | Following bisphosphonate treatment, how do you feel things have changed for child 3? | Please select one answer per row | Only one answer per row | Columns: Improved Stayed the same Got worse Not applicable I don't know Prefer not to say Rows: Growth rate Mobility Fatigue Pain How easily they fracture Bone mass   - Other (please specify) | Yes | - | - | - | - | x | - | - | - | - |
| 293 | Following bisphosphonate treatment, has child 3 experienced any of the following? | Please select all that apply | Yes | Osteonecrosis of the jaw (which may include symptoms like delayed healing, exposed bone, swelling) Sudden hearing loss Stomach problems They have not experienced any of the listed issues Prefer not to say Other (please specify)  - Other (please specify): | Yes | - | - | - | - | x | - | - | - | - |
| 294 | In your children's lives, how many surgeries have they had for the following things? | Please include surgeries for any reason, including OI-unrelated reasons. Please fill in those that apply to your children. For those that do not apply, please leave blank. If you are not sure, give your best estimate. | Yes | Columns: Child 1  Integer (1 to 49) >50 I don't know Prefer not to say Child 2  Integer (1 to 49) >50 I don't know Prefer not to say Rows: Rodding Fracture repairs Spine Hearing Teeth Heart Tonsils and adenoids Basilar invagination (skull/neck) Soft tissue (for example, tendons, hypermobility, dislocations) Other (please specify below)  - Other (please specify) | Yes | - | - | - | - | x | - | - | - | - |
| 295 | In your children's lives, how many surgeries have they had for the following things? | Please include surgeries for any reason, including OI-unrelated reasons. Please fill in those that apply to your children. For those that do not apply, please leave blank. If you are not sure, give your best estimate. | Yes | Columns: Child 1  Integer (1 to 49) >50 I don't know Prefer not to say Child 2  Integer (1 to 49) >50 I don't know Prefer not to say  Child 3  Integer (1 to 49) >50 I don't know Prefer not to say Rows: Rodding Fracture repairs Spine Hearing Teeth Heart Tonsils and adenoids Basilar invagination (skull/neck) Soft tissue (for example, tendons, hypermobility, dislocations) Other (please specify below)  - Other (please specify) | Yes | - | - | - | x | - | - | - | - | - |
| 296 | Overall, how do you feel about the following aspects of your child's OI treatment and care? | Please select one answer per row | Only one answer per row | Columns: Strongly agree Agree Neither agree nor disagree Disagree Strongly disagree Not applicable I don't know Prefer not to say Rows: Doctors and other healthcare professionals understand their OI You are provided with sufficient information about OI You are provided with sufficient information on how to care for your children Treatments and treatment options are explained to you sufficiently Treatments and treatment options are explained to your children appropriately Doctors and other healthcare professionals understand your concerns Doctors and other healthcare professionals understand your children's needs and concerns The care is continuous The care is coordinated You feel you need to coordinate your children's care yourself | No | - | - | - | - | x | - | - | - | - |
| 297 | Please indicate how you feel the following statements describe your children's situation since the start of the COVID-19 pandemic. | Please select one answer per row | Only one answer per row | Columns: Strongly agree Agree Neither agree nor disagree Disagree Strongly disagree Not applicable I don't know Prefer not to say Rows: Your children have received less healthcare Appointments that previously took place in person are now online/on the phone Your children has been able to access additional healthcare because more doctors offer phone/online appointments You have avoided seeking medical care for your children because you are worried about exposure to COVID-19 You have avoided visiting the emergency department for your children because of COVID-19 | No | - | - | - | x | x | - | - | - | - |
| 298 | Do you sometimes avoid seeking medical care for your children? | Please select only one answer | No | Yes No I don't know Prefer not to say | No | - | - | - | x | x | - | - | - | - |
| 299 | Please indicate whether you avoid seeking medical care for your children due to any of the reasons below: | Please select one answer per row | Only one answer per row | Columns: Strongly agree Agree Neither agree nor disagree Disagree Strongly disagree Not applicable I don't know Prefer not to say Rows: High costs Because of past trauma or negative experiences you or your children had You can do it better yourself Your children's healthcare provider is too far away from your home You don't have transportation to access your children's healthcare provider easily Fear Healthcare professionals in your area are not familiar with OI You don't trust healthcare professionals  - Other reasons: | Yes | - | - | - | x | x | - | - | - | - |
| 300 | If there are any positive impacts of OI you would like to describe, please use the space below. | None | Only free text | - | Yes | - | - | - | x | x | - | - | - | - |
| 301 | How did you hear about the IMPACT survey? |  | No | Internet search (search engine) Email Facebook Instagram Twitter Other social medial platform Friends/family OIFE communication OIF communication Communication from your national OI organisation Conference/meeting Flyer Poster Other (please specify below)  - Other (please specify): | Yes | - | - | x | x | x | - | - | - | - |
| 302 | What is your country of residence? |  | No | Afghanistan – Zimbabwe (195 alternatives)  Prefer not to say | No | - | x | x | x | x | - | - | - | - |
| 303 | What is your sex? | Please select only one answer | No | Male Female Other Prefer not to say | No | - | x | - | - | - | - | - | - | - |
| 304 | Please indicate which of the following best describe you. | Please select only one answer | No | I attend my school of choice I attend school, but not my school of choice I attend a special school I am home schooled Prefer not to say Other (please specify):   - Other (please specify): | Yes | - | x | - | - | - | - | - | - | - |
| 305 | In the last 4 weeks school took place, how many days of school have you missed because of your OI? | Please note: you may have not visited school in the past 4 weeks due to summer holidays or COVID-19. Please respond about the last 4 weeks your school was open. | No | 0 Less than 1 Integer (1 to 28) I don't know Prefer not to say | No | - | x | - | - | - | - | - | - | - |
| 306 | Who do you live with? | Please select only one answer | No | I live with my parents (and other family members if applicable) I live with one of my parents (and other family members if applicable) I live with my grandparent/grandparents (and other family members if applicable) I live with my caregiver/assistant I live in supported living accommodation or a care home Prefer not to say Other (please specify):   - Other (please specify): | Yes | - | x | - | - | - | - | - | - | - |
| 307 | Do you prefer the metric or imperial system to describe your height? | Please select only one answer | No | Metric (centimetres) Imperial (feet/inches) | No | - | x | - | - | - | - | - | - | - |
| 308 | What is your height? | Please select your height in centimetres | No | I don't know Prefer not to say Integer (40 to 200) | No | - | x | - | - | - | - | - | - | - |
| 309 | What is your height? | Please select your height in feet and inches | No | I don't know Prefer not to say Integer (1 feet, 4 inches to 6 feet, 8 inches) | No | - | x | - | - | - | - | - | - | - |
| 310 | How do you get around? | Please select all that apply | Multiple answers per row | Columns: Inside your home Outside your home  Rows: Walking unaided Cane/walking stick Rollator (wheeled walker) Walking frame Crutches Manual wheelchair Powered wheelchair Mobility scooter Crawling Being carried Laying in bed/stretcher Other (please specify below)   - Other: | Yes | - | x | - | - | - | - | - | - | - |
| 311 | If you have received an OI type as part of your OI diagnosis or treatment, please indicate your type using the dropdown below. | Please note: If you were not diagnosed with a specific type, please select 'Undefined type', if you do not know your type please select 'I don't know'. You may have been diagnosed with a specific OI type even if you did not have genetic testing. In all cases we will use your responses to other questions in this survey to understand more about the kind of OI you have. | No | Undefined type I don't know Prefer not to say Type 1 (I) - Type 18 (XVIII) Other   - Other (please specify) | Yes | - | x | - | - | - | - | - | - | - |
| 312 | How would you describe the severity of your OI? | Please select only one answer | No | Mild Moderate Severe I don't know Prefer not to say | No | - | x | - | - | - | - | - | - | - |
| 313 | Do you have a genetically confirmed diagnosis of OI? | Please select only one answer | No | Yes No I don't know Prefer not to say | No | - | x | - | - | - | - | - | - | - |
| 314 | Why is your OI not genetically confirmed? | Please select all that apply | Yes | My test was inconclusive I do not want a test The test was too expensive for me I wasn't offered a test I don't know Prefer not to say For other reasons (please specify):   - For other reasons (please specify): | Yes | - | x | - | - | - | - | - | - | - |
| 315 | Which gene is the cause of your OI diagnosis? |  | No | BMP1 COL1A1 COL1A2 CREB3L1 CRTAP FKBP10 IFITM5 LEPRE1/P3H1 MBTPS2 MESD P4HB PLOD2 PLS3 PPIB SEC24D SERPINF1 SERPINH1 SP7 SPARC TENT5A TMEM38 WNT1 Other I don't know Prefer not to say   - Other (please specify) | Yes | - | x | - | - | - | - | - | - | - |
| 316 | In the past 12 months, how would you describe the impact that OI has had on your life? | This question is about understanding the 'negative' impacts or challenges you have faced. We will ask you about any positive impacts later in the survey, Please select one answer per row. | Only one answer per row | Columns: Severely impacted Moderately impacted Mildly impacted Very mildly impacted Not impacted I don't know Prefer not to say  Rows: The number of school hours you miss Your career choices Your ability to care for yourself (for example, dressing, bathing) The type of leisure activities you can do (for example, hobbies, sports, reading) Your social life (socialising with friends, participating in activities) Your relationships with family Your relationships with friends Your mental health Your happiness   - Are there any other aspects of your life that you feel are impacted by OI? | Yes | - | x | - | - | - | - | - | - | - |
| 317 | At what age (years) were you diagnosed with OI? | If you are not sure, please give your best estimate. | No | I don't know Prenatally At birth <1 Integer (1 to 100) | No | - | x | - | - | - | - | - | - | - |
| 318 | Over the past 12 months, have you experienced any of the following signs, symptoms, or events? | Please select all that apply | Yes | Pain Fractures Fatigue (feeling tired) Spine and backbone curvature problems (scoliosis) or other bone problems Soft tissue problems or injuries (muscles, tendons, ligaments) Hypermobility Joint problems (dislocations, osteoarthritis) Hearing problems Eye or vision problems Dental problems Lung or breathing problems Stomach and bowel problems (for example, constipation, diarrhoea) Kidney and bladder problems (for example, kidney stones, urinary tract infection, incontinence) High blood pressure Heart problems (for example, atrial fibrillation/flutter, valve regurgitation, heart attack, arrhythmia) Sleep disturbance Depression, anxiety, or other mental health problems Basilar invagination (which may include pain in the back of the head or upper neck, trouble talking or swallowing, confusion, feeling dizzy or lightheaded) Gynaecological problems/menstruation problems Problems with chewing, swallowing, and speaking Obesity/overweight Low/underweight None of the above Prefer not to say   - Other signs and symptoms you have experienced | Yes | - | x | - | - | - | - | - | - | - |
| 319 | In the past 12 months, how has pain impacted your life? | This question is referring to pain of any type. Please select only one answer. | No | Severely Moderately Mildly Very mildly Not at all I don't know Prefer not to say | No | - | x | - | - | - | - | - | - | - |
| 320 | How would you describe the type and frequency of pain you have experienced in the past 12 months? | Please include all types of pain you experience and select the frequency of each type from the dropdown menus. If you do not experience a type/severity of pain you may leave that field blank. | Yes - several drop down menus per row | Columns: Mild   Every day Often Sometimes Rarely Never Moderate  Every day Often Sometimes Rarely Never Severe  Every day Often Sometimes Rarely Never  Rows: Pain all over your body from nowhere specific Sharp, stabbing, shooting, electric shocks that radiate out Dull, aching, throbbing, squeezing deep inside (from internal organ or non-specific areas) Dull, aching, throbbing in your joints, bones, or muscles Burning, stinging, numbness, tingling, pricking from a specific area   - Other (please specify): | Yes | - | x | - | - | - | - | - | - | - |
| 321 | In the past 12 months, how have fractures impacted your life? | Please select only one answer | No | Severely Moderately Mildly Very mildly Not at all I don't know Prefer not to say | No | - | x | - | - | - | - | - | - | - |
| 322 | In the past 12 months, how many times have you experienced any of the following events? | Please use the dropdown menu to indicate the number of times you have experienced each event. If you have not experienced the event in the past 12 months, please select '0'. | Only one answer per row | Columns: Number of times  Prefer not to say Integer (0 to 50) >50  Rows: Arm fractures Leg fractures Vertebral fractures Rib fractures Other fractures (for example, pelvis, skull, fingers, toes, sternum; please specify below)   - Please specify which other fractures you have experienced | Yes | - | x | - | - | - | - | - | - | - |
| 323 | In the past 12 months, how has fatigue (feeling tired) impacted your life? | Please select only one answer | No | Severely Moderately Mildly Very mildly Not at all I don't know Prefer not to say | No | - | x | - | - | - | - | - | - | - |
| 324 | In the past 12 months, how have spine and backbone curvature problems (scoliosis) or other bone problems impacted your life? | Please select only one answer | No | Severely Moderately Mildly Very mildly Not at all I don't know Prefer not to say | No | - | x | - | - | - | - | - | - | - |
| 325 | In the past 12 months, how have soft tissue (muscles, tendons, ligaments) problems impacted your life? | Please select only one answer | No | Severely Moderately Mildly Very mildly Not at all I don't know Prefer not to say | No | - | x | - | - | - | - | - | - | - |
| 326 | In the past 12 months, how many times have you experienced soft tissue injuries (muscles, tendons, ligaments)? | Please use the dropdown menu to indicate the number of times you have experienced soft tissue injuries. If you have not experienced the event in the past 12 months, please select '0'. | No | Integer (0-50) I don't know Prefer not to say | No | - | x | - | - | - | - | - | - | - |
| 327 | In the past 12 months, how has hypermobility impacted your life? | Please select only one answer | No | Severely Moderately Mildly Very mildly Not at all I don't know Prefer not to say | No | - | x | - | - | - | - | - | - | - |
| 328 | In the past 12 months, how have joint problems (for example dislocations or osteoarthritis) impacted your life? | Please select only one answer | No | Severely Moderately Mildly Very mildly Not at all I don't know Prefer not to say | No | - | x | - | - | - | - | - | - | - |
| 329 | In the past 12 months, how have hearing problems impacted your life? | Please select only one answer | No | Severely Moderately Mildly Very mildly Not at all I don't know Prefer not to say | No | - | x | - | - | - | - | - | - | - |
| 330 | In the past 12 months, how have eye or vision problems impacted your life? | Please select only one answer | No | Severely Moderately Mildly Very mildly Not at all I don't know Prefer not to say | No | - | x | - | - | - | - | - | - | - |
| 331 | In the past 12 months, how have dental problems impacted your life? | Please select only one answer | No | Severely Moderately Mildly Very mildly Not at all I don't know Prefer not to say | No | - | x | - | - | - | - | - | - | - |
| 332 | In the past 12 months, how have lung or breathing problems impacted your life? | Please select only one answer | No | Severely Moderately Mildly Very mildly Not at all I don't know Prefer not to say | No | - | x | - | - | - | - | - | - | - |
| 333 | In the past 12 months, how have stomach and bowel problems (for example constipation or diarrhoea) impacted your life? | Please select only one answer | No | Severely Moderately Mildly Very mildly Not at all I don't know Prefer not to say | No | - | x | - | - | - | - | - | - | - |
| 334 | In the past 12 months, how have kidney and bladder problems impacted your life? | Please select only one answer | No | Severely Moderately Mildly Very mildly Not at all I don't know Prefer not to say | No | - | x | - | - | - | - | - | - | - |
| 335 | In the past 12 months, how has high blood pressure impacted your life? | Please select only one answer | No | Severely Moderately Mildly Very mildly Not at all I don't know Prefer not to say | No | - | x | - | - | - | - | - | - | - |
| 336 | In the past 12 months, how have heart events (for example heart attacks, arrhythmia) impacted your life? | Please select only one answer | No | Severely Moderately Mildly Very mildly Not at all I don't know Prefer not to say | No | - | x | - | - | - | - | - | - | - |
| 337 | In the past 12 months, how many times have you experienced cardiac events (such as heart attacks, arrhythmia)? | Please use the dropdown menu to indicate the number of times you have experienced cardiac events. If you have not experienced cardiac event in the past 12 months, please select '0'. | No | Integer (0-50) I don't know Prefer not to say | No | - | x | - | - | - | - | - | - | - |
| 338 | In the past 12 months, how has sleep disturbance impacted your life? | Please select only one answer | No | Severely Moderately Mildly Very mildly Not at all I don't know Prefer not to say | No | - | x | - | - | - | - | - | - | - |
| 339 | In the past 12 months, how have depression/anxiety or other mental health problems impacted your life? | Please select only one answer | No | Severely Moderately Mildly Very mildly Not at all I don't know Prefer not to say | No | - | x | - | - | - | - | - | - | - |
| 340 | In the past 12 months, how has basilar invagination (which may include pain in the back of the head or upper neck, trouble talking or swallowing, confusion, feeling dizzy or lightheaded) impacted your life? | Select only one answer | No | Severely Moderately Mildly Very mildly Not at all I don't know Prefer not to say | No | - | x | - | - | - | - | - | - | - |
| 341 | In the past 12 months, how have gynaecological problems/menstruation problems impacted your life? | Please select only one answer | No | Severely Moderately Mildly Very mildly Not at all I don't know Prefer not to say | No | - | x | - | - | - | - | - | - | - |
| 342 | In the past 12 months, how have problems with chewing, swallowing, and speaking impacted your life? | Please select only one answer | No | Severely Moderately Mildly Very mildly Not at all I don't know Prefer not to say | No | - | x | - | - | - | - | - | - | - |
| 343 | In the past 12 months, how have obesity/overweight problems impacted your life? | Please select only one answer | No | Severely Moderately Mildly Very mildly Not at all I don't know Prefer not to say | No | - | x | - | - | - | - | - | - | - |
| 344 | In the past 12 months, how have problems with low/underweight impacted your life? | Please select only one answer | No | Severely Moderately Mildly Very mildly Not at all I don't know Prefer not to say | No | - | x | - | - | - | - | - | - | - |
| 345 | In the past 12 months, how have mobility challenges impacted your life? | Please select only one answer | No | Severely Moderately Mildly Very mildly Not at all I don't know Prefer not to say | No | - | x | - | - | - | - | - | - | - |
| 346 | Prior to the past 12 months (during your lifetime), have you experienced any of the following signs, symptoms, or events? | Please select all that apply | Yes | Pain Fractures Fatigue (feeling tired) Spine and backbone curvature problems (scoliosis) or other bone problems Soft tissue problems or injuries (muscles, tendons, ligaments) Hypermobility Joint problems (dislocations, osteoarthritis) Hearing problems Eye or vision problems Dental problems Lung or breathing problems Stomach and bowel problems (for example, constipation, diarrhoea) Kidney and bladder problems (for example, kidney stones, urinary tract infection, incontinence) High blood pressure Heart problems (for example, atrial fibrillation/flutter, valve regurgitation, heart attack, arrhythmia) Sleep disturbance Depression, anxiety, or other mental health problems Basilar invagination (which may include pain in the back of the head or upper neck, trouble talking or swallowing, confusion, feeling dizzy or lightheaded) Gynaecological  problems/menstruation problems Problems with chewing, swallowing, and speaking Obesity/overweight Low/underweight None of the above  Prefer not to say  - Other signs and symptoms you have experienced | Yes | - | x | - | - | - | - | - | - | - |
| 347 | In the past 12 months, how many times have you visited hospital? | Please include all visits, for any reason, including OI-unrelated visits. This includes both planned and emergency visits. If you are not sure, please give your best estimate. | No | Prefer not to say Integer (0 to 100) | No | - | x | - | - | - | - | - | - | - |
| 348 | Of these times (in the past 12 months), how many times have you visited the emergency department? | Please include all visits, for any reason, including OI-unrelated visits. If you are not sure, please give your best estimate. | No | Prefer not to say Integer (0 to 100) | No | - | x | - | - | - | - | - | - | - |
| 349 | In the past 12 months how many nights did you spend in hospital overall (for both planned and emergency visits)? | Please include all visits, for any reason, including OI-unrelated visits. If you are not sure, please give your best estimate. | No | Prefer not to say Integer (0 to 100) | No | - | x | - | - | - | - | - | - | - |
| 350 | In the past 12 months, how many nights did you spend in a rehabilitation facility? | Please include all visits, for any reason, including OI-unrelated visits. If you are not sure, please give your best estimate. | No | Not applicable Prefer not to say Integer (0 to 100) | No | - | x | - | - | - | - | - | - | - |
| 351 | In the past 12 months, how many times have your received the following tests and examinations? | Please include any tests and examinations, including OI-unrelated procedures. If you are not sure, please give your best estimate. If you have not received any of these tests in the past 12 months, please select '0'. | Only one answer per row | Columns: Number of times  I don't know Prefer not to say Integer (0 to 49) >50  Rows: Blood test Urine test X-ray CT scan (computed tomography) DEXA scan (dual energy X-ray absorptiometry) MRI scan (magnetic resonance imaging) Ultrasound scan Echo scan (echocardiogram, heart scan) Audiology (hearing) test Other (please specify)   - Please specify which other tests you have received | Yes | - | x | - | - | - | - | - | - | - |
| 352 | Please indicate how often you have visited the following healthcare professionals in the past 12 months. | Please include all visits, for any reason, including OI-unrelated visits. If you are not sure, please give your best estimate. Please also include appointments that took place over the phone or video chat. Please leave blank if you have not seen a healthcare professional. | Only one answer per row | Columns: More than twice weekly Twice weekly Weekly Every other week Monthly Every six weeks Every other month Once per quarter Once every six months Once per year  Rows: General practitioner/family doctor Nurse practitioner/care coordinator Paediatrician (children's doctor) Orthopaedic surgeon/orthopaedist (bone specialist) Nutritionist Psychotherapist/counsellor Physiotherapist Dentist/orthodontist Audiology (hearing) Ophthalmologist (eye) Gynaecologist/obstetrician (women's reproductive health) Endocrinologist (hormones, bone turnover/density) Cardiologist (heart) Neurologist (brain and nervous system) Gastroenterologist (digestive system) Rheumatologist (musculoskeletal system) Pulmonologist (lung/respiratory system) Rehabilitation therapist/doctor Occupational therapist (helps to recover, improve, and maintain skills needed for daily living and working)   - If you have visited any other healthcare professionals, please specify below: | Yes | - | x | - | - | - | - | - | - | - |
| 353 | Have you visited any of the following healthcare professionals in the past (prior to the past 12 months)? | Please include all visits, for any reason, including OI-unrelated visits. Please select all that apply | Yes | General practitioner/family doctor Nurse practitioner/care coordinator Paediatrician (children's doctor) Orthopaedic surgeon/orthopaedist (bone specialist) Nutritionist Psychotherapist/counsellor Physiotherapist Dentist/orthodontist Audiology (hearing) Ophthalmologist (eye) Gynaecologist/obstetrician (women's reproductive health) Endocrinologist (hormones, bone turnover/density) Cardiologist (heart) Neurologist (brain and nervous system) Gastroenterologist (digestive system) Rheumatologist (musculoskeletal system) Pulmonologist (lung/respiratory system) Rehabilitation therapist/doctor Occupational therapist (helps to recover, improve, and maintain skills needed for daily living and working) None of the above Other healthcare professionals you have visited in the past (please specify below)   - Other healthcare professionals you have visited in the past (please specify below) | Yes | - | x | - | - | - | - | - | - | - |
| 354 | Do you use pain medication regularly? | Please select only one answer | No | Yes No Prefer not to say | No | - | x | - | - | - | - | - | - | - |
| 355 | Over the past 4 weeks, how often (on average) have you used pain medication? | If you are not sure, give your best estimate. | No | Daily Several times per week Once per week Less than once per week I haven't used painkillers in the last 4 weeks I don't know Prefer not to say | No | - | x | - | - | - | - | - | - | - |
| 356 | What kinds of other OI treatment are you taking now, or have you taken before? | Please select one answer per row. If you take other treatments not listed below, please specify them in the comments box. | Only one answer per row | Columns: I currently take this treatment (in the past 12 months) I used to take this treatment (prior to the past 12 months) I have never taken this treatment I don't know Prefer not to say  Rows: Bisphosphonates Vitamin D supplements Calcium supplements Muscle relaxants Parathyroid hormone (PTH) (for example, abaloparatide [Tymlos®], teriparatide [Forteo®]) Oestrogen hormone (for example, raloxifene [Evista®], tamoxifen [Nolvadex®]) Antibodies (for example, denosumab [Prolia®], romosozumab [Evenity®], setrusumab)   - Other treatments (please specify): | Yes | - | x | - | - | - | - | - | - | - |
| 357 | What type of bisphosphonates do you currently take? | None | No | Pamidronate (Aredia®) Alendronate (Fosamax®, Fosavance®) Zoledronate (Zometa®, Reclast®, Aclasta®) Risedronate (Actonel®, Atelvia®, Benet®, Ribastamin®) Ibandronate (Boniva®, Bonviva®, Bondronat®, Lasibon®, Quodixor®) Neridronate (Nerixia®, Attilia®) Clodronate (Bonefos®, Clasteon®, Loron®, Sindronat®, Lodronat®) Etidronate (Didronel®) Other (please specify in the comment box) I don't know Prefer not to say   - Other (please specify) | Yes | - | x | - | - | - | - | - | - | - |
| 358 | Following bisphosphonate treatment, how do you feel things have changed for you? | Please select one answer per row | Only one answer per row | Columns: Improved Stayed the same Got worse Not applicable I don't know Prefer not to say Rows: Growth rate Mobility Fatigue Pain How easily you fracture Strength and thickness of your bones (bone mass)  - Other (please specify): | Yes | - | x | - | - | - | - | - | - | - |
| 359 | Following bisphosphonate treatment, have you experienced any of the following? | Please select all that apply | Yes | Osteonecrosis of the jaw (which may include symptoms like delayed healing, exposed bone, swelling) Sudden hearing loss Stomach problems I have not experienced any of the listed issues Prefer not to say Other (please specify)  - Other (please specify): | Yes | - | x | - | - | - | - | - | - | - |
| 360 | In your life, how many surgeries have you had for the following things? | Please include all surgeries for any reason, including OI-unrelated reasons. If you are not sure, please give your best estimate. For those that do not apply, please select '0'. | Only one answer per row | Columns: Number of surgeries  I don't know Prefer not to say Integer (0 to 50) Rows: Rodding Fracture repairs Spine Hearing Teeth Heart Basilar invagination (skull/neck) Soft tissue (for example, tendons, hypermobility, dislocations) Other (please specify below)  - Please specify which other surgeries you have had. | Yes | - | x | - | - | - | - | - | - | - |
| 361 | Overall, how do you feel about the following aspects of your OI treatment and care? | Please select one answer per row | Only one answer per row | Columns: Strongly agree Agree Neither agree nor disagree Disagree Strongly disagree Not applicable I don't know Prefer not to say Rows: Doctors and other healthcare professionals understand your condition You are provided with sufficient information about your OI You have access to the treatment and care that you need You have access to regular follow up Treatments and treatment options are explained to you sufficiently Doctors and other healthcare professionals understand your needs and concerns You feel you are/will be supported in your transition from childhood doctors to adult care | No | - | x | - | - | - | - | - | - | - |
| 362 | Please indicate whether you agree with the following statements about how your healthcare might have changed since the start of the COVID-19 pandemic. | Please select one answer per row | Only one answer per row | Columns: Severely Moderately Mildly Very mildly Not at all I don't know Prefer not to say  Rows: You have had fewer appointments with healthcare providers You have had fewer medical tests and examinations Appointments that previously took place in person are now online/on the phone You have been able to access additional healthcare because more doctors offer phone/online appointments You have avoided seeking medical care because you are worried about exposure to COVID-19 You have avoided visiting the emergency department because of COVID-19 | No | - | x | - | - | - | - | - | - | - |
| 363 | Do you sometimes avoid seeking medical care for reasons other than the COVID-19 pandemic? | Please select only one answer | No | Yes No I don't know Prefer not to say | No | - | x | - | - | - | - | - | - | - |
| 364 | Please indicate whether you avoid seeking medical care due to any of the reasons below: | Please select one answer per row | Only one answer per row | Columns: Severely Moderately Mildly Very mildly Not at all I don't know Prefer not to say  Rows: Because of past trauma or negative experiences You/your parents can do it better Your healthcare provider is too far away from your home You don't have transportation to access your healthcare provider easily Fear Healthcare professionals in your area are not familiar with OI You don't trust healthcare professionals  - Other reasons: | Yes | - | x | - | - | - | - | - | - | - |
| 365 | Do you require any of the following? | Please select all that apply | Yes | Manual wheelchair Powered wheelchair Walking aids (for example crutches, walking frame) Hearing aids Breathing aid/machine (for example CPAP [continuous positive airway pressure therapy] machine) Home modifications (for example wheelchair ramps and other changes for easy access) Parent/caregiver vehicle modifications (for example for easier wheelchair access to the vehicle) Modifications at work (for example wheelchair ramps and other changes for easy access) Personal care/support assistance Dental work None of the above   - If there are any other things you use (excluding medicines and hospital care), you can use the space below to describe them | Yes | - | x | - | - | - | - | - | - | - |
| 366 | If there are any positive impacts of OI you would like to describe, please use the space below. | None | Only free text |  | Yes | x | x | - | - | - | - | - | - | - |
| 367 | How did you hear about the IMPACT survey? |  | No | Internet search (search engine) Email Facebook Instagram Twitter Other social medial platform Friends/family OIFE communication OIF communication Conference/meeting Flyer Poster National organisation Other (please specify below)   - Other (please specify): | Yes | x | x | - | - | - | x | x | x | - |
| 368 | Do you have a close relationship to a person with OI? | NONE | No | Yes No | No | - | - | - | - | - | x | x | x | - |
| 369 | Please indicate which of the following best describes your relationship to the person with OI | Please select only one answer | No | Child of a person with OI Sibling of a person with OI Grandparent of a person with OI Partner of a person with OI Other relationship to a person with OI (please specify below)   - Other relationship to a person with OI (please specify below) | Yes | - | - | - | - | - | - | - | - | x |
| 370 | What is your country of residence? |  | No | Afghanistan-Zimbabwe (**195 alternatives)**  Prefer not to say | No | - | - | - | - | - | - | - | - | x |
| 371 | What is your sex? | Please select only one answer | No | Male Female Other Prefer not to say | No | - | - | - | - | - | - | - | - | x |
| 372 | Please indicate which of the following best describe you. | Please select all that apply to you | Yes | I am in paid employment full time I am in paid employment part time I am self-employed I am in full time education I am in part time education I am retired I am a homemaker (housewife/househusband) I am a volunteer I am between jobs I am unable to find a job I am not employed I am not fit to work Prefer not to say Other (please specify):   - Other (please specify): | Yes | - | - | - | - | - | - | - | - | x |
| 373 | Do you live with a person with OI? |  | No | Yes No Prefer not to say | No | - | - | - | - | - | - | - | - | x |
| 374 | In the past 12 months, how would you describe the impact that OI has had on your life? | This question is about understanding the ‘negative’ impacts or challenges you have faced. We will ask you about any positive impacts later in the survey. Please select one answer per row. | Only one answer per row | Columns: Severely impacted Moderately impacted Mildly impacted Very mildly impacted Not impacted Not applicable I don't know Prefer not to say Rows: The number of hours you work in your paid job The type of job you can do Your career choices The amount of time you have free for leisure activities (for example, hobbies, sports, reading) The type of leisure activities you can do (for example, hobbies, sports, reading) Your social life (for example, socialising with friends, attending events, participating in activities) Your relationships with family and friends Your relationship with the person with OI Your romantic relationships Your mental health Your physical health Your happiness   - Are there any other aspects of your life that you feel are impacted by OI? | Yes | - | - | - | - | - | - | - | - | x |
| 375 | Do you feel worried or concerned about any of the following things in relation to the person with OI? | Please select one answer per row. In this question 'they/their' refers to the person with OI. | Only one answer per row | Columns: Worry a lot Worry a little  Don't worry Not applicable I don't know Prefer not to say Rows: Their future Their opportunities How their OI impact your other family members How their OI impacts your relationships with family and friends How their OI impacts your romantic relationships The quality of care they have access to The amount of support they will require from you in the future Your financial situation in the future   - Do you have any other worries or concerns? | Yes | - | - | - | - | - | - | - | - | x |
| 376 | If there are any positive impacts of OI you would like to describe, please use the space below. |  | Only free text |  | Yes | - | - | - | - | - | - | - | - | x |
| 377 | How did you hear about the IMPACT survey? |  | No | Internet search (search engine) Email Facebook Instagram Twitter Other social medial platform Friends/family OIFE communication OIF communication Conference/meeting Flyer Poster National organisation Other (please specify below)   - Other (please specify): | Yes | - | - | - | - | - | - | - | - | x |
